# Risk of Operative and Nonoperative Interventions Up to 4 Years After Roux-en-Y Gastric Bypass vs Vertical Sleeve Gastrectomy in a Nationwide US Commercial Insurance Claims Database

**DOI:** 10.1001/jamanetworkopen.2019.17603

**Published:** 2019-12-18

**Authors:** Kristina H. Lewis, David E. Arterburn, Katherine Callaway, Fang Zhang, Stephanie Argetsinger, Jamie Wallace, Adolfo Fernandez, Dennis Ross-Degnan, James F. Wharam

**Affiliations:** 1 Division of Public Health Sciences, Wake Forest School of Medicine, Winston-Salem, North Carolina; 2 Department of General Surgery, Wake Forest School of Medicine, Winston-Salem, North Carolina; 3 Kaiser Permanente Washington Health Research Institute, Seattle, Washington; 4 Division of Health Policy and Insurance Research, Department of Population Medicine, Harvard Pilgrim Healthcare Institute, Harvard Medical School, Boston, Massachusetts

## Abstract

**Importance:**

There are few nationwide studies comparing the risk of reintervention after contemporary bariatric procedures.

**Objective:**

To compare the risk of intervention after Roux-en-Y gastric bypass (RYGB) vs vertical sleeve gastrectomy (VSG).

**Design, Setting, and Participants:**

This cohort study used a nationwide US commercial insurance claims database. Adults aged 18 to 64 years who underwent a first RYGB or VSG procedure between January 1, 2010, and June 30, 2017, were matched on US region, year of surgery, most recent presurgery body mass index (BMI) category (based on diagnosis codes), and baseline type 2 diabetes. The prematch pool included 4496 patients undergoing RYGB and 8627 patients undergoing VSG, and the final weighted matched sample included 4476 patients undergoing RYGB and 8551 patients undergoing VSG.

**Exposures:**

Bariatric surgery procedure type (RYGB vs VSG).

**Main Outcomes and Measures:**

The primary outcome was any abdominal operative intervention after the index procedure. Secondary outcomes included the following subtypes of operative intervention: biliary procedures, abdominal wall hernia repair, bariatric conversion or revision, and other abdominal operations. Nonoperative outcomes included endoscopy and enteral access. Time to first event was compared using multivariable Cox proportional hazards regression modeling.

**Results:**

Among 13 027 patients, the mean (SD) age was 44.4 (10.3) years, and 74.1% were female; 13.7% had a preoperative BMI between 30 and 39.9, 45.8% had a preoperative BMI between 40 and 49.9, and 24.2% had a preoperative BMI of at least 50. Patients were followed up for up to 4 years after surgery (median, 1.6 years; interquartile range, 0.7-3.2 years), with 41.9% having at least 2 years of follow-up and 16.3% having at least 4 years of follow-up. Patients undergoing VSG were less likely to have any subsequent operative intervention than matched patients undergoing RYGB (adjusted hazard ratio [aHR], 0.80; 95% CI, 0.72-0.89) and similarly were less likely to undergo biliary procedures (aHR, 0.77; 95% CI, 0.67-0.90), abdominal wall hernia repair (aHR, 0.60; 95% CI, 0.47-0.75), other abdominal operations (aHR, 0.71; 95% CI, 0.61-0.82), and endoscopy (aHR, 0.54; 95% CI, 0.49-0.59) or have enteral access placed (aHR, 0.58; 95% CI, 0.39-0.86). Patients undergoing VSG were more likely to undergo bariatric conversion or revision (aHR, 1.83; 95% CI, 1.19-2.80).

**Conclusions and Relevance:**

In this nationwide study, patients undergoing VSG appeared to be less likely than matched patients undergoing RYGB to experience subsequent abdominal operative interventions, except for bariatric conversion or revision procedures. Patients considering bariatric surgery should be aware of the increased risk of subsequent procedures associated with RYGB vs VSG as part of shared decision-making around procedure choice.

## Key Points

**Question:**

Do patients have greater risk of subsequent operative intervention after Roux-en-Y gastric bypass (RYGB) or vertical sleeve gastrectomy (VSG)?

**Findings:**

Among 13 027 patients in this cohort study, compared with matched counterparts undergoing RYGB, patients undergoing VSG had lower overall risk of subsequent operative and nonoperative interventions, including biliary procedures, abdominal wall hernia repair, other abdominal operations, and endoscopy, up to 4 years after surgery.

**Meaning:**

Patients deciding between RYGB and VSG should consider not only the clinical effectiveness of these 2 procedures but also the risk for subsequent operative and nonoperative interventions.

## Teaser

This cohort study compares the risk of operative and nonoperative interventions up to 4 years after Roux-en-Y gastric bypass vs vertical sleeve gastrectomy in patient groups matched on US region, year of surgery, body mass index category, and type 2 diabetes status.

## Introduction

Operative reintervention is among the most concerning sequelae of bariatric surgery. It can occur within days or weeks because of problems like anastomotic leak, infection, or hemorrhage^1,2^ or years later, with revisional procedures performed to enhance weight loss or address chronic complications.^3^ Return trips to the operating room have been associated with higher complication rates than primary bariatric surgical procedures^4,5,6^ and are thus critical outcomes for patients to consider as they select an initial procedure.

Differences in reintervention rates between Roux-en-Y gastric bypass (RYGB) and vertical sleeve gastrectomy (VSG), the most common contemporary bariatric procedures, have been understudied.^1,7,8,9^ Most relevant prior comparative studies have examined reoperation rates by 30 days^10,11,12^ or 1 to 2 years after an index procedure.^13,14,15^ Analyses with longer follow-up time have been characterized by small sample sizes that limit assessment of operation subtypes^16,17^ or were single-center studies,^18,19^ limiting generalizability. Another potential limitation of prior observational studies of reoperation is reliance on clinical registry or electronic health record data,^14,18,20,21^ leading to incomplete capture of subsequent surgical procedures performed outside of the health system under study. Two randomized clinical trials^17,22^ found no statistically significant differences in reoperation rates between RYGB and VSG at 5 years; however, it is unclear whether these findings could be generalized to larger, more heterogeneous populations. Finally, the relative frequency of nonsurgical interventions, such as endoscopy, has not been well characterized after RYGB vs VSG. The present analysis uses a nationwide US commercial insurance claims database to compare matched cohorts of patients undergoing RYGB vs patients undergoing VSG with respect to subsequent abdominal operative interventions (AOIs), as well as subcategories of operations and invasive but nonoperative interventions, up to 4 years after an index procedure.

## Methods

### Study Design and Data Source

This retrospective cohort study was conducted using a nationwide US commercial insurance claims deidentified database (Optum’s deidentified Clinformatics Data Mart). This database includes inpatient, outpatient, and pharmacy claims from January 1, 2010, to June 30, 2017, for approximately 33 million members of a large national health plan, as well as enrollment and demographic information. The study was approved by the Harvard Pilgrim Institutional Review Board with a waiver of patient informed consent. The data-only nature of the present analysis posed minimal risks to participating individuals. We minimized the only potential risk of loss of confidentiality of medical data by presenting all results in aggregate. This study followed the Strengthening the Reporting of Observational Studies in Epidemiology (STROBE) reporting guideline.

### Study Population

We identified adult members aged 18 to 64 years who underwent a first RYGB or VSG procedure between January 1, 2010, and June 30, 2017. Detailed methods for identifying these patients were previously published.^23^ Briefly, *Current Procedural Terminology* (*CPT*) codes and *International Classification of Diseases, Ninth Revision* (*ICD-9*) and *International Statistical Classification of Diseases and Related Health Problems, Tenth Revision *(*ICD-10*) procedure codes were used to identify the earliest bariatric procedure for each patient in the data set. The analysis was restricted to patients whose index procedure was coded as a laparoscopic RYGB or VSG because open procedures would have different risks of subsequent reintervention. Patients with diagnosis codes that suggested surgical indications other than obesity were excluded. Patients in the RYGB and VSG groups were required to have at least 6 months of insurance enrollment before surgery to allow assessment of baseline comorbidities. Patients had a minimum of 1-month postoperative insurance enrollment. They were followed up to 4 years after surgery and censored at death, gastrointestinal cancer diagnosis, age 65 years, or insurance disenrollment (true loss to follow-up).

### Outcome Measures

The primary outcome measure was subsequent AOI after the date of the index procedure. This comprehensive outcome included any operative procedure on the abdomen that could be associated with the index bariatric procedure. We first developed a listing of more than 1000 relevant *CPT*, *ICD-9*, and *ICD-10* codes for AOI based on abdominal procedures that were identified in the data set, as well as in collaboration with 2 other research teams examining similar outcomes in different data sets (eTable 1 in the Supplement). Surgical procedures on the large intestine (eg, colectomy) and inguinal and femoral hernia repairs were excluded because these were unlikely to be related to the bariatric procedure.

In addition to the overall AOI outcome, several subcategories of surgical intervention were examined separately. These interventions included biliary procedures (eg, cholecystectomy), abdominal wall hernia repair, bariatric conversion or revision (eg, RYGB or gastrectomy), and other abdominal operations for presumed complication (eg, drain abscess, stricturoplasty, and paraesophageal hernia repair). Complete *CPT*, *ICD-9*, and *ICD-10* codes are listed in eTable 1 in the Supplement.

In clinical practice, procedures like paraesophageal^24^ or ventral^25^ hernia repair and cholecystectomy^26^ are sometimes performed concurrently with an index bariatric procedure. Insurance claims for these concurrent procedures might appear on a later date, leading to overcounting in the early period after the index procedure. Therefore, a 30-day postindex washout period was applied for codes representing common concurrent procedures (eTable 2 in the Supplement), during which these procedures were not counted as unique operations.

Several types of nonoperative interventions after bariatric surgery were examined, including endoscopy, enteral access (eg, placement of a percutaneous enterogastrostomy tube), and other nonoperative interventions (eg, paracentesis or radiology-guided drainage of the abdomen). These outcomes were also characterized using *CPT*, *ICD-9*, and *ICD-10* codes (eTable 1 in the Supplement), and no washout period was used.

### Covariates

Demographic measures included age group (18-39, 40-49, 50-59, and 60-64 years), sex, and US region (West, South, Midwest, and Northeast). Because race/ethnicity is associated with operative outcomes,^27^ we classified patients as residing in predominantly white, black, mixed-race, Hispanic, or Asian neighborhoods based on geocoding.^28,29^ Validated measures of neighborhood poverty and educational level^29,30^ were created using 2010 US Census tract–level data,^31,32^ which defined patients as living in low-income neighborhoods when at least 10% of people in their area were below the poverty line and as living in less educated neighborhoods when the percentage of adults lacking a high school diploma was at least 25%.^33^ Timing of procedure was grouped in 2-year blocks from 2010 to 2017.

Presurgery body mass index (BMI) (calculated as weight in kilograms divided by height in meters squared) was determined based on the most recently coded diagnosis in the baseline 6 months. Because BMI diagnoses in claims data do not provide exact values, patients were categorized into the following groups: BMI of 30 to 39.9, 40 to 49.9, 50 to 59.9, 60 or higher, nonspecific obesity (when only a generic obesity code, such as *ICD-9* code 278.01, was available), and missing (<1%).^23^

Johns Hopkins Adjusted Clinical Groups (ACG) System software^34,35^ was used to calculate an overall measure of morbidity based on medical and pharmacy claims in the baseline 6 months, and patients were classified as having lower morbidity (score, <3) or higher morbidity (score, ≥3). The ACG software was also used to flag the specific comorbidities of hypertension, cardiovascular disease, and mental illness. Patients with type 2 diabetes, gastroesophageal reflux disease (GERD), and history of tobacco use were identified based on the presence of diagnosis codes for these conditions in the 6 months before surgery (eTable 3 in the Supplement).

### Matching Strategy

To ensure that RYGB and VSG cohorts were balanced on characteristics that could be associated with future procedures, coarsened exact matching (CEM) was conducted^36,37,38^ within categories of selected variables, creating weights for each stratum that adjusted for differences between study groups in the proportion of persons in the stratum (akin to stratified randomization in a trial). We exactly matched on variables with baseline standardized differences of at least 0.1 (absolute value), including US region, year of surgery, preoperative BMI category, and type 2 diabetes status. For each variable, we matched patients undergoing RYGB with missing values to those in the VSG group who were also missing.

### Statistical Analysis

#### Analytic Approach

A standardized differences approach was used to compare baseline characteristics of the RYGB group and VSG group before and after matching. If the standardized difference was less than 0.2 (absolute value), the groups were deemed to be well balanced.^39^

For each outcome measure, separate CEM-weighted Kaplan-Meier curves with 95% CIs were constructed to visualize each outcome in a time-to-event framework. From these curves, the percentage of patients in each group with an event at key time points during follow-up were estimated, accounting for their matching weights. To compare the cumulative risk of an outcome between RYGB and VSG, Cox proportional hazards regression models were built for each outcome of interest. The models were adjusted for all matched covariates, plus age group, sex, baseline ACG comorbidity score group, and presence of hypertension, GERD, and mental illness. In each Cox proportional hazards regression model, patients who did not have a qualifying event for that particular outcome were censored at the time of incident gastrointestinal cancer, death, the end of our data set (June 30, 2017), or plan disenrollment (loss to follow-up).

#### Sensitivity Analyses

Because *ICD-9* and *ICD-10* procedure codes are more likely to be generated by hospitals than by clinicians, it is possible that they are less accurate than *CPT* codes. Therefore, all analyses were repeated using outcome definitions based solely on *CPT* codes.

Despite the advantage of CEM for generating groups that are well balanced on key variables, the results of a recent simulation study^40^ suggest that this approach may confer a higher risk of type I error than ordinary least squares regression modeling. To test whether the present study’s results were sensitive to the approach for handling potential confounders, all Cox proportional hazards regression models were repeated on the raw unmatched cohort of 4496 patients undergoing RYGB and 8627 patients undergoing VSG, adjusting for the matching variable used in the main analysis. Statistical significance was assessed at a significance level of *P* < .05 using 2-sided tests and 95% CIs. All data pulls were performed in SAS Studio, version 3.7 (SAS Institute Inc). We used Stata, version 16.0 (StataCorp) to complete the CEM matching and Cox proportional hazards regressions and R Studio, version 3.6.0 (R Foundation) to construct the Kaplan-Meier curves.

## Results

### Study Population

**Figure 1.**
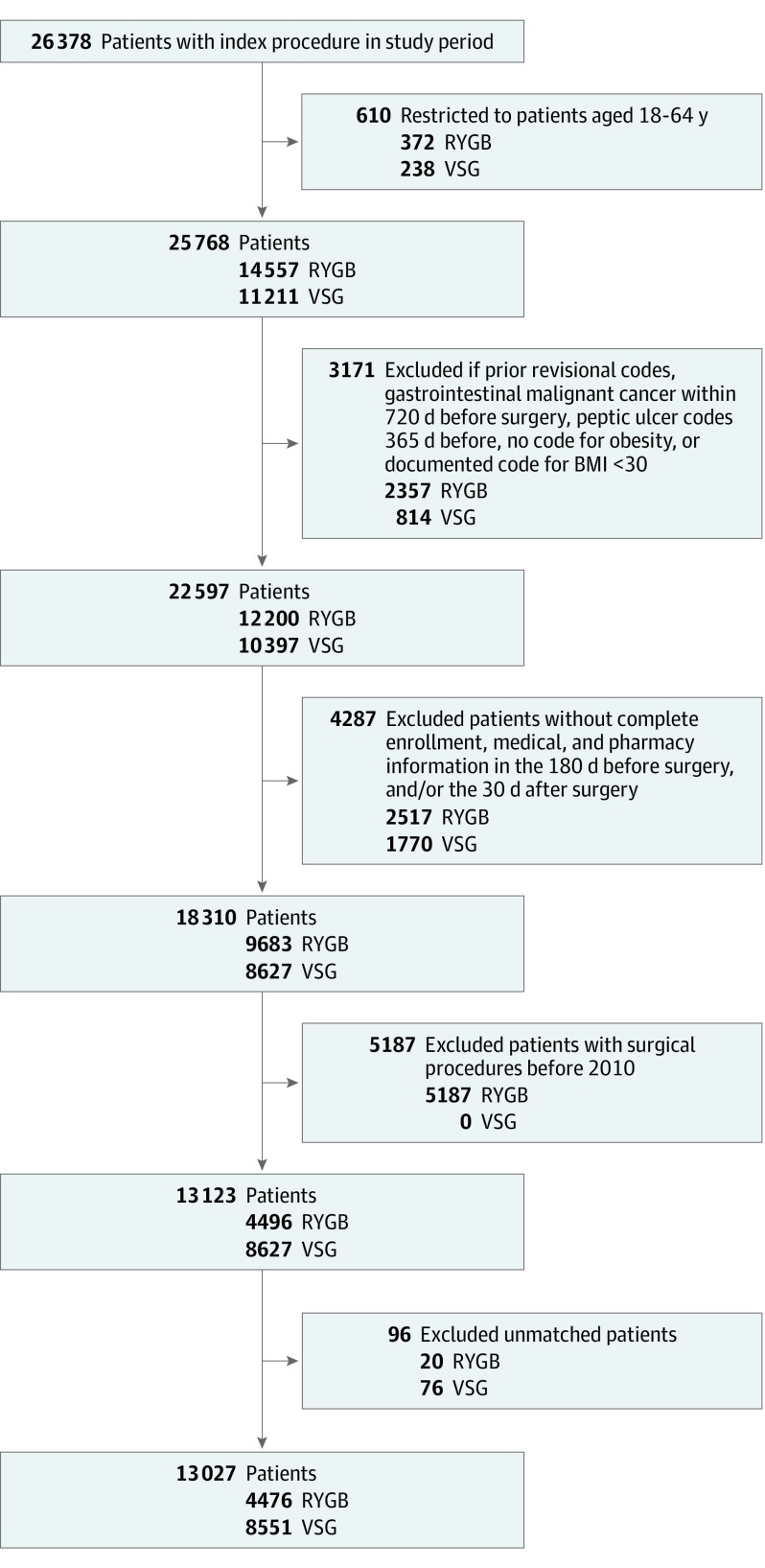
Flow Diagram for Cohort Selection Shown is the application of inclusion and exclusion criteria and resulting sample size of the Roux-en-Y gastric bypass (RYGB) group and vertical sleeve gastrectomy (VSG) group. Body Mass Index (BMI) indicates the most recently diagnosed BMI category (calculated as weight in kilograms divided by height in meters squared) coded before surgery.

Our prematch pool included 4496 patients undergoing RYGB and 8627 patients undergoing VSG, and the final weighted matched sample included 4476 patients undergoing RYGB and 8551 patients undergoing VSG (Figure 1). Among 13 027 patients, the mean (SD) age was 44.4 (10.3) years, 74.1% were female, and 49.9% resided in predominantly white neighborhoods (Table 1). Among the cohort, 13.7% had a preoperative BMI between 30 and 39.9, 45.8% had a preoperative BMI between 40 and 49.9, and 24.2% had a preoperative BMI of at least 50. At baseline, 47.3% had hypertension, 42.5% had type 2 diabetes, 38.5% had mental illness, and 59.5% had GERD. The RYGB group and VSG group were well matched with respect to all baseline characteristics and had similar follow-up time. Accounting for all potential censoring events, the median postoperative follow-up time was 1.6 years (interquartile range, 0.7-3.2 years), with 65.8% of patients undergoing RYGB or VSG still enrolled at 1 year after surgery, 41.9% still enrolled at 2 years after surgery, and 16.3% still enrolled at 4 years after surgery. However, when only excluding those truly lost to follow-up (ie, disenrolled), the percentage of eligible patients observed at 1, 2, and 4 years was 74.7%, 55.6%, and 31.4%, respectively (eTable 6 in the Supplement).

**Table 1.** Baseline Characteristics Before and After Matching Among Patients Undergoing RYGB vs VSG^a^

Variable	Before Matching, No. (%)		Standardized Difference	After Matching, No. (%)		Standardized Difference
	RYGB (n = 4496)	VSG (n = 8627)		RYGB (n = 4476)	VSG (n = 8551)	
Age group, y						
18-39	1461 (32.5)	3118 (36.1)	0.10	1453 (32.5)	2833 (33.1)	0.03
40-49	1497 (33.3)	2873 (33.3)		1492 (33.3)	2842 (33.2)	
50-59	1194 (26.6)	2074 (24.0)		1187 (26.5)	2260 (26.4)	
60-64	344 (7.7)	562 (6.5)		344 (7.7)	616 (7.2)	
Female sex	3394 (75.5)	6532 (75.7)	0.01	3378 (75.5)	6275 (73.4)	−0.05
Predominantly white neighborhood, ≥75%	2278 (50.7)	4135 (47.9)	0.08	2268 (50.7)	4236 (49.5)	0.06
Percentage of neighborhood residents without high school education^b^						
Less educated, ≥25%	453 (10.1)	795 (9.2)	0.05	450 (10.1)	785 (9.2)	0.03
More educated, <25%	3709 (82.5)	7085 (82.1)		3694 (82.5)	7188 (84.1)	
Missing	334 (7.4)	747 (8.7)		332 (7.4)	578 (6.8)	
Percentage of neighborhood residents below poverty line^c^						
Less poor, <10%	1933 (43.0)	3789 (43.9)	0.08	1924 (43.0)	3738 (43.7)	0.04
More poor, ≥10%	2228 (49.6)	4091 (47.4)		2219 (49.6)	4235 (49.5)	
Missing	335 (7.5)	747 (8.7)		333 (7.4)	578 (6.8)	
US region						
West	1128 (25.1)	1613 (18.7)	0.23	1128 (25.2)	2155 (25.2)	0.00
South	1876 (41.7)	4453 (51.6)		1876 (41.9)	3584 (41.9)	
Midwest	1028 (22.9)	1565 (18.1)		1022 (22.8)	1952 (22.8)	
Northeast	443 (9.9)	984 (11.4)		441 (9.9)	843 (9.9)	
Missing	21 (0.5)	12 (0.1)		9 (0.2)	17 (0.2)	
Year of surgery						
2010-2011	1493 (33.2)	898 (10.4)	0.63	1480 (33.1)	2827 (33.1)	0.00
2012-2013	1179 (26.2)	2057 (23.8)		1177 (26.3)	2249 (26.3)	
2014-2015	1041 (23.2)	3088 (35.8)		1038 (23.2)	1983 (23.2)	
2016-2017	783 (17.4)	2584 (30.0)		781 (17.4)	1492 (17.4)	
Follow-up duration, d						
<90	366 (8.1)	790 (9.2)	0.25	365 (8.2)	631 (7.4)	0.09
90-359	1262 (28.1)	2667 (30.9)		1256 (28.1)	2133 (24.9)	
360-719	1045 (23.2)	2319 (26.9)		1042 (23.3)	2041 (23.9)	
720-1079	645 (14.3)	1300 (15.1)		642 (14.3)	1293 (15.1)	
1080-1439	439 (9.8)	791 (9.2)		438 (9.8)	968 (11.3)	
≥1440	739 (16.4)	760 (8.8)		733 (16.4)	1485 (17.4)	
BMI category						
30-39.9	613 (13.6)	1508 (17.5)	0.28	612 (13.7)	1169 (13.7)	0.00
40-49.9	2051 (45.6)	4596 (53.3)		2047 (45.7)	3910 (45.7)	
50-59.9	889 (19.8)	1443 (16.7)		887 (19.8)	1695 (19.8)	
≥60	195 (4.3)	391 (4.5)		195 (4.4)	373 (4.4)	
Nonspecific obesity	703 (15.6)	649 (7.5)		699 (15.6)	1335 (15.6)	
Missing	45 (1.0)	40 (0.5)		36 (0.8)	69 (0.8)	
ACG comorbidity score ≥3	819 (18.2)	1406 (16.3)	−0.05	816 (18.2)	1434 (16.8)	−0.04
Hypertension	2153 (47.9)	3770 (43.7)	−0.08	2146 (47.9)	4010 (46.9)	−0.02
Type 2 diabetes	1909 (42.5)	2681 (31.1)	−0.24	1902 (42.5)	3634 (42.5)	0.00
GERD	2712 (60.3)	5216 (60.5)	0.00	2703 (60.4)	5046 (59.0)	−0.03
Cardiovascular disease	340 (7.6)	575 (6.7)	−0.03	339 (7.6)	651 (7.6)	0.00
Mental illness	1736 (38.6)	3567 (41.3)	0.06	1734 (38.7)	3278 (38.3)	−0.01
Liver disease	554 (12.3)	1179 (13.7)	0.04	550 (12.3)	1076 (12.6)	0.01
Kidney disease	112 (2.5)	162 (1.9)	−0.04	111 (2.5)	166 (1.9)	−0.04
Tobacco/smoking history	763 (17.0)	1498 (17.4)	0.01	762 (17.0)	1351 (15.8)	−0.03

Abbreviations: ACG, Adjusted Clinical Groups; BMI, body mass index (calculated as weight in kilograms divided by height in meters squared); GERD, gastroesophageal reflux disease; RYGB, Roux-en-Y gastric bypass; VSG, vertical sleeve gastrectomy.

^a^ We conducted coarsened exact matching on US region, year of surgery, BMI category, ACG comorbidity score group, and type 2 diabetes status. Standardized differences are the differences in means between the RYGB and VSG groups divided by the SD of the difference in means. Lower absolute values indicate greater similarity between RYGB and VSG, and values less than 0.2 indicate minimal differences between groups. Complete descriptions of baseline variable construction are given in the Covariates subsection of the present study’s Methods.

^b^ More educated neighborhoods were those where less than 25% of adult residents did not graduate from high school, and less educated neighborhoods were those where at least 25% of adult residents did not graduate from high school.

^c^ Neighborhoods with less poverty were those where less than 10% of households were below the poverty line, and neighborhoods with more poverty were those where at least 10% of households were below the poverty line.

### Primary Outcome of AOI

**Figure 2.**
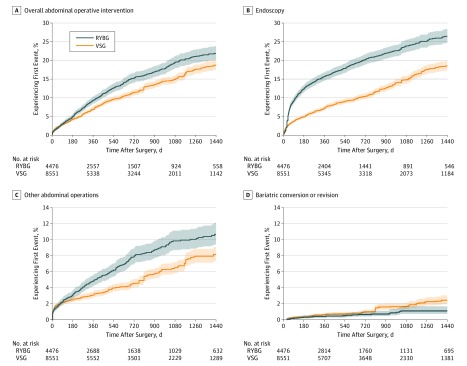
Time to Overall Abdominal Operative Intervention, Endoscopy, Other Abdominal Operation, or Bariatric Conversion or Revision in the Matched Roux-en-Y Gastric Bypass (RYGB) Group and Vertical Sleeve Gastrectomy (VSG) Group Numbers at risk are coarsened exact matching weighted and represent patients who remained enrolled and at risk (had not yet had an event of interest) at each time point. Shaded areas represent 95% CIs. Because many of the procedures took place in the later years of the data, some proportions of the RYGB and VSG groups lack complete follow-up not because of loss to follow-up (disenrollment) or events, but rather because of insufficient time between the date of surgery and the end of the data set. To more accurately represent completeness of follow-up accounting for this fact, eTable 6 in the Supplement lists counts and percentage enrolled relative to those truly eligible for complete follow-up at all relevant time points. A, Category includes any operative intervention on the abdomen (includes subcategories of biliary procedures, abdominal wall hernia repairs, conversions or revisions, and reoperation). Complete code list is in eTable 1 in the Supplement. B, Category includes any endoscopic procedure for diagnosis or treatment on the upper gastrointestinal tract. C, Category includes those abdominal operative procedures not captured under the categories of biliary, abdominal wall hernias, or conversion or revision and represents presumed complications. D, Category includes only subsequent bariatric procedures (eg, conversion from VSG to RYGB), as well as revisional procedures, such as gastrectomy.

During up to 4 years of follow-up, patients in the VSG group were less likely to undergo AOI than matched patients in the RYGB group (adjusted hazard ratio [aHR], 0.80; 95% CI, 0.72-0.89) (Table 2). The estimated cumulative incidence of AOI increased steadily during follow-up for both surgery types. For those in the RYGB and VSG groups, the respective percentages of patients undergoing AOI ranged from 2.9% (95% CI, 2.5%-3.5%) and 2.8% (95% CI, 2.4%-3.2%) at day 90 to 9.3% (95% CI, 8.5%-10.4%) and 6.9% (95% CI, 6.4%-7.6%) at 1 year for those in the RYGB and VSG groups, respectively. At year 4, the estimated cumulative incidence of AOI was 21.9% (95% CI, 20.1%-23.8%) among patients with RYGB and 18.7% (95% CI, 17.5%-20.1%) among patients with VSG (Table 2 and Figure 2).

**Table 2.** Results From Cox Proportional Hazards Regression Models Comparing Matched Cohorts of Patients Undergoing RYGB vs VSG, Up to 4 Years After Surgery, and Procedure-Specific Estimated Event Rates Based on Kaplan-Meier Plots^a^

Outcome Measure	Adjusted Model		Cumulative Incidence of Outcome, % (95% CI)^b^					
	Hazard Ratio (95% CI) for RYGB vs VSG	*P* Value	90 d After Index Procedure		1 y After Index Procedure		4 y After Index Procedure	
			RYGB	VSG	RYGB	VSG	RYGB	VSG
Remained enrolled, No./total No. (%)	NA	NA	4098/4384 (93.5)	7900/8382 (94.2)	2825/3941 (71.7)	5743/7538 (76.2)	701/2362 (29.7)	1420/4395 (32.3)
Overall abdominal operative intervention^c^	0.80 (0.72-0.89)	<.001	2.9 (2.5-3.5)	2.8 (2.4-3.2)	9.3 (8.5-10.4)	6.9 (6.4-7.6)	21.9 (20.1-23.8)	18.7 (17.5-20.1)
Biliary procedures^d^	0.77 (0.67-0.90)	.001	0.7 (0.5-1.0)	0.6 (0.5-0.8)	4.6 (4.0-5.4)	3.4 (3.0-3.9)	11.2 (9.9-12.7)	9.0 (8.1-10.0)
Abdominal wall hernia repair^e^	0.60 (0.47-0.75)	<.001	0.1 (0.0-0.2)	0.1 (0.0-0.2)	1.6 (1.3-2.2)	1.0 (0.8-1.3)	6.4 (5.3-7.7)	3.9 (3.3-4.6)
Bariatric conversion or revision^f^	1.83 (1.19-2.80)	.005	0.2 (0.1-0.3)	0.3 (0.2-0.4)	0.4 (0.2-0.6)	0.6 (0.5-0.8)	1.1 (0.7-1.7)	2.4 (1.9-3.0)
Other abdominal operations^g^	0.71 (0.61-0.82)	<.001	2.2 (1.8-2.7)	2.1 (1.8-2.5)	4.8 (4.2-5.5)	3.1 (2.7-3.5)	10.7 (9.4-12.1)	8.1 (7.3-9.1)
Endoscopy^h^	0.54 (0.49-0.59)	<.001	9.1 (8.3-10.0)	3.6 (3.3-4.1)	15.6 (14.5-16.8)	7.3 (6.7-7.9)	26.5 (24.6-28.4)	18.5 (17.3-19.9)
Enteral access^i^	0.58 (0.39-0.86)	.006	0.5 (0.4-0.8)	0.5 (0.4-0.7)	0.9 (0.7-1.3)	0.6 (0.5-0.8)	1.5 (1.1-2.1)	0.7 (0.5-0.9)
Other nonoperative interventions^j^	0.88 (0.62-1.26)	.48	0.5 (0.4-0.8)	0.5 (0.3-0.6)	0.9 (0.7-1.2)	0.6 (0.4-0.8)	1.7 (1.2-2.4)	1.7 (1.3-2.2)

Abbreviations: NA, not applicable; RYGB, Roux-en-Y gastric bypass; VSG, vertical sleeve gastrectomy.

^a^ Models were adjusted for all matched covariates, plus age group, sex, baseline adjusted clinical groups comorbidity score group, and presence of hypertension, gastroesophageal reflux disease, and mental illness.

^b^ From adjusted Kaplan-Meier plots at days 90, 360, and 1440 relative to index procedure.

^c^ Category includes any operative procedure on the abdomen (includes subcategories of biliary procedures, abdominal wall hernia repairs, conversions or revisions, and reoperation). Complete code list is in eTable 1 in the Supplement.

^d^ Category includes only procedures on the biliary tract, such as cholecystectomy and placement of drains in the biliary tree.

^e^ Category includes only repair of ventral hernias and other abdominal wall hernias and does not include internal hernias or paraesophageal hernias.

^f^ Category includes only subsequent bariatric procedures (eg, conversion from VSG to RYGB), as well as revisional procedures, such as gastrectomy.

^g^ Category includes those abdominal operative procedures not captured under the categories of biliary, abdominal wall hernias, or conversion or revision.

^h^ Category includes any endoscopic procedure for diagnosis or treatment on the upper gastrointestinal tract.

^i^ Category includes placement of gastrostomy tubes or other feeding devices, either percutaneously or through other means of access.

^j^ Category includes invasive but nonoperative procedures on the abdomen, such as paracentesis, or radiologically guided drainage procedures that do not involve incisions.

### Biliary Procedures, Abdominal Wall Hernia Repair, and Other Abdominal Operations

**Figure 3.**
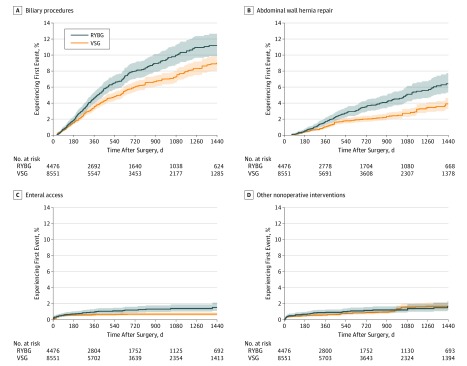
Time to First Biliary Procedure, Abdominal Wall Hernia Repair, Enteral Access, or Other Nonoperative Intervention in the Matched Roux-en-Y Gastric Bypass (RYGB) Group Members and Vertical Sleeve Gastrectomy (VSG) Group Members Numbers at risk are coarsened exact matching weighted and represent patients who remained enrolled and at risk (had not yet had an event of interest) at each time point. Shaded areas represent 95% CIs. Because many of the procedures took place in the later years of the data, some proportions of the RYGB and VSG groups lack complete follow-up not because of loss to follow-up (disenrollment) or events, but rather because of insufficient time between the date of their surgery and the end of our data set. To more accurately represent completeness of follow-up accounting for this fact, eTable 6 in the Supplement lists counts and percentage enrolled relative to those truly eligible for complete follow-up at all relevant time points. A, Category includes only procedures on the biliary tract, such as cholecystectomy and placement of drains in the biliary tree. B, Category includes only repair of ventral hernias and other abdominal wall hernias and does not include internal hernias or paraesophageal hernias. C, Category includes placement of gastrostomy tubes or other feeding devices, either percutaneously or through other means of access. D, Category includes invasive but nonoperative procedures on the abdomen, such as paracentesis, or radiologically guided drainage procedures that do not involve incisions.

The VSG group members were less likely than matched RYGB group members to undergo biliary procedures (aHR, 0.77; 95% CI, 0.67-0.90), abdominal wall hernia repair (aHR, 0.60; 95% CI, 0.47-0.75), and other abdominal operations (aHR, 0.71; 95% CI, 0.61-0.82) (Table 2 and Figure 3). Biliary procedures were one of the more common intervention subcategories after both bariatric procedure types, with an estimated 11.2% (95% CI, 9.9%-12.7%) of patients in the RYGB group and 9.0% (95% CI, 8.1%-10.0%) of patients in the VSG group undergoing a qualifying procedure by 4 years after surgery.

### Bariatric Conversion or Revision

In contrast to all other outcomes, bariatric conversion or revision was observed to be more likely among patients in the VSG group than in matched patients in the RYGB group (aHR, 1.83; 95% CI, 1.19-2.80). However, bariatric conversion or revision procedures generally occurred at a much lower frequency than other types of reinterventions. For example, by 1 year after the index date, an estimated 0.4% (95% CI, 0.2%-0.6%) of patients in the RYGB group and 0.6% (95% CI, 0.5%-0.8%) of those in the VSG group had undergone a qualifying procedure. By 4 years after the index date, the percentage had increased to 1.1% (95% CI, 0.7%-1.7%) of patients in the RYGB group and 2.4% (95% CI, 1.9%-3.0%) of those in the VSG group (Table 2 and Figure 2).

### Endoscopy and Enteral Access

Compared with the RYGB group, patients in the VSG group were less likely to undergo endoscopy (aHR, 0.54; 95% CI, 0.49-0.59) or require placement of enteral access (aHR, 0.58; 95% CI, 0.39-0.86). However, endoscopy was by far the most common intervention type for both groups of patients, with 26.5% (95% CI, 24.6%-28.4%) of those in the RYGB group and 18.5% (95% CI, 17.3%-19.9%) of those in the VSG group having an endoscopy performed by 4 years after the index procedure (Table 2, Figure 2, and Figure 3).

### Other Nonoperative Interventions

No difference in the risk of other nonoperative interventions was observed between patients in the RYGB group and those in the VSG group in the 4 years after an index procedure (aHR, 0.88; 95% CI, 0.62-1.26). Overall rates of these interventions were low, with just 1.7% (95% CI, 1.2%-2.4%) of patients in the RYGB group and 1.7% (95% CI, 1.3%-2.2%) of those in the VSG group coded as undergoing such a procedure (Table 2 and Figure 3).

### Sensitivity Analyses

Analyses using only *CPT* codes to define events and analyses on unmatched cohorts of patients resulted in findings similar to those of our main analysis (eTable 4, eTable 5, eTable 7, eFigure 1, eFigure 2, eFigure 3, and eFigure 4 in the Supplement). Within this sensitivity analysis (eTable 4, eFigure 1, and eFigure 2 in the Supplement), slightly lower rates of all outcome measures were observed for both procedures. For example, the *CPT*-based 4-year operative reintervention estimate for RYGB was 20.7% vs 21.9% when characterization included *ICD-9* and *ICD-10* codes. However, the magnitude and direction of between-procedure comparisons were unchanged.

## Discussion

Risk for subsequent intervention is an important consideration for patients and surgeons when selecting an initial bariatric procedure. In this nationwide cohort study, consistently higher risk of operative and nonoperative interventions was observed after RYGB compared with VSG, except for bariatric conversion or revision procedures, which were more likely among patients in the VSG group. However, absolute risk of bariatric conversion or revision was low in both groups, with cumulative incidence rates of 1.1% and 2.4% after 4 years among those in the RYGB and VSG groups, respectively. The geographic and sociodemographic diversity of the data, coupled with rigorous comparative effectiveness methods and the use of claims data to capture outcomes up to 4 years after an index procedure, strengthens these findings and adds considerably to the existing literature.

Previous studies’ estimates of the burden of operative intervention after bariatric surgery have varied, especially for time frames beyond 30 days. Estimated rates of abdominal reoperation or intervention after VSG have ranged from less than 1%^19,41^ to 16%^16,17^ at between 1 and 5 years. For RYGB, prior estimates have ranged from 6%^18^ to 22%^16,17^ over the same period. Variability between studies is likely because of different follow-up periods and outcome definitions, as well as variable completeness and accuracy of different data sources for outcome assessment.

Insurance claims are an ideal data source for identification of surgical procedures. These are high-cost events, for which claims are almost universally submitted, and they will be captured for enrolled members regardless of where a procedure takes place. In contrast, electronic health record–based studies may only capture follow-up procedures that take place in the health system at which an initial procedure was performed. Accordingly, we estimated that 21.9% of patients in the RYGB group and 18.7% of those in the VSG group had some form of AOI within 4 years after an index procedure, which are higher percentages than reported in most prior observational studies. These estimates also slightly exceeded 5-year rates reported by recent randomized clinical trials,^16,17^ possibly because data in the present study include patients with higher morbidity and lower adherence or surgeons with lower skill level (in aggregate) than those typically involved in clinical trials.

Few published studies have examined subtypes of operative intervention after bariatric surgery, but our large data set enabled us to look at several distinct, clinically important categories of reintervention. Biliary procedures were one of the most common operative subtypes, and estimates in the present study were similar to those published from a statewide registry in New York, where 9.1% of patients undergoing RYGB and 10.1% of patients undergoing VSG underwent cholecystectomy up to 5 years after an index procedure.^42^ The abdominal wall hernia repair findings merit discussion as well. Bariatric surgery can be used to promote weight loss before an elective ventral hernia repair^43^; therefore, some fraction of these procedures likely represents planned procedures, rather than complications of the bariatric surgery. The slightly higher rate of abdominal wall hernia repair among patients in the RYGB group, especially approximately 18 months after bariatric surgery (Figure 3), is of unclear clinical significance but could be driven by the overall higher rate of abdominal operations for patients in the RYGB group in the first postoperative year.

Bariatric conversion or revision was the only operative category that was more common among patients in the VSG group than in the RYGB group. One potential reason for this finding is that a subset of patients undergo VSG as part of a planned 2-stage procedure, a common pathway for those who are initially too heavy to undergo the more invasive RYGB.^1^ Alternatively, the higher rate of bariatric conversion or revisions among patients undergoing VSG could represent complications, such as worsening GERD symptoms, or could simply indicate that the procedure has a higher rate of weight regain than RYGB.^44,45^ The findings in this regard align with those of another study^21^ from the statewide registry in New York, which showed that bariatric conversion or revision was almost twice as common after VSG as it was after RYGB.

Our analyses suggest high rates of endoscopy after both RYGB and VSG: an estimated 26.5% and 18.5% of patients, respectively, had undergone at least 1 endoscopic procedure by 4 years after bariatric surgery. Although endoscopy is lower risk than most surgical procedures, it is nonetheless an invasive procedure, and patients should be made aware of its high likelihood after bariatric surgery. This is particularly salient because of the high prevalence of GERD among the bariatric patient population: the present study identified 59.5% of patients with GERD before surgery. Future analyses should assess the role that GERD plays in reintervention after bariatric surgery, especially since VSG is believed to exacerbate reflux more than RYGB.^1^

### Limitations

Limitations of our study include substantial loss to follow-up by year 4, which is typical of an administrative data set. It is possible that patients with longer follow-up are systematically different in ways that overrepresent or underrepresent the risk of subsequent operative interventions. However, we chose analytic methods that would maximize the validity of between-procedure comparisons despite loss to follow-up, including the use of Cox proportional hazards regression models and adjustment for changing population characteristics over time.

As with any observational study, it is possible that our findings are subject to unmeasured confounding. One important potential category of unmeasured confounders is clinician-level factors, such as surgeon case volumes. Prior studies indicate that for both RYGB^20,46^ and VSG^47^ having a surgeon with lower annual bariatric procedure volumes is associated with a higher risk of early reoperation and complication. Because of the nature of the data, we were also unable to examine how the volume of surgery performed at a particular center^10,48^ or the type of center (eg, outpatient surgical center or academic center) might have altered our outcomes. It is possible that there were unmeasured systematic differences for these clinician-level and facility-level factors between the RYGB group and the VSG group. It is also possible that our classification system for outcomes according to procedure codes led to some misclassification. For example, some procedures that we classified as other abdominal operations (eg, laparoscopic enterectomy) may have been viewed by the submitting surgeon as revisions. This phenomenon may have been more likely to affect subsequent operations after RYGB than after VSG, potentially leading to undercounting of bariatric conversion or revision for RYGB group members and slight overcounting of other abdominal operations. However, this should not alter the primary finding that the RYGB group overall required more subsequent AOIs than those in the matched VSG group. Also, because of the 30-day washout rule for certain interventions (eg, cholecystectomy and bariatric conversion or revision), early complications may have been undercounted. However, the 30-day estimated operation rates align well with the published literature.

## Conclusions

The findings of the present study add to the complex and emerging picture regarding procedure choice for patients considering bariatric surgery. Although RYGB appears to be slightly more effective than VSG for weight loss^16,17^ and type 2 diabetes remission,^23^ the current findings suggest it is also associated with a higher risk of subsequent operative and endoscopic interventions. It is crucial for clinicians to clearly communicate these potential risks to patients considering bariatric surgery as they weigh the potential benefits and complications of these two procedures.

## Notes

Supplement. **eTable 1.** CPT, ICD-9 Procedure, and ICD-10 Procedure Codes Used to Identify Reintervention Following Index Bariatric Procedures **eTable 2.** Procedures Subject to a 30-Day Washout Period Following the Date of the Index Bariatric Procedure **eTable 3.** Procedure and Pharmacy Codes Used to Define Study Cohort and Baseline Comorbidities **eTable 4.** Sensitivity Results From Cox Proportional Hazards Models Comparing Matched Cohorts of VSG and RYGB Patients, Up to 48 Months After Surgery, and Procedure-Specific Estimated Event Rates Based on Kaplan-Meier Plots–Using CPT Codes Only to Define Events **eTable 5.** Results From Cox Proportional Hazards Models Comparing Unmatched Cohorts of VSG and RYGB Patients, Up to 48 Months After Surgery, and Procedure-Specific Estimated Event Rates Based on Kaplan-Meier Plots **eTable 6.** Number (%) of Patients Remaining Enrolled Over Postoperative Follow-up Period, by Surgery Type **eTable 7.** Number (%) of Patients Remaining Enrolled in Unmatched Cohort, Over Postoperative Period, by Surgery Type **eFigure 1.** Time to Operative Abdominal Intervention, Endoscopy, Other Abdominal Operation, or Bariatric Conversion or Revision in Matched Cohorts of RYGB and VSG Patients, Using Only CPT Codes to Define Event **eFigure 2.** Time to First Biliary Procedure, Abdominal Wall Hernia Repair, Enteral Access, or Other Nonoperative Intervention in Matched Cohorts of RYGB and VSG Patients, Using Only CPT Codes to Define Event **eFigure 3.** Time to First Operative Abdominal Intervention, Endoscopy, Other Abdominal Operation, or Bariatric Conversion or Revision in Unmatched Cohorts of RYGB and VSG Patients **eFigure 4.** Time to First Biliary Procedure, Abdominal Wall Hernia Repair, Enteral Access, or Other Nonoperative Intervention in Unmatched Cohorts of RYGB and VSG Patients
