# Supplementary material for: Risk of Operative and Nonoperative Interventions Up to 4 Years After Roux-en-Y Gastric Bypass vs Vertical Sleeve Gastrectomy in a Nationwide US Commercial Insurance Claims Database
Source: JAMA Netw Open. 2019 Dec 18;2(12):e1917603. doi: 10.1001/jamanetworkopen.2019.17603 (PMC6991222; doi:10.1001/jamanetworkopen.2019.17603)
Supplement: Supplement. — eTable 1. CPT, ICD-9 Procedure, and ICD-10 Procedure Codes Used to Identify Reintervention Following Index Bariatric Procedures eTable 2. Procedures Subject to a 30-Day Washout Period Following the Date of the Index Bariatric Procedure eTable 3. Procedure and Pharmacy Codes Used to Define Study Cohort and Baseline Comorbidities eTable 4. Sensitivity Results From Cox Proportional Hazards Models Comparing Matched Cohorts of VSG and RYGB Patients, Up to 48 Months After Surgery, and Procedure-Specific Estimated Event Rates Based on Kaplan-Meier Plots–Using CPT Codes Only to Define Events eTable 5. Results From Cox Proportional Hazards Models Comparing Unmatched Cohorts of VSG and RYGB Patients, Up to 48 Months After Surgery, and Procedure-Specific Estimated Event Rates Based on Kaplan-Meier Plots eTable 6. Number (%) of Patients Remaining Enrolled Over Postoperative Follow-up Period, by Surgery Type eTable 7. Number (%) of Patients Remaining Enrolled in Unmatched Cohort, Over Postoperative Period, by Surgery Type eFigure 1. Time to Operative Abdominal Intervention, Endoscopy, Other Abdominal Operation, or Bariatric Conversion or Revision in Matched Cohorts of RYGB and VSG Patients, Using Only CPT Codes to Define Event eFigure 2. Time to First Biliary Procedure, Abdominal Wall Hernia Repair, Enteral Access, or Other Nonoperative Intervention in Matched Cohorts of RYGB and VSG Patients, Using Only CPT Codes to Define Event eFigure 3. Time to First Operative Abdominal Intervention, Endoscopy, Other Abdominal Operation, or Bariatric Conversion or Revision in Unmatched Cohorts of RYGB and VSG Patients eFigure 4. Time to First Biliary Procedure, Abdominal Wall Hernia Repair, Enteral Access, or Other Nonoperative Intervention in Unmatched Cohorts of RYGB and VSG Patients [file jamanetwopen-2-e1917603-s001.pdf]

# Supplementary Online Content

Lewis KH, Arterburn DE, Callaway K, et al. Risk of operative and nonoperative interventions up to 4 years after Roux-en-Y gastric bypass vs vertical sleeve gastrectomy in a nationwide US commercial insurance claims database. *JAMA Netw Open*. 2019;2(12):e1917603. doi:10.1001/jamanetworkopen.2019.17603

**eTable 1.** CPT, ICD-9 Procedure, and ICD-10 Procedure Codes Used to Identify Reintervention Following Index Bariatric Procedures

**eTable 2.** Procedures Subject to a 30-Day Washout Period Following the Date of the Index Bariatric Procedure

**eTable 3.** Procedure and Pharmacy Codes Used to Define Study Cohort and Baseline Comorbidities

**eTable 4.** Sensitivity Results From Cox Proportional Hazards Models Comparing Matched Cohorts of VSG and RYGB Patients, Up to 48 Months After Surgery, and Procedure-Specific Estimated Event Rates Based on Kaplan-Meier Plots--Using CPT Codes Only to Define Events

**eTable 5.** Results From Cox Proportional Hazards Models Comparing Unmatched Cohorts of VSG and RYGB Patients, Up to 48 Months After Surgery, and Procedure-Specific Estimated Event Rates Based on Kaplan-Meier Plots

**eTable 6.** Number (%) of Patients Remaining Enrolled Over Postoperative Follow-up Period, by Surgery Type

**eTable 7.** Number (%) of Patients Remaining Enrolled in Unmatched Cohort, Over Postoperative Period, by Surgery Type

**eFigure 1.** Time to Operative Abdominal Intervention, Endoscopy, Other Abdominal Operation, or Bariatric Conversion or Revision in Matched Cohorts of RYGB and VSG Patients, Using Only CPT Codes to Define Event

**eFigure 2.** Time to First Biliary Procedure, Abdominal Wall Hernia Repair, Enteral Access, or Other Nonoperative Intervention in Matched Cohorts of RYGB and VSG Patients, Using Only CPT Codes to Define Event

**eFigure 3.** Time to First Operative Abdominal Intervention, Endoscopy, Other Abdominal Operation, or Bariatric Conversion or Revision in Unmatched Cohorts of RYGB and VSG Patients

**eFigure 4.** Time to First Biliary Procedure, Abdominal Wall Hernia Repair, Enteral Access, or Other Nonoperative Intervention in Unmatched Cohorts of RYGB and VSG Patients

This supplementary material has been provided by the authors to give readers additional information about their work.

© 2019 Lewis KH et al. *JAMA Network Open*.

**eTable 1.** CPT, ICD-9 Procedure, and ICD-10 Procedure Codes
Used to Identify Reintervention Following Index Bariatric Procedures

| Category | Code Type | Code | Code Description |
|---|---|---|---|
| BILIARY PROCEDURE | CPT | 47490 | Incision of gallbladder |
| | | 47510 | Insert catheter, bile duct |
| | | 47511 | Insert bile duct drain |
| | | 47534 | Placement of biliary drainage catheter |
| | | 47542 | Dilate biliary duct/ampulla |
| | | 47562 | Laparoscopic cholecystectomy |
| | | 47563 | Laparoscopy, surgical; cholecystectomy with cholangiography |
| | | 47564 | Laparoscopy, surgical; cholecystectomy with exploration of common duct |
| | | 47600 | Cholecystectomy |
| | | 47605 | Cholecystectomy; with cholangiography |
| | | 47610 | Cholecystectomy with exploration of common duct |
| | | 47999 | Bile tract surgery procedure |
| BILIARY PROCEDURE | ICD-9 procedure | 51.0 | Cholecystectomy |
| | | 51.03 | Other cholecystostomy |
| | | 51.04 | Other cholecystostomy |
| | | 51.23 | Laparoscopic cholecystectomy |
| | | 51.92 | Closure of cholecystostomy |
| | | 51.93 | Closure of other biliary fistula |
| | | 51.94 | Revision anastomosis biliary tract |
| | | 51.99 | Other operations on biliary tract |
| BILIARY PROCEDURE | ICD-10 procedure | 0F190Z3 | Bypass Common Bile Duct to Duodenum, Open Approach |
| | | 0F754DZ | Dilation of Right Hepatic Duct with Intraluminal Device, Percutaneous Endoscopic Approach |
| | | 0F764DZ | Dilation of Left Hepatic Duct with Intraluminal Device, Percutaneous Endoscopic Approach |
| | | 0F774DZ | Dilation of Common Hepatic Duct with Intraluminal Device, Percutaneous Endoscopic Approach |
| | | 0F784DZ | Dilation of Cystic Duct with Intraluminal Device, Percutaneous Endoscopic Approach |
| | | 0F794DZ | Dilation of Common Bile Duct with Intraluminal Device, Percutaneous Endoscopic Approach |
| | | 0F9400Z | Drainage of Gallbladder with Drainage Device, Open Approach |
| | | 0F940ZZ | Drainage of Gallbladder, Open Approach |
| | | 0F9C40Z | Drainage of Ampulla of Vater with Drainage Device, Percutaneous Endoscopic Approach |
| | | 0F9C4ZZ | Drainage of Ampulla of Vater, Percutaneous Endoscopic Approach |
| | | 0FC40ZZ | Extirpation of Matter from Gallbladder, Open Approach |
| | | 0FC43ZZ | Extirpation of Matter from Gallbladder, Percutaneous Approach |
| | | 0FC44ZZ | Extirpation of Matter from Gallbladder, Percutaneous Endoscopic Approach |
| | | 0FC48ZZ | Extirpation of Matter from Gallbladder, Via Natural or Artificial Opening Endoscopic |
| | | 0FC54ZZ | Extirpation of Matter from Right Hepatic Duct, Percutaneous Endoscopic Approach |
| | | 0FC64ZZ | Extirpation of Matter from Left Hepatic Duct, Percutaneous Endoscopic Approach |
| | | 0FC74ZZ | Extirpation of Matter from Common Hepatic Duct, Percutaneous Endoscopic Approach |
| | | 0FC84ZZ | Extirpation of Matter from Cystic Duct, Percutaneous Endoscopic Approach |
| | | 0FC94ZZ | Extirpation of Matter from Common Bile Duct, Percutaneous Endoscopic Approach |
| | | 0FCC4ZZ | Extirpation of Matter from Ampulla of Vater, Percutaneous Endoscopic Approach |
| | | 0FF40ZZ | Fragmentation in Gallbladder, Open Approach |
| | | 0FF43ZZ | Fragmentation in Gallbladder, Percutaneous Approach |
| | | 0FF44ZZ | Fragmentation in Gallbladder, Percutaneous Endoscopic Approach |
| | | 0FF47ZZ | Fragmentation in Gallbladder, Via Natural or Artificial Opening |
| | | 0FF48ZZ | Fragmentation in Gallbladder, Via Natural or Artificial Opening Endoscopic |
| | | 0FH402Z | Insertion of Monitoring Device into Gallbladder, Open Approach |
| | | 0FH40YZ | Insertion of Other Device into Gallbladder, Open Approach |
| | | 0FH432Z | Insertion of Monitoring Device into Gallbladder, Percutaneous Approach |
| | | 0FH43YZ | Insertion of Other Device into Gallbladder, Percutaneous Approach |
| | | 0FH442Z | Insertion of Monitoring Device into Gallbladder, Percutaneous Endoscopic Approach |
| | | 0FH44YZ | Insertion of Other Device into Gallbladder, Percutaneous Endoscopic Approach |
| | | 0FHB4DZ | Insertion of Intraluminal Device into Hepatobiliary Duct, Percutaneous Endoscopic Approach |
| | | 0FJ44ZZ | Inspection of Gallbladder, Percutaneous Endoscopic Approach |
| | | 0FJB4ZZ | Inspection of Hepatobiliary Duct, Percutaneous Endoscopic Approach |
| | | 0FL50CZ | Occlusion of Right Hepatic Duct with Extraluminal Device, Open Approach |
| | | 0FL50DZ | Occlusion of Right Hepatic Duct with Intraluminal Device, Open Approach |
| | | 0FL50ZZ | Occlusion of Right Hepatic Duct, Open Approach |
| | | 0FL60CZ | Occlusion of Left Hepatic Duct with Extraluminal Device, Open Approach |
| | | 0FL60DZ | Occlusion of Left Hepatic Duct with Intraluminal Device, Open Approach |
| | | 0FL60ZZ | Occlusion of Left Hepatic Duct, Open Approach |
| | | 0FL70CZ | Occlusion of Common Hepatic Duct with Extraluminal Device, Open Approach |
| | | 0FL70DZ | Occlusion of Common Hepatic Duct with Intraluminal Device, Open Approach |
| | | 0FL70ZZ | Occlusion of Common Hepatic Duct, Open Approach |
| | | 0FL80CZ | Occlusion of Cystic Duct with Extraluminal Device, Open Approach |
| | | 0FL80DZ | Occlusion of Cystic Duct with Intraluminal Device, Open Approach |
| | | 0FL80ZZ | Occlusion of Cystic Duct, Open Approach |
| | | 0FL90CZ | Occlusion of Common Bile Duct with Extraluminal Device, Open Approach |
| | | 0FL90DZ | Occlusion of Common Bile Duct with Intraluminal Device, Open Approach |
| | | 0FL90ZZ | Occlusion of Common Bile Duct, Open Approach |
| | | 0FM40ZZ | Reattachment of Gallbladder, Open Approach |
| | | 0FN40ZZ | Release Gallbladder, Open Approach |
| | | 0FN43ZZ | Release Gallbladder, Percutaneous Approach |
| | | 0FN44ZZ | Release Gallbladder, Percutaneous Endoscopic Approach |

© 2019 Lewis KH et al. *JAMA Network Open*.

| | | | |
|---|---|---|---|
| BILIARY PROCEDURE | ICD-10 procedure | 0FN50ZZ | Release Right Hepatic Duct, Open Approach |
| | | 0FN53ZZ | Release Right Hepatic Duct, Percutaneous Approach |
| | | 0FN54ZZ | Release Right Hepatic Duct, Percutaneous Endoscopic Approach |
| | | 0FN57ZZ | Release Right Hepatic Duct, Via Natural or Artificial Opening |
| | | 0FN60ZZ | Release Left Hepatic Duct, Open Approach |
| | | 0FN63ZZ | Release Left Hepatic Duct, Percutaneous Approach |
| | | 0FN64ZZ | Release Left Hepatic Duct, Percutaneous Endoscopic Approach |
| | | 0FN67ZZ | Release Left Hepatic Duct, Via Natural or Artificial Opening |
| | | 0FN70ZZ | Release Common Hepatic Duct, Open Approach |
| | | 0FN73ZZ | Release Common Hepatic Duct, Percutaneous Approach |
| | | 0FN74ZZ | Release Common Hepatic Duct, Percutaneous Endoscopic Approach |
| | | 0FN77ZZ | Release Common Hepatic Duct, Via Natural or Artificial Opening |
| | | 0FN80ZZ | Release Cystic Duct, Open Approach |
| | | 0FN83ZZ | Release Cystic Duct, Percutaneous Approach |
| | | 0FN84ZZ | Release Cystic Duct, Percutaneous Endoscopic Approach |
| | | 0FN87ZZ | Release Cystic Duct, Via Natural or Artificial Opening |
| | | 0FN90ZZ | Release Common Bile Duct, Open Approach |
| | | 0FN93ZZ | Release Common Bile Duct, Percutaneous Approach |
| | | 0FN94ZZ | Release Common Bile Duct, Percutaneous Endoscopic Approach |
| | | 0FN97ZZ | Release Common Bile Duct, Via Natural or Artificial Opening |
| | | 0FP400Z | Removal of Drainage Device from Gallbladder, Open Approach |
| | | 0FP402Z | Removal of Monitoring Device from Gallbladder, Open Approach |
| | | 0FP40YZ | Removal of Other Device from Gallbladder, Open Approach |
| | | 0FP430Z | Removal of Drainage Device from Gallbladder, Percutaneous Approach |
| | | 0FP432Z | Removal of Monitoring Device from Gallbladder, Percutaneous Approach |
| | | 0FP43YZ | Removal of Other Device from Gallbladder, Percutaneous Approach |
| | | 0FP440Z | Removal of Drainage Device from Gallbladder, Percutaneous Endoscopic Approach |
| | | 0FP442Z | Removal of Monitoring Device from Gallbladder, Percutaneous Endoscopic Approach |
| | | 0FP44YZ | Removal of Other Device from Gallbladder, Percutaneous Endoscopic Approach |
| | | 0FQ50ZZ | Repair Right Hepatic Duct, Open Approach |
| | | 0FQ53ZZ | Repair Right Hepatic Duct, Percutaneous Approach |
| | | 0FQ54ZZ | Repair Right Hepatic Duct, Percutaneous Endoscopic Approach |
| | | 0FQ57ZZ | Repair Right Hepatic Duct, Via Natural or Artificial Opening |
| | | 0FQ58ZZ | Repair Right Hepatic Duct, Via Natural or Artificial Opening Endoscopic |
| | | 0FQ60ZZ | Repair Left Hepatic Duct, Open Approach |
| | | 0FQ63ZZ | Repair Left Hepatic Duct, Percutaneous Approach |
| | | 0FQ64ZZ | Repair Left Hepatic Duct, Percutaneous Endoscopic Approach |
| | | 0FQ67ZZ | Repair Left Hepatic Duct, Via Natural or Artificial Opening |
| | | 0FQ68ZZ | Repair Left Hepatic Duct, Via Natural or Artificial Opening Endoscopic |
| | | 0FQ70ZZ | Repair Common Hepatic Duct, Open Approach |
| | | 0FQ73ZZ | Repair Common Hepatic Duct, Percutaneous Approach |
| | | 0FQ74ZZ | Repair Common Hepatic Duct, Percutaneous Endoscopic Approach |
| | | 0FQ77ZZ | Repair Common Hepatic Duct, Via Natural or Artificial Opening |
| | | 0FQ78ZZ | Repair Common Hepatic Duct, Via Natural or Artificial Opening Endoscopic |
| | | 0FQ80ZZ | Repair Cystic Duct, Open Approach |
| | | 0FQ83ZZ | Repair Cystic Duct, Percutaneous Approach |
| | | 0FQ84ZZ | Repair Cystic Duct, Percutaneous Endoscopic Approach |
| | | 0FQ87ZZ | Repair Cystic Duct, Via Natural or Artificial Opening |
| | | 0FQ88ZZ | Repair Cystic Duct, Via Natural or Artificial Opening Endoscopic |
| | | 0FQ90ZZ | Repair Common Bile Duct, Open Approach |
| | | 0FQ93ZZ | Repair Common Bile Duct, Percutaneous Approach |
| | | 0FQ94ZZ | Repair Common Bile Duct, Percutaneous Endoscopic Approach |
| | | 0FQ97ZZ | Repair Common Bile Duct, Via Natural or Artificial Opening |
| | | 0FQ98ZZ | Repair Common Bile Duct, Via Natural or Artificial Opening Endoscopic |
| | | 0FR50JZ | Replacement of Right Hepatic Duct with Synthetic Substitute, Open Approach |
| | | 0FR54JZ | Replacement of Right Hepatic Duct with Synthetic Substitute, Percutaneous Endoscopic Approach |
| | | 0FR58JZ | Replacement of Right Hepatic Duct with Synthetic Substitute, Via Natural or Artificial Opening Endoscopic |
| | | 0FR60JZ | Replacement of Left Hepatic Duct with Synthetic Substitute, Open Approach |
| | | 0FR64JZ | Replacement of Left Hepatic Duct with Synthetic Substitute, Percutaneous Endoscopic Approach |
| | | 0FR68JZ | Replacement of Left Hepatic Duct with Synthetic Substitute, Via Natural or Artificial Opening Endoscopic |
| | | 0FR70JZ | Replacement of Common Hepatic Duct with Synthetic Substitute, Open Approach |
| | | 0FR74JZ | Replacement of Common Hepatic Duct with Synthetic Substitute, Percutaneous Endoscopic Approach |
| | | 0FR78JZ | Replacement of Common Hepatic Duct with Synthetic Substitute, Via Natural or Artificial Opening Endoscopic |
| | | 0FR80JZ | Replacement of Cystic Duct with Synthetic Substitute, Open Approach |
| | | 0FR84JZ | Replacement of Cystic Duct with Synthetic Substitute, Percutaneous Endoscopic Approach |
| | | 0FR88JZ | Replacement of Cystic Duct with Synthetic Substitute, Via Natural or Artificial Opening Endoscopic |
| | | 0FR90JZ | Replacement of Common Bile Duct with Synthetic Substitute, Open Approach |
| | | 0FR94JZ | Replacement of Common Bile Duct with Synthetic Substitute, Percutaneous Endoscopic Approach |
| | | 0FR98JZ | Replacement of Common Bile Duct with Synthetic Substitute, Via Natural or Artificial Opening Endoscopic |
| | | 0FS40ZZ | Reposition Gallbladder, Open Approach |
| | | 0FS44ZZ | Reposition Gallbladder, Percutaneous Endoscopic Approach |
| | | 0FT44ZZ | Resection of Gallbladder, Percutaneous Endoscopic Approach |
| | | 0FV50CZ | Restriction of Right Hepatic Duct with Extraluminal Device, Open Approach |
| | | 0FV50DZ | Restriction of Right Hepatic Duct with Intraluminal Device, Open Approach |
| | | 0FV50ZZ | Restriction of Right Hepatic Duct, Open Approach |
| | | 0FV60CZ | Restriction of Left Hepatic Duct with Extraluminal Device, Open Approach |
| | | 0FV60DZ | Restriction of Left Hepatic Duct with Intraluminal Device, Open Approach |
| | | 0FV60ZZ | Restriction of Left Hepatic Duct, Open Approach |
| | | 0FV70CZ | Restriction of Common Hepatic Duct with Extraluminal Device, Open Approach |
| | | 0FV70DZ | Restriction of Common Hepatic Duct with Intraluminal Device, Open Approach |

© 2019 Lewis KH et al. *JAMA Network Open*.

| | | | |
|---|---|---|---|
| BILIARY PROCEDURE | ICD-10 procedure | 0FV70ZZ | Restriction of Common Hepatic Duct, Open Approach |
| | | 0FV80CZ | Restriction of Cystic Duct with Extraluminal Device, Open Approach |
| | | 0FV80DZ | Restriction of Cystic Duct with Intraluminal Device, Open Approach |
| | | 0FV80ZZ | Restriction of Cystic Duct, Open Approach |
| | | 0FV90CZ | Restriction of Common Bile Duct with Extraluminal Device, Open Approach |
| | | 0FV90DZ | Restriction of Common Bile Duct with Intraluminal Device, Open Approach |
| | | 0FV90ZZ | Restriction of Common Bile Duct, Open Approach |
| | | 0FW400Z | Revision of Drainage Device in Gallbladder, Open Approach |
| | | 0FW402Z | Revision of Monitoring Device in Gallbladder, Open Approach |
| | | 0FW40DZ | Revision of Intraluminal Device in Gallbladder, Open Approach |
| | | 0FW40YZ | Revision of Other Device in Gallbladder, Open Approach |
| | | 0FW430Z | Revision of Drainage Device in Gallbladder, Percutaneous Approach |
| | | 0FW432Z | Revision of Monitoring Device in Gallbladder, Percutaneous Approach |
| | | 0FW43DZ | Revision of Intraluminal Device in Gallbladder, Percutaneous Approach |
| | | 0FW43YZ | Revision of Other Device in Gallbladder, Percutaneous Approach |
| | | 0FW440Z | Revision of Drainage Device in Gallbladder, Percutaneous Endoscopic Approach |
| | | 0FW442Z | Revision of Monitoring Device in Gallbladder, Percutaneous Endoscopic Approach |
| | | 0FW44DZ | Revision of Intraluminal Device in Gallbladder, Percutaneous Endoscopic Approach |
| | | 0FW44YZ | Revision of Other Device in Gallbladder, Percutaneous Endoscopic Approach |
| ENDOSCOPY | CPT | 43200 | Esophagoscopy w/ brushing or washing |
| | | 43201 | Esophagoscopy w/ directed submucosal injection(s) |
| | | 43202 | Esophagoscopy w/bx |
| | | 43204 | Esophagoscopy flex transoral injection varices |
| | | 43205 | Esophagoscopy with band ligation varices |
| | | 43210 | Esophagoscopy with fundoplasty |
| | | 43212 | Esophagoscopy with stent placement |
| | | 43213 | Esophagoscopy with dilation |
| | | 43214 | Esophagoscopy with dilation |
| | | 43215 | Esophagoscopy remove foreign body |
| | | 43216 | Esophagoscopy w/removal of tumor(s) using bipolar cautery |
| | | 43217 | Esophagoscopy w/removal of tumor(s) using snare |
| | | 43219 | Esophagoscopy w/ stent or tube placement |
| | | 43220 | Esophagoscopy balloon <30mm |
| | | 43226 | Esophagoscopy with dilation |
| | | 43227 | Esophagoscopy w/ control of bleeding |
| | | 43228 | Esophagoscopy w/control of bleeding |
| | | 43232 | Esophagoscopy w/tumor ablation |
| | | 43233 | Esophagoscopy w/intramural or transmural aspiration/bx |
| | | 43234 | EGD balloon dil esoph30 mm/> |
| | | 43235 | Upper gi endoscopy |
| | | 43236 | Endo diagnostic brush wash |
| | | 43237 | EGD w/directed submucosal injection(s) |
| | | 43239 | EGD with us |
| | | 43240 | EGD w/bx |
| | | 43241 | EGD with transmural drn pseudocyst |
| | | 43242 | EGD w/insertion transluminal tube |
| | | 43243 | EGD w/us guided bx |
| | | 43244 | EGD w/injection varices |
| | | 43245 | EGD w band lign varices |
| | | 43247 | EGD dilate stricture |
| | | 43248 | EGD remove foreign body |
| | | 43249 | EGD w/dilation of esophagus |
| | | 43250 | Esoph EGD dilation <30mm |
| | | 43251 | Endoscopy hot bx forceps |
| | | 43253 | EGD w/removal of tumor(s) using snare |
| | | 43254 | Operative upper gi endoscopy |
| | | 43255 | Operative upper gi endoscopy |
| | | 43256 | EGD control bleeding any |
| | | 43257 | Upper endoscopy with stent |
| | | 43258 | EGD w/thermal energy for gerd treatment |
| | | 43259 | EGD w/tumor ablation |
| | | 43260 | EGD with eus to duod |
| | | 43264 | ERCP, with removal of calculi/debris from biliary/pancreatic duct(s) |
| | | 43265 | ERCP, with destruction of calculi |
| | | 43266 | EGD , with placement of endoscopic stent |
| | | 43270 | EGD , with ablation of tumors |
| | | 43274 | ERCP with placement of endoscopic stent into biliary or pancreatic duct |
| | | 43275 | ERCP with removal of foreign body(s) or stent(s) from biliary/pancreatic duct(s) |
| | | 43277 | ERCP with trans-endoscopic balloon dilation of biliary/pancreatic duct(s) or of ampulla (sphincteroplasty) |
| | | 43450 | Dilation esophagus using sound |
| | | 43453 | Dilation esophagus guide wire |
| | | 43456 | Dilation esophagus balloon |
| | | 43458 | Dilation esophagus >30mm for achalasia |
| | | 43460 | Esophagogastric tamponade, with balloon |
| | | 44360 | Small bowel endoscopy |
| | | 44361 | Small bowel endoscopy w/bx |
| | | 44372 | Small bowel endoscopy |
| | | 44373 | Small bowel endoscopy |
| | | 44376 | Small bowel endoscopy w/ or w/o brushing or washing |

© 2019 Lewis KH et al. *JAMA Network Open*.

| | | | |
|---|---|---|---|
| ENDOSCOPY | ICD-9 Procedure | 42.21 | Esophagoscopy by incis |
| | | 42.22 | Esophagoscopy through stoma |
| | | 42.23 | Esophagoscopy NEC |
| | | 42.24 | Closed bx of esophagus |
| | | 42.25 | Open biopsy of esophagus |
| | | 42.29 | Esophageal dx proc not elsewhere classified |
| | | 42.92 | Dilation of esophagus |
| | | 43.41 | Endo exc/destruc les/tissue stomach |
| | | 44.13 | Other gastroscopy |
| | | 44.22 | Endoscopic dilation of pylorus |
| | | 44.43 | Endo control of gastric/duoden bleed |
| | | 45.11 | Eransabd endoscopy sm intestine |
| | | 45.12 | Endosc sm bowel through stoma |
| | | 45.13 | Other endoscopy small intestine |
| | | 45.14 | Closed small bowel bx |
| | | 51.85 | Endo sphincterotomy & papillotomy |
| | | 51.87 | Endoscopic insertion stent into bd |
| | | 51.88 | Endo removal stone from bili tract |
| ENDOSCOPY | ICD-10 Procedure | 0D568ZZ | Destruction of Stomach, Via Natural or Artificial Opening Endoscopic |
| | | 0D578ZZ | Destruction of Stomach, Pylorus, Via Natural or Artificial Opening Endoscopic |
| | | 0D717DZ | Dilation of Upper Esophagus with Intraluminal Device, Via Natural or Artificial Opening |
| | | 0D717ZZ | Dilation of Upper Esophagus, Via Natural or Artificial Opening |
| | | 0D718DZ | Dilation of Upper Esophagus with Intraluminal Device, Via Natural or Artificial Opening Endoscopic |
| | | 0D718ZZ | Dilation of Upper Esophagus, Via Natural or Artificial Opening Endoscopic |
| | | 0D727DZ | Dilation of Middle Esophagus with Intraluminal Device, Via Natural or Artificial Opening |
| | | 0D727ZZ | Dilation of Middle Esophagus, Via Natural or Artificial Opening |
| | | 0D728DZ | Dilation of Middle Esophagus with Intraluminal Device, Via Natural or Artificial Opening Endoscopic |
| | | 0D728ZZ | Dilation of Middle Esophagus, Via Natural or Artificial Opening Endoscopic |
| | | 0D737DZ | Dilation of Lower Esophagus with Intraluminal Device, Via Natural or Artificial Opening |
| | | 0D737ZZ | Dilation of Lower Esophagus, Via Natural or Artificial Opening |
| | | 0D738DZ | Dilation of Lower Esophagus with Intraluminal Device, Via Natural or Artificial Opening Endoscopic |
| | | 0D738ZZ | Dilation of Lower Esophagus, Via Natural or Artificial Opening Endoscopic |
| | | 0D747DZ | Dilation of Esophagogastric Junction with Intraluminal Device, Via Natural or Artificial Opening |
| | | 0D747ZZ | Dilation of Esophagogastric Junction, Via Natural or Artificial Opening |
| | | 0D748DZ | Dilation esophagogast jun il n/a en |
| | | 0D748ZZ | Dilation of Esophagogastric Junction, Via Natural or Artificial Opening Endoscopic |
| | | 0D757DZ | Dilation of Esophagus with Intraluminal Device, Via Natural or Artificial Opening |
| | | 0D757ZZ | Dilation of Esophagus, Via Natural or Artificial Opening |
| | | 0D758DZ | Dilation of Esophagus with Intraluminal Device, Via Natural or Artificial Opening Endoscopic |
| | | 0D758ZZ | Dilation esophagus nat/art endo |
| | | 0D768DZ | Dilation of Stomach with Intraluminal Device, Via Natural or Artificial Opening Endoscopic |
| | | 0D768ZZ | Dilation stomach nat/art op endo |
| | | 0D778DZ | Dilation of Stomach, Pylorus with Intraluminal Device, Via Natural or Artificial Opening Endoscopic |
| | | 0D778ZZ | Dilation of Stomach, Pylorus, Via Natural or Artificial Opening Endoscopic |
| | | 0D788DZ | Dilation of Small Intestine with Intraluminal Device, Via Natural or Artificial Opening Endoscopic |
| | | 0D788ZZ | Dilation of Small Intestine, Via Natural or Artificial Opening Endoscopic |
| | | 0D798DZ | Dilation of Duodenum with Intraluminal Device, Via Natural or Artificial Opening Endoscopic |
| | | 0D7A8DZ | Dilation of Jejunum with Intraluminal Device, Via Natural or Artificial Opening Endoscopic |
| | | 0D7A8ZZ | Dilation of Jejunum, Via Natural or Artificial Opening Endoscopic |
| | | 0D7B8DZ | Dilation of Ileum with Intraluminal Device, Via Natural or Artificial Opening Endoscopic |
| | | 0D7B8ZZ | Dilation of Ileum, Via Natural or Artificial Opening Endoscopic |
| | | 0D7C8DZ | Dilation of Ileocecal Valve with Intraluminal Device, Via Natural or Artificial Opening Endoscopic |
| | | 0D7C8ZZ | Dilation of Ileocecal Valve, Via Natural or Artificial Opening Endoscopic |
| | | 0D7E8DZ | Dilation of Large Intestine with Intraluminal Device, Via Natural or Artificial Opening Endoscopic |
| | | 0D7F8DZ | Dilation of Right Large Intestine with Intraluminal Device, Via Natural or Artificial Opening Endoscopic |
| | | 0D7G8DZ | Dilation of Left Large Intestine with Intraluminal Device, Via Natural or Artificial Opening Endoscopic |
| | | 0D7H8DZ | Dilation of Cecum with Intraluminal Device, Via Natural or Artificial Opening Endoscopic |
| | | 0D7K8DZ | Dilation of Ascending Colon with Intraluminal Device, Via Natural or Artificial Opening Endoscopic |
| | | 0D7L8DZ | Dilation of Transverse Colon with Intraluminal Device, Via Natural or Artificial Opening Endoscopic |
| | | 0D7M8DZ | Dilation of Descending Colon with Intraluminal Device, Via Natural or Artificial Opening Endoscopic |
| | | 0D7N8DZ | Dilation of Sigmoid Colon with Intraluminal Device, Via Natural or Artificial Opening Endoscopic |
| | | 0D958ZX | Drainage of Esophagus, Via Natural or Artificial Opening Endoscopic, Diagnostic |
| | | 0D968ZZ | Drainage of Stomach, Via Natural or Artificial Opening Endoscopic |
| | | 0D988ZZ | Drainage of Small Intestine, Via Natural or Artificial Opening Endoscopic |
| | | 0D9A8ZZ | Drainage of Jejunum, Via Natural or Artificial Opening Endoscopic |
| | | 0D9B8ZX | Drainage of Ileum, Via Natural or Artificial Opening Endoscopic, Diagnostic |
| | | 0D9B8ZZ | Drainage of Ileum, Via Natural or Artificial Opening Endoscopic |
| | | 0D9C80Z | Drainage of Ileocecal Valve with Drainage Device, Via Natural or Artificial Opening Endoscopic |
| | | 0D9C8ZX | Drainage of Ileocecal Valve, Via Natural or Artificial Opening Endoscopic, Diagnostic |
| | | 0D9C8ZZ | Drainage of Ileocecal Valve, Via Natural or Artificial Opening Endoscopic |
| | | 0DB18ZX | Excision of Upper Esophagus, Via Natural or Artificial Opening Endoscopic, Diagnostic |
| | | 0DB28ZX | Excision of Middle Esophagus, Via Natural or Artificial Opening Endoscopic, Diagnostic |
| | | 0DB38ZX | Excision of Lower Esophagus, Via Natural or Artificial Opening Endoscopic, Diagnostic |
| | | 0DB48ZX | Excision of Esophagogastric Junction, Via Natural or Artificial Opening Endoscopic, Diagnostic |
| | | 0DB58ZX | Excision of Esophagus, Via Natural or Artificial Opening Endoscopic, Diagnostic |
| | | 0DB78ZZ | Excision of Stomach, Pylorus, Via Natural or Artificial Opening Endoscopic |
| | | 0DB88ZX | Excision of Small Intestine, Via Natural or Artificial Opening Endoscopic, Diagnostic |
| | | 0DB98ZX | Excision of Duodenum, Via Natural or Artificial Opening Endoscopic, Diagnostic |
| | | 0DBA8ZX | Excision of Jejunum, Via Natural or Artificial Opening Endoscopic, Diagnostic |
| | | 0DBB8ZX | Excision of Ileum, Via Natural or Artificial Opening Endoscopic, Diagnostic |

© 2019 Lewis KH et al. *JAMA Network Open*.

| | | | |
|---|---|---|---|
| ENDOSCOPY | ICD-10 Procedure | 0DBC8ZX | Excision of Ileocecal Valve, Via Natural or Artificial Opening Endoscopic, Diagnostic |
| | | 0DD18ZX | Extraction of Upper Esophagus, Via Natural or Artificial Opening Endoscopic, Diagnostic |
| | | 0DD28ZX | Extraction of Middle Esophagus, Via Natural or Artificial Opening Endoscopic, Diagnostic |
| | | 0DD38ZX | Extraction of Lower Esophagus, Via Natural or Artificial Opening Endoscopic, Diagnostic |
| | | 0DD48ZX | Extraction of Esophagogastric Junction, Via Natural or Artificial Opening Endoscopic, Diagnostic |
| | | 0DD58ZX | Extraction of Esophagus, Via Natural or Artificial Opening Endoscopic, Diagnostic |
| | | 0DD88ZX | Extraction of Small Intestine, Via Natural or Artificial Opening Endoscopic, Diagnostic |
| | | 0DD98ZX | Extraction of Duodenum, Via Natural or Artificial Opening Endoscopic, Diagnostic |
| | | 0DDA8ZX | Extraction of Jejunum, Via Natural or Artificial Opening Endoscopic, Diagnostic |
| | | 0DDB8ZX | Extraction of Ileum, Via Natural or Artificial Opening Endoscopic, Diagnostic |
| | | 0DDC8ZX | Extraction of Ileocecal Valve, Via Natural or Artificial Opening Endoscopic, Diagnostic |
| | | 0DF68ZZ | Fragmentation in Stomach, Via Natural or Artificial Opening Endoscopic |
| | | 0DF88ZZ | Fragmentation in Small Intestine, Via Natural or Artificial Opening Endoscopic |
| | | 0DF98ZZ | Fragmentation in Duodenum, Via Natural or Artificial Opening Endoscopic |
| | | 0DFA8ZZ | Fragmentation in Jejunum, Via Natural or Artificial Opening Endoscopic |
| | | 0DFB8ZZ | Fragmentation in Ileum, Via Natural or Artificial Opening Endoscopic |
| | | 0DH58DZ | Insertion of Intraluminal Device into Esophagus, Via Natural or Artificial Opening Endoscopic |
| | | 0DH68DZ | Insertion of Intraluminal Device into Stomach, Via Natural or Artificial Opening Endoscopic |
| | | 0DH88DZ | Insertion of Intraluminal Device into Small Intestine, Via Natural or Artificial Opening Endoscopic |
| | | 0DH98DZ | Insertion of Intraluminal Device into Duodenum, Via Natural or Artificial Opening Endoscopic |
| | | 0DHA8DZ | Insertion of Intraluminal Device into Jejunum, Via Natural or Artificial Opening Endoscopic |
| | | 0DHB8DZ | Insertion of Intraluminal Device into Ileum, Via Natural or Artificial Opening Endoscopic |
| | | 0DHE8DZ | Insertion of Intraluminal Device into Large Intestine, Via Natural or Artificial Opening Endoscopic |
| | | 0DHP8DZ | Insertion of Intraluminal Device into Rectum, Via Natural or Artificial Opening Endoscopic |
| | | 0DJ08ZZ | Inspect upper intestine tract endo |
| | | 0DJ0XZZ | Inspection of Upper Intestinal Tract, External Approach |
| | | 0DJ68ZZ | Inspection of Stomach, Via Natural or Artificial Opening Endoscopic |
| | | 0DL68DZ | Occlusion of Stomach with Intraluminal Device, Via Natural or Artificial Opening Endoscopic |
| | | 0DL68ZZ | Occlusion of Stomach, Via Natural or Artificial Opening Endoscopic |
| | | 0DL78DZ | Occlusion of Stomach, Pylorus with Intraluminal Device, Via Natural or Artificial Opening Endoscopic |
| | | 0DL78ZZ | Occlusion of Stomach, Pylorus, Via Natural or Artificial Opening Endoscopic |
| | | 0DL88DZ | Occlusion of Small Intestine with Intraluminal Device, Via Natural or Artificial Opening Endoscopic |
| | | 0DL88ZZ | Occlusion of Small Intestine, Via Natural or Artificial Opening Endoscopic |
| | | 0DL98DZ | Occlusion of Duodenum with Intraluminal Device, Via Natural or Artificial Opening Endoscopic |
| | | 0DL98ZZ | Occlusion of Duodenum, Via Natural or Artificial Opening Endoscopic |
| | | 0DLA8DZ | Occlusion of Jejunum with Intraluminal Device, Via Natural or Artificial Opening Endoscopic |
| | | 0DLA8ZZ | Occlusion of Jejunum, Via Natural or Artificial Opening Endoscopic |
| | | 0DLB8DZ | Occlusion of Ileum with Intraluminal Device, Via Natural or Artificial Opening Endoscopic |
| | | 0DLB8ZZ | Occlusion of Ileum, Via Natural or Artificial Opening Endoscopic |
| | | 0DLC8DZ | Occlusion of Ileocecal Valve with Intraluminal Device, Via Natural or Artificial Opening Endoscopic |
| | | 0DLC8ZZ | Occlusion of Ileocecal Valve, Via Natural or Artificial Opening Endoscopic |
| | | 0DN68ZZ | Release Stomach, Via Natural or Artificial Opening Endoscopic |
| | | 0DN78ZZ | Release Stomach, Pylorus, Via Natural or Artificial Opening Endoscopic |
| | | 0DP087Z | Removal of Autologous Tissue Substitute from Upper Int Tract, Via Natural or Artificial Opening Endoscopic |
| | | 0DP08CZ | Removal of Extraluminal Device from Upper Intestinal Tract, Via Natural or Artificial Opening Endoscopic |
| | | 0DP08JZ | Removal of Synthetic Substitute from Upper Intestinal Tract, Via Natural or Artificial Opening Endoscopic |
| | | 0DP08KZ | Removal of Nonautologous Tissue Substitute from Upper Int Tract, Via Natural or Artificial Opening Endoscopic |
| | | 0DP687Z | Removal of Autologous Tissue Substitute from Stomach, Via Natural or Artificial Opening Endoscopic |
| | | 0DP68CZ | Removal of Extraluminal Device from Stomach, Via Natural or Artificial Opening Endoscopic |
| | | 0DP68JZ | Removal of Synthetic Substitute from Stomach, Via Natural or Artificial Opening Endoscopic |
| | | 0DP68KZ | Removal of Nonautologous Tissue Substitute from Stomach, Via Natural or Artificial Opening Endoscopic |
| | | 0DQ68ZZ | Repair Stomach, Via Natural or Artificial Opening Endoscopic |
| | | 0DU687Z | Supplement Stomach with Autologous Tissue Substitute, Via Natural or Artificial Opening Endoscopic |
| | | 0DU68JZ | Supplement Stomach with Synthetic Substitute, Via Natural or Artificial Opening Endoscopic |
| | | 0DU68KZ | Supplement Stomach with Nonautologous Tissue Substitute, Via Natural or Artificial Opening Endoscopic |
| | | 0DU787Z | Supplement Stomach, Pylorus with Autologous Tissue Substitute, Via Natural or Artificial Opening Endoscopic |
| | | 0DU78JZ | Supplement Stomach, Pylorus with Synthetic Substitute, Via Natural or Artificial Opening Endoscopic |
| | | 0DU78KZ | Supplement Stomach, Pylorus with Nonautol Tissue Substitute, Via Natural or Artificial Opening Endoscopic |
| | | 0DU887Z | Supplement Small Intestine with Autologous Tissue Substitute, Via Natural or Artificial Opening Endoscopic |
| | | 0DU88JZ | Supplement Small Intestine with Synthetic Substitute, Via Natural or Artificial Opening Endoscopic |
| | | 0DU88KZ | Supplement Small Intestine with Nonautologous Tissue Substitute, Via Natural or Artificial Opening Endoscopic |
| | | 0DU987Z | Suppl duodenum auto tissue sub endo |
| | | 0DU98JZ | Supplement Duodenum with Synthetic Substitute, Via Natural or Artificial Opening Endoscopic |
| | | 0DU98KZ | Supplement Duodenum with Nonautologous Tissue Substitute, Via Natural or Artificial Opening Endoscopic |
| | | 0DUA8JZ | Supplement Jejunum with Synthetic Substitute, Via Natural or Artificial Opening Endoscopic |
| | | 0DUA8KZ | Supplement Jejunum with Nonautologous Tissue Substitute, Via Natural or Artificial Opening Endoscopic |
| | | 0DUB87Z | Supplement Ileum with Autologous Tissue Substitute, Via Natural or Artificial Opening Endoscopic |
| | | 0DUB8JZ | Supplement Ileum with Synthetic Substitute, Via Natural or Artificial Opening Endoscopic |
| | | 0DUB8KZ | Supplement Ileum with Nonautologous Tissue Substitute, Via Natural or Artificial Opening Endoscopic |
| | | 0DUC87Z | Supplement Ileocecal Valve with Autologous Tissue Substitute, Via Natural or Artificial Opening Endoscopic |
| | | 0DUC8JZ | Supplement Ileocecal Valve with Synthetic Substitute, Via Natural or Artificial Opening Endoscopic |
| | | 0DUC8KZ | Supplement Ileocecal Valve with Nonautologous Tissue Substitute, Via Natural or Artificial Opening Endoscopic |
| | | 0DW080Z | Revision of Drainage Device in Upper Intestinal Tract, Via Natural or Artificial Opening Endoscopic |
| | | 0DW082Z | Revision of Monitoring Device in Upper Intestinal Tract, Via Natural or Artificial Opening Endoscopic |
| | | 0DW087Z | Revision of Autologous Tissue Substitute in Upper Intestinal Tract, Via Natural or Artificial Opening Endoscopic |
| | | 0DW08CZ | Revision of Extraluminal Device in Upper Intestinal Tract, Via Natural or Artificial Opening Endoscopic |
| | | 0DW08DZ | Revision of Intraluminal Device in Upper Intestinal Tract, Via Natural or Artificial Opening Endoscopic |
| | | 0DW08JZ | Revision of Synthetic Substitute in Upper Intestinal Tract, Via Natural or Artificial Opening Endoscopic |
| | | 0DW08KZ | Revision of Nonautol Tissue Substitute in Upper Intestinal Tract, Via Natural or Artificial Opening Endoscopic |
| | | 0F758DZ | Dilation of Right Hepatic Duct with Intraluminal Device, Via Natural or Artificial Opening Endoscopic |

© 2019 Lewis KH et al. *JAMA Network Open*.

| | | | |
|---|---|---|---|
| ENDOSCOPY | ICD-10 Procedure | 0F768DZ | Dilation of Left Hepatic Duct with Intraluminal Device, Via Natural or Artificial Opening Endoscopic |
| | | 0F778DZ | Dilation of Common Hepatic Duct with Intraluminal Device, Via Natural or Artificial Opening Endoscopic |
| | | 0F788DZ | Dilation of Cystic Duct with Intraluminal Device, Via Natural or Artificial Opening Endoscopic |
| | | 0F798DZ | Dilation of Common Bile Duct with Intraluminal Device, Via Natural or Artificial Opening Endoscopic |
| | | 0F9C80Z | Drainage of Ampulla of Vater with Drainage Device, Via Natural or Artificial Opening Endoscopic |
| | | 0FC58ZZ | Extirpation of Matter from Right Hepatic Duct, Via Natural or Artificial Opening Endoscopic |
| | | 0FC68ZZ | Extirpation of Matter from Left Hepatic Duct, Via Natural or Artificial Opening Endoscopic |
| | | 0FC78ZZ | Extirpation of Matter from Common Hepatic Duct, Via Natural or Artificial Opening Endoscopic |
| | | 0FC88ZZ | Extirpation of Matter from Cystic Duct, Via Natural or Artificial Opening Endoscopic |
| | | 0FC98ZZ | Extirpation of Matter from Common Bile Duct, Via Natural or Artificial Opening Endoscopic |
| | | 0FCC8ZZ | Extirpation of Matter from Ampulla of Vater, Via Natural or Artificial Opening Endoscopic |
| | | 0FF58ZZ | Fragmentation in Right Hepatic Duct, Via Natural or Artificial Opening Endoscopic |
| | | 0FF68ZZ | Fragmentation in Left Hepatic Duct, Via Natural or Artificial Opening Endoscopic |
| | | 0FF78ZZ | Fragmentation in Common Hepatic Duct, Via Natural or Artificial Opening Endoscopic |
| | | 0FF88ZZ | Fragmentation in Cystic Duct, Via Natural or Artificial Opening Endoscopic |
| | | 0FF98ZZ | Fragmentation in Common Bile Duct, Via Natural or Artificial Opening Endoscopic |
| | | 0FFC8ZZ | Fragmentation in Ampulla of Vater, Via Natural or Artificial Opening Endoscopic |
| | | 0FHB8DZ | Insertion of Intraluminal Device into Hepatobiliary Duct, Via Natural or Artificial Opening Endoscopic |
| | | 0FJ48ZZ | Inspection of Gallbladder, Via Natural or Artificial Opening Endoscopic |
| | | 0FJG8ZZ | Inspection of Pancreas, Via Natural or Artificial Opening Endoscopic |
| | | 0FN48ZZ | Release Gallbladder, Via Natural or Artificial Opening Endoscopic |
| | | 0FN58ZZ | Release Right Hepatic Duct, Via Natural or Artificial Opening Endoscopic |
| | | 0FN68ZZ | Release Left Hepatic Duct, Via Natural or Artificial Opening Endoscopic |
| | | 0FN78ZZ | Release Common Hepatic Duct, Via Natural or Artificial Opening Endoscopic |
| | | 0FN88ZZ | Release Cystic Duct, Via Natural or Artificial Opening Endoscopic |
| | | 0FN98ZZ | Release Common Bile Duct, Via Natural or Artificial Opening Endoscopic |
| | | 0FNC8ZZ | Release Ampulla of Vater, Via Natural or Artificial Opening Endoscopic |
| | | 0FND8ZZ | Release Pancreatic Duct, Via Natural or Artificial Opening Endoscopic |
| | | 0FNF8ZZ | Release Accessory Pancreatic Duct, Via Natural or Artificial Opening Endoscopic |
| | | 0W3P8ZZ | Control Bleeding in Gastrointestinal Tract, Via Natural or Artificial Opening Endoscopic |
| ENTERAL ACCESS | CPT | 43246 | EGD place gastrostomy tube |
| | | 43653 | Laparoscopy, gastrostomy |
| | | 43750 | Place gastrostomy tube |
| | | 43752 | Nasal/orogastric tube placement |
| | | 43760 | Change gastrostomy tube |
| | | 43761 | Reposition gastrostomy tube |
| | | 43830 | Place gastrostomy tube |
| | | 43832 | Place gastrostomy tube |
| | | 44015 | Tube/needle catheter jejunostomy |
| | | 44186 | Lap, jejunostomy |
| | | 44500 | Introdxn proc on intestines |
| | | 49440 | Place gastrostomy tube perc |
| | | 49441 | Place duod/jej tube perc |
| | | 49446 | Change gtube to gj perc |
| | | 49450 | Replace g/c tube perc |
| | | 49451 | Replace duod/jej tube perc |
| | | 49452 | Replace g-j tube perc |
| ENTERAL ACCESS | ICD-9 Procedure | 43.11 | Percutaneous gastrostomy/peg tube |
| | | 43.19 | Other gastrostomy |
| | | 46.32 | Percutaneous jejunostomy |
| | | 46.39 | Other enterostomy |
| ENTERAL ACCESS | ICD-10 Procedure | 0DH50UZ | Insertion of Feeding Device into Esophagus, Open Approach |
| | | 0DH53UZ | Insertion of Feeding Device into Esophagus, Percutaneous Approach |
| | | 0DH54UZ | Insertion of Feeding Device into Esophagus, Percutaneous Endoscopic Approach |
| | | 0DH57UZ | Insertion of Feeding Device into Esophagus, Via Natural or Artificial Opening |
| | | 0DH58UZ | Insertion of Feeding Device into Esophagus, Via Natural or Artificial Opening Endoscopic |
| | | 0DH60UZ | Insert feed device stomach open |
| | | 0DH63UZ | Insertion of Feeding Device into Stomach, Percutaneous Approach |
| | | 0DH64UZ | Insert Feed Device Stomach Perq Endo |
| | | 0DH80UZ | Insertion of Feeding Device into Small Intestine, Open Approach |
| | | 0DH83UZ | Insertion of Feeding Device into Small Intestine, Percutaneous Approach |
| | | 0DH84UZ | Insertion of Feeding Device into Small Intestine, Percutaneous Endoscopic Approach |
| | | 0DH87UZ | Insertion of Feeding Device into Small Intestine, Via Natural or Artificial Opening |
| | | 0DH88UZ | Insertion of Feeding Device into Small Intestine, Via Natural or Artificial Opening Endoscopic |
| | | 0DH90UZ | Insertion of Feeding Device into Duodenum, Open Approach |
| | | 0DH93UZ | Insertion of Feeding Device into Duodenum, Percutaneous Approach |
| | | 0DH94UZ | Insertion of Feeding Device into Duodenum, Percutaneous Endoscopic Approach |
| | | 0DH97UZ | Insertion of Feeding Device into Duodenum, Via Natural or Artificial Opening |
| | | 0DH98UZ | Insertion of Feeding Device into Duodenum, Via Natural or Artificial Opening Endoscopic |
| | | 0DHA0UZ | Insertion of Feeding Device into Jejunum, Open Approach |
| | | 0DHA3UZ | Insert Feed Device Jejun Perq Approach |
| | | 0DHA7UZ | Insertion of Feeding Device into Jejunum, Via Natural or Artificial Opening |
| | | 0DHB0UZ | Insertion of Feeding Device into Ileum, Open Approach |
| | | 0DHB3UZ | Insertion of Feeding Device into Ileum, Percutaneous Approach |
| | | 0DHB4UZ | Insertion of Feeding Device into Ileum, Percutaneous Endoscopic Approach |
| | | 0DHB7UZ | Insertion of Feeding Device into Ileum, Via Natural or Artificial Opening |
| | | 0DHB8UZ | Insertion of Feeding Device into Ileum, Via Natural or Artificial Opening Endoscopic |
| | | 0DP00UZ | Removal of Feeding Device from Upper Intestinal Tract, Open Approach |
| | | 0DP03UZ | Removal of Feeding Device from Upper Intestinal Tract, Percutaneous Approach |
| | | 0DP04UZ | Removal of Feeding Device from Upper Intestinal Tract, Percutaneous Endoscopic Approach |

© 2019 Lewis KH et al. *JAMA Network Open*.

| | | | |
|---|---|---|---|
| ENTERAL ACCESS | ICD-10 Procedure | 0DP07UZ<br>0DP08UZ<br>0DP60UZ<br>0DP63UZ<br>0DP64UZ<br>0DP68UZ<br>0DW00UZ<br>0DW03UZ<br>0DW07UZ<br>0DW08UZ<br>0DW60UZ<br>0DW63UZ<br>0DW64UZ<br>0DW67UZ<br>0DW68UZ | Removal of Feeding Device from Upper Intestinal Tract, Via Natural or Artificial Opening<br>Removal of Feeding Device from Upper Intestinal Tract, Via Natural or Artificial Opening Endoscopic<br>Removal of Feeding Device from Stomach, Open Approach<br>Removal of Feeding Device from Stomach, Percutaneous Approach<br>Removal of Feeding Device from Stomach, Percutaneous Endoscopic Approach<br>Removal of Feeding Device from Stomach, Via Natural or Artificial Opening Endoscopic<br>Revision of Feeding Device in Upper Intestinal Tract, Open Approach<br>Revision of Feeding Device in Upper Intestinal Tract, Percutaneous Approach<br>Revision of Feeding Device in Upper Intestinal Tract, Via Natural or Artificial Opening<br>Revision of Feeding Device in Upper Intestinal Tract, Via Natural or Artificial Opening Endoscopic<br>Revision of Feeding Device in Stomach, Open Approach<br>Revision of Feeding Device in Stomach, Percutaneous Approach<br>Revision of Feeding Device in Stomach, Percutaneous Endoscopic Approach<br>Revision of Feeding Device in Stomach, Via Natural or Artificial Opening<br>Revision of Feeding Device in Stomach, Via Natural or Artificial Opening Endoscopic |
| ABDOMINAL WALL HERNIA REPAIR | CPT | 49560<br>49561<br>49565<br>49566<br>49568<br>49570<br>49572<br>49585<br>49587<br>49590<br>49651<br>49652<br>49653<br>49654<br>49655<br>49656<br>49657<br>49659<br>49900 | Rpr ventral hern init, reduc<br>Rpr ventral hern init, block<br>Rerepair ventrl hern, reduce<br>Rerepair ventral hernia, block<br>Hernia repair w/mesh<br>Rpr epigastric hern, reduce<br>Hernioplasty<br>Rpr umbil hern, reduc > 5 yr<br>Rpr umbil hern, block > 5 yr<br>Hernioplasty<br>Hernia laparoscope<br>Rap/vent abd hernia repair<br>Rap/vent abd hernia proc comp<br>Rap inc hernia repair<br>Rap inc hern repair comp<br>Rap inc hernia repair recur<br>Rap inc hern recur comp<br>Laparoscopic proc, hernia repair<br>Suture, secondary, of abdominal wall for evisceration or dehiscence |
| ABDOMINAL WALL HERNIA REPAIR | ICD-9 procedure | 53.0<br>53.41<br>53.42<br>53.43<br>53.49<br>53.5<br>53.51<br>53.59<br>53.6<br>53.61<br>53.62<br>53.63<br>53.69<br>53.9 | Repair of hernia<br>Oth & opn rep umb hernia w/gft/pros<br>Lap rep umb hernia w/graft/prosth<br>Other lap umbilical herniorraphy<br>Other open umbilical herniorrhaphy<br>Repair of other anterior hernia<br>Incisional hernia repair<br>Repair oth hernia anterior abd wall<br>Repair of other anterior hernia w graft<br>Oth & opn inc hernia rep w/gft/pros<br>Lap incisional hernia rep gft/pros<br>Oth lap rep oth hernia ant abd wall<br>Oth & opn rep oth hern ant aw-graft<br>Other hernia repair |
| ABDOMINAL WALL HERNIA REPAIR | ICD-10 procedure | 0WQF0ZZ<br>0WQF3ZZ<br>0WQF4ZZ<br>0WQFXZZ<br>0WUF07Z<br>0WUF0JZ<br>0WUF0KZ<br>0WUF47Z<br>0WUF4JZ<br>0WUF4KZ | Repair Abdominal Wall, Open Approach<br>Repair Abdominal Wall, Percutaneous Approach<br>Repair Abdominal Wall, Percutaneous Endoscopic Approach<br>Repair Abdominal Wall, External Approach<br>Supplement Abdominal Wall with Autologous Tissue Substitute, Open Approach<br>Supplement Abdominal Wall with Synthetic Substitute, Open Approach<br>Supplement Abdominal Wall with Nonautologous Tissue Substitute, Open Approach<br>Supplement Abdominal Wall with Autologous Tissue Substitute, Percutaneous Endoscopic Approach<br>Supplement Abdominal Wall with Synthetic Substitute, Percutaneous Endoscopic Approach<br>Supplement Abdominal Wall with Nonautologous Tissue Substitute, Percutaneous Endoscopic Approach |
| OTHER NONOPERATIVE INTERVENTION | CPT | 10140<br>49041<br>49061<br>49080<br>49082<br>49083<br>49084<br>49324<br>49406<br>49420<br>49422<br>49423<br>75989 | Drainage of hematoma/fluid<br>Drain, percut, abdom abscess<br>Drain, percut, retroper absc<br>Puncture peritoneal cavity<br>Abdominal paracentesis<br>Abdominal paracentesis with imaging<br>Peritoneal lavage<br>Lap insert ip cath<br>Image guided drain of app abscess<br>Insert abdominal drain temp<br>Remove tunneled ip cath<br>Exchange drainage catheter<br>Abscess drainage under xray |
| OTHER NONOPERATIVE INTERVENTION | ICD-9 Procedure | 54.91 | Percutaneous abdominal drainage |
| OTHER NONOPERATIVE INTERVENTION | ICD-10 Procedure | 0D583ZZ<br>0D5A3ZZ<br>0D5B3ZZ<br>0D5C3ZZ<br>0D5U3ZZ<br>0D5V3ZZ | Destruction of Small Intestine, Percutaneous Approach<br>Destruction of Jejunum, Percutaneous Approach<br>Destruction of Ileum, Percutaneous Approach<br>Destruction of Ileocecal Valve, Percutaneous Approach<br>Destruction of Omentum, Percutaneous Approach<br>Destruction of Mesentery, Percutaneous Approach |

© 2019 Lewis KH et al. *JAMA Network Open*.

| | | | |
|---|---|---|---|
| OTHER NONOPERATIVE INTERVENTION | ICD-10 Procedure | 0D5W3ZZ | Destruction of Peritoneum, Percutaneous Approach |
| | | 0D763DZ | Dilation of Stomach with Intraluminal Device, Percutaneous Approach |
| | | 0D763ZZ | Dilation of Stomach, Percutaneous Approach |
| | | 0D773DZ | Dilation of Stomach, Pylorus with Intraluminal Device, Percutaneous Approach |
| | | 0D913ZX | Drainage of Upper Esophagus, Percutaneous Approach, Diagnostic |
| | | 0D923ZX | Drainage of Middle Esophagus, Percutaneous Approach, Diagnostic |
| | | 0D933ZX | Drainage of Lower Esophagus, Percutaneous Approach, Diagnostic |
| | | 0D943ZX | Drainage of Esophagogastric Junction, Percutaneous Approach, Diagnostic |
| | | 0D953ZX | Drainage of Esophagus, Percutaneous Approach, Diagnostic |
| | | 0D983ZX | Drainage of Small Intestine, Percutaneous Approach, Diagnostic |
| | | 0D993ZX | Drainage of Duodenum, Percutaneous Approach, Diagnostic |
| | | 0D9C3ZX | Drainage of Ileocecal Valve, Percutaneous Approach, Diagnostic |
| | | 0DB23ZX | Excision of Middle Esophagus, Percutaneous Approach, Diagnostic |
| | | 0DB33ZX | Excision of Lower Esophagus, Percutaneous Approach, Diagnostic |
| | | 0W9F3ZZ | Drainage abdominal wall perq |
| | | 0W9G3ZZ | Drainage peritoneal cavity perq |
| | | 0WWH30Z | Revision of Drainage Device in Retroperitoneum, Percutaneous Approach |
| | | 0WWH3YZ | Revision of Other Device in Retroperitoneum, Percutaneous Approach |
| | | 3E1M38X | Irrigation of Peritoneal Cavity using Irrigating Substance, Percutaneous Approach, Diagnostic |
| | | 3E1M38Z | Irrigation of Peritoneal Cavity using Irrigating Substance, Percutaneous Approach |
| OTHER ABDOMINAL OPERATION | CPT | 22999 | Abdomen surgery procedure |
| | | 39599 | Unlisted px diaphragm |
| | | 43123 | Partial removal of esophagus |
| | | 43279 | Esophagomyotomy |
| | | 43280 | Laparoscopic fundoplasty |
| | | 43281 | Lap paraesophag hernia repair |
| | | 43282 | Lap paraesophag hernia rpr w/mesh |
| | | 43289 | Laparoscope proc, esoph |
| | | 43300 | Repair esoph |
| | | 43305 | Repair esoph |
| | | 43310 | Repair esoph |
| | | 43312 | Repair esoph |
| | | 43327 | Fundoplasty |
| | | 43328 | Fundoplasty |
| | | 43330 | Esophagomyotomy |
| | | 43331 | Esophagomyotomy |
| | | 43332 | Tranabd esoph hiat hernia repair |
| | | 43333 | Paraesophageal hernia repair |
| | | 43334 | Paraesophageal hernia repair |
| | | 43335 | Paraesophageal hernia repair |
| | | 43336 | Paraesophageal hernia repair |
| | | 43337 | Paraesophageal hernia repair |
| | | 43340 | Esophagojejunostomy |
| | | 43341 | Esophagojejunostomy |
| | | 43499 | Esophagus surgery procedure |
| | | 43500 | Surgical opening of stomach |
| | | 43501 | Gastrotomy w suture bldg ulcer |
| | | 43520 | Pyloromyotomy |
| | | 43610 | Gastrotomy w ulcer excision |
| | | 43640 | Pyloroplasty and vagotomy |
| | | 43651 | Laparoscopy, vagus nerve |
| | | 43659 | Laparoscope procedure, stomach |
| | | 43800 | Pyloroplasty |
| | | 43840 | Repair of stomach lesion |
| | | 43870 | Repair stomach opening |
| | | 43999 | Stomach surgery procedure |
| | | 44005 | Freeing of bowel adhesion |
| | | 44020 | Explore small intestine |
| | | 44021 | Decompress small bowel |
| | | 44050 | Reduce bowel obstruction |
| | | 44055 | Correct malrotation of bowel |
| | | 44120 | Removal of small intestine |
| | | 44121 | Enterectomy resxn sm intestine |
| | | 44125 | Enterectomy resxn sm intestine |
| | | 44130 | Bowel to bowel fusion |
| | | 44180 | Lap, enterolysis |
| | | 44200 | Lap fundoplication w/ enterolysis |
| | | 44202 | Lap, enterectomy |
| | | 44203 | Lap resect s/intestine addl |
| | | 44238 | Laparoscope proc, intestine |
| | | 44300 | Open bowel to skin |
| | | 44310 | Ileostomy/jejunostomy |
| | | 44602 | Suture small intestine |
| | | 44603 | Suture small intestine |
| | | 44615 | Intestinal stricturoplasty |
| | | 44620 | Repair bowel opening |
| | | 44640 | Closure enterocutaneous fistula |
| | | 44680 | Intestinal plication |
| | | 44799 | Unlisted px small intestine |
| | | 44800 | Excision of bowel pouch |

© 2019 Lewis KH et al. *JAMA Network Open*.

| | | | |
|---|---|---|---|
| OTHER ABDOMINAL OPERATION | CPT | 44850 | Repair of mesentary |
| | | 44899 | Bowel surgery procedure |
| | | 47011 | Percut drain, liver lesion |
| | | 47379 | Laparoscope procedure, liver |
| | | 48000 | Place peripancreatic drain |
| | | 49000 | Exploration of abdomen |
| | | 49002 | Reopening of abdomen |
| | | 49010 | Exploration behind abdomen |
| | | 49020 | Drainage of abdom abscess open |
| | | 49021 | Drain abdominal abscess |
| | | 49040 | Drain, open, abdom abscess |
| | | 49060 | Drain retroperitoneal abscess |
| | | 49320 | Diag laparo separate proc |
| | | 49321 | Laparoscopy, biopsy |
| | | 49322 | Laparoscopy, aspiration |
| | | 49326 | Lap w/omentopexy add-on |
| | | 49329 | Laparo proc, abdm/per/oment |
| | | 49402 | Remove foreign body, adbomen |
| | | 49904 | Omental flap extra abdom |
| | | 49905 | Omental flap intra-abdom |
| | | 49999 | Abdomen surgery procedure |
| OTHER ABDOMINAL OPERATION | ICD-9 Procedure | 17.41 | Open robotic assisted procedure |
| | | 17.42 | Laparoscopic robotic assisted proc |
| | | 34.83 | Closure of fistula of diaphragm |
| | | 42.81 | Insertion perm tube esophagus |
| | | 42.84 | Repair esoph fistula |
| | | 42.9 | Other operations on esophagus |
| | | 43.0 | Gastrotomy |
| | | 43.42 | Local excision oth lesion/tissue stomach |
| | | 44.01 | Truncal vagotomy |
| | | 44.29 | Other pyloroplasty |
| | | 44.40 | Suture of peptic ulcer nos |
| | | 44.41 | Suture of gastric ulcer site |
| | | 44.42 | Suture of duodenal ulcer site |
| | | 44.49 | Oth control hemorr stomach/duodenum |
| | | 44.61 | Suture of laceration of stomach |
| | | 44.62 | Closure of gastrostomy |
| | | 44.63 | Closure of other gastric fistula |
| | | 44.64 | Gastropexy |
| | | 44.67 | Lap create eg sphinctric competence |
| | | 44.69 | Other repair of stomach |
| | | 44.93 | Insertion of gastric bubble |
| | | 44.99 | Other operations on stomach |
| | | 45.02 | Other incision on small intestine |
| | | 45.19 | Oth dx proc small intestine |
| | | 45.33 | Loc exc les/tiss sm intst no duodum |
| | | 45.34 | Oth destruc les sm intest no duodum |
| | | 45.51 | Isolation segment small intestine |
| | | 45.61 | Mx seg resection small intestine |
| | | 45.62 | Oth part resection sm intestine |
| | | 45.63 | Total removal of small intestine |
| | | 45.90 | Intestinal anastomosis nos |
| | | 45.91 | Sm to sm intestine anastamosis |
| | | 45.92 | Anastom sm intestine rectal stump |
| | | 46.01 | Exteriorization of small intestine |
| | | 46.40 | Revision of intestinal stoma nos |
| | | 46.41 | Revision stoma small intestine |
| | | 46.50 | Closure of intestinal stoma nos |
| | | 46.51 | Closure of stoma of small intestine |
| | | 46.60 | Fixation of intestine nos |
| | | 46.62 | Other fixation of small intestine |
| | | 46.71 | Suture laceration duodenum |
| | | 46.73 | Sut laceration sm intest no duodum |
| | | 46.74 | Clos fist sm intestine no duodenum |
| | | 46.79 | Other repair of intestine |
| | | 46.80 | Intra-abd manipulation intest nos |
| | | 46.81 | Intra-abd manipulation of sm intestine |
| | | 46.85 | Dilation of intestine |
| | | 46.99 | Oth op on intestines |
| | | 53.71 | Lap rep diaph hernia abd approach |
| | | 54.0 | Incision of abdominal wall |
| | | 54.11 | Exploratory laparotomy |
| | | 54.12 | Reopening of recent laparotomy site |
| | | 54.19 | Other laparotomy |
| | | 54.21 | Laparoscopy |
| | | 54.3 | Exc/destruc les abd wall/umbilicus |
| | | 54.4 | Incision/destxn peritoneal tx |
| | | 54.51 | Laparoscopic lysis peritoneal adhesions |
| | | 54.59 | Other lysis of peritoneal adhesions |
| | | 54.61 | Reclos postop disrupt abdom wal |

© 2019 Lewis KH et al. *JAMA Network Open*.

| | | | |
|---|---|---|---|
| OTHER ABDOMINAL OPERATION | ICD-9 Procedure | 54.62 | Delay closure granulating abd wound |
| | | 54.63 | Other suture abdominal wall |
| | | 54.72 | Other repair abdominal wall |
| | | 54.74 | Other repair of omentum |
| | | 54.75 | Other repair of mesentary |
| | | 54.95 | Incision of peritoneum |
| | | 54.99 | Other operations abdominal region |
| OTHER ABDOMINAL OPERATION | ICD-10 Procedure | 008Q0ZZ | Division of Vagus Nerve, Open Approach |
| | | 008Q3ZZ | Division of Vagus Nerve, Percutaneous Approach |
| | | 008Q4ZZ | Division of Vagus Nerve, Percutaneous Endoscopic Approach |
| | | 07JP0ZZ | Inspection of Spleen, Open Approach |
| | | 0BQT4ZZ | Repair Diaphragm, Percutaneous Endoscopic Approach |
| | | 0BRT47Z | Replacement of Diaphragm with Autologous Tissue Substitute, Percutaneous Endoscopic Approach |
| | | 0BRT4JZ | Replacement of Diaphragm with Synthetic Substitute, Percutaneous Endoscopic Approach |
| | | 0BRT4KZ | Replacement of Diaphragm with Nonautologous Tissue Substitute, Percutaneous Endoscopic Approach |
| | | 0BUT47Z | Supplement Diaphragm with Autologous Tissue Substitute, Percutaneous Endoscopic Approach |
| | | 0BUT4JZ | Supplement Diaphragm with Synthetic Substitute, Percutaneous Endoscopic Approach |
| | | 0BUT4KZ | Supplement Diaphragm with Nonautologous Tissue Substitute, Percutaneous Endoscopic Approach |
| | | 0D16074 | Bypass Stomach to Cutaneous with Autologous Tissue Substitute, Open Approach |
| | | 0D160J4 | Bypass Stomach to Cutaneous with Synthetic Substitute, Open Approach |
| | | 0D160K4 | Bypass Stomach to Cutaneous with Nonautologous Tissue Substitute, Open Approach |
| | | 0D160Z4 | Bypass Stomach to Cutaneous, Open Approach |
| | | 0D163J4 | Bypass Stomach to Cutaneous with Synthetic Substitute, Percutaneous Approach |
| | | 0D16474 | Bypass Stomach to Cutaneous with Autologous Tissue Substitute, Percutaneous Endoscopic Approach |
| | | 0D164J4 | Bypass Stomach to Cutaneous with Synthetic Substitute, Percutaneous Endoscopic Approach |
| | | 0D164K4 | Bypass Stomach to Cutaneous with Nonautologous Tissue Substitute, Percutaneous Endoscopic Approach |
| | | 0D164Z4 | Bypass Stomach to Cutaneous, Percutaneous Endoscopic Approach |
| | | 0D19074 | Bypass Duodenum to Cutaneous with Autologous Tissue Substitute, Open Approach |
| | | 0D19079 | Bypass Duodenum to Duodenum with Autologous Tissue Substitute, Open Approach |
| | | 0D1907A | Bypass Duodenum to Jejunum with Autologous Tissue Substitute, Open Approach |
| | | 0D1907B | Bypass Duodenum to Ileum with Autologous Tissue Substitute, Open Approach |
| | | 0D1907L | Bypass Duodenum to Transverse Colon with Autologous Tissue Substitute, Open Approach |
| | | 0D190J4 | Bypass Duodenum to Cutaneous with Synthetic Substitute, Open Approach |
| | | 0D190J9 | Bypass Duodenum to Duodenum with Synthetic Substitute, Open Approach |
| | | 0D190JA | Bypass Duodenum to Jejunum with Synthetic Substitute, Open Approach |
| | | 0D190JB | Bypass Duodenum to Ileum with Synthetic Substitute, Open Approach |
| | | 0D190JL | Bypass Duodenum to Transverse Colon with Synthetic Substitute, Open Approach |
| | | 0D190K4 | Bypass Duodenum to Cutaneous with Nonautologous Tissue Substitute, Open Approach |
| | | 0D190K9 | Bypass Duodenum to Duodenum with Nonautologous Tissue Substitute, Open Approach |
| | | 0D190KA | Bypass Duodenum to Jejunum with Nonautologous Tissue Substitute, Open Approach |
| | | 0D190KB | Bypass Duodenum to Ileum with Nonautologous Tissue Substitute, Open Approach |
| | | 0D190KL | Bypass Duodenum to Transverse Colon with Nonautologous Tissue Substitute, Open Approach |
| | | 0D190Z4 | Bypass Duodenum to Cutaneous, Open Approach |
| | | 0D190Z9 | Bypass Duodenum to Duodenum, Open Approach |
| | | 0D190ZA | Bypass Duodenum to Jejunum, Open Approach |
| | | 0D190ZB | Bypass Duodenum to Ileum, Open Approach |
| | | 0D190ZL | Bypass Duodenum to Transverse Colon, Open Approach |
| | | 0D193J4 | Bypass Duodenum to Cutaneous with Synthetic Substitute, Percutaneous Approach |
| | | 0D19474 | Bypass Duodenum to Cutaneous with Autologous Tissue Substitute, Percutaneous Endoscopic Approach |
| | | 0D19479 | Bypass Duodenum to Duodenum with Autologous Tissue Substitute, Percutaneous Endoscopic Approach |
| | | 0D1947A | Bypass Duodenum to Jejunum with Autologous Tissue Substitute, Percutaneous Endoscopic Approach |
| | | 0D1947B | Bypass Duodenum to Ileum with Autologous Tissue Substitute, Percutaneous Endoscopic Approach |
| | | 0D1947L | Bypass Duodenum to Transverse Colon with Autologous Tissue Substitute, Percutaneous Endoscopic Approach |
| | | 0D194J4 | Bypass Duodenum to Cutaneous with Synthetic Substitute, Percutaneous Endoscopic Approach |
| | | 0D194J9 | Bypass Duodenum to Duodenum with Synthetic Substitute, Percutaneous Endoscopic Approach |
| | | 0D194JA | Bypass Duodenum to Jejunum with Synthetic Substitute, Percutaneous Endoscopic Approach |
| | | 0D194JB | Bypass Duodenum to Ileum with Synthetic Substitute, Percutaneous Endoscopic Approach |
| | | 0D194JL | Bypass Duodenum to Transverse Colon with Synthetic Substitute, Percutaneous Endoscopic Approach |
| | | 0D194K4 | Bypass Duodenum to Cutaneous with Nonautologous Tissue Substitute, Percutaneous Endoscopic Approach |
| | | 0D194K9 | Bypass Duodenum to Duodenum with Nonautologous Tissue Substitute, Percutaneous Endoscopic Approach |
| | | 0D194KA | Bypass Duodenum to Jejunum with Nonautologous Tissue Substitute, Percutaneous Endoscopic Approach |
| | | 0D194KB | Bypass Duodenum to Ileum with Nonautologous Tissue Substitute, Percutaneous Endoscopic Approach |
| | | 0D194KL | Bypass Duodenum to Transverse Colon with Nonautol Tissue Substitute, Percutaneous Endoscopic Approach |
| | | 0D194Z4 | Bypass Duodenum to Cutaneous, Percutaneous Endoscopic Approach |
| | | 0D194Z9 | Bypass Duodenum to Duodenum, Percutaneous Endoscopic Approach |
| | | 0D194ZA | Bypass Duodenum to Jejunum, Percutaneous Endoscopic Approach |
| | | 0D194ZB | Bypass Duodenum to Ileum, Percutaneous Endoscopic Approach |
| | | 0D194ZL | Bypass Duodenum to Transverse Colon, Percutaneous Endoscopic Approach |
| | | 0D1A074 | Bypass Jejunum to Cutaneous with Autologous Tissue Substitute, Open Approach |
| | | 0D1A07A | Bypass Jejunum to Jejunum with Autologous Tissue Substitute, Open Approach |
| | | 0D1A07B | Bypass Jejunum to Ileum with Autologous Tissue Substitute, Open Approach |
| | | 0D1A07H | Bypass Jejunum to Cecum with Autologous Tissue Substitute, Open Approach |
| | | 0D1A07K | Bypass Jejunum to Ascending Colon with Autologous Tissue Substitute, Open Approach |
| | | 0D1A07L | Bypass Jejunum to Transverse Colon with Autologous Tissue Substitute, Open Approach |
| | | 0D1A07M | Bypass Jejunum to Descending Colon with Autologous Tissue Substitute, Open Approach |
| | | 0D1A07N | Bypass Jejunum to Sigmoid Colon with Autologous Tissue Substitute, Open Approach |
| | | 0D1A07P | Bypass Jejunum to Rectum with Autologous Tissue Substitute, Open Approach |

© 2019 Lewis KH et al. *JAMA Network Open*.

| | | | |
|---|---|---|---|
| OTHER ABDOMINAL OPERATION | ICD-10 Procedure | 0D1A0J4 | Bypass Jejunum to Cutaneous with Synthetic Substitute, Open Approach |
| | | 0D1A0JA | Bypass Jejunum to Jejunum with Synthetic Substitute, Open Approach |
| | | 0D1A0JB | Bypass Jejunum to Ileum with Synthetic Substitute, Open Approach |
| | | 0D1A0JH | Bypass Jejunum to Cecum with Synthetic Substitute, Open Approach |
| | | 0D1A0JK | Bypass Jejunum to Ascending Colon with Synthetic Substitute, Open Approach |
| | | 0D1A0JL | Bypass Jejunum to Transverse Colon with Synthetic Substitute, Open Approach |
| | | 0D1A0JM | Bypass Jejunum to Descending Colon with Synthetic Substitute, Open Approach |
| | | 0D1A0JN | Bypass Jejunum to Sigmoid Colon with Synthetic Substitute, Open Approach |
| | | 0D1A0JP | Bypass Jejunum to Rectum with Synthetic Substitute, Open Approach |
| | | 0D1A0K4 | Bypass Jejunum to Cutaneous with Nonautologous Tissue Substitute, Open Approach |
| | | 0D1A0KA | Bypass Jejunum to Jejunum with Nonautologous Tissue Substitute, Open Approach |
| | | 0D1A0KB | Bypass Jejunum to Ileum with Nonautologous Tissue Substitute, Open Approach |
| | | 0D1A0KH | Bypass Jejunum to Cecum with Nonautologous Tissue Substitute, Open Approach |
| | | 0D1A0KK | Bypass Jejunum to Ascending Colon with Nonautologous Tissue Substitute, Open Approach |
| | | 0D1A0KL | Bypass Jejunum to Transverse Colon with Nonautologous Tissue Substitute, Open Approach |
| | | 0D1A0KM | Bypass Jejunum to Descending Colon with Nonautologous Tissue Substitute, Open Approach |
| | | 0D1A0KN | Bypass Jejunum to Sigmoid Colon with Nonautologous Tissue Substitute, Open Approach |
| | | 0D1A0KP | Bypass Jejunum to Rectum with Nonautologous Tissue Substitute, Open Approach |
| | | 0D1A0Z4 | Bypass Jejunum to Cutaneous, Open Approach |
| | | 0D1A0ZA | Bypass Jejunum to Jejunum, Open Approach |
| | | 0D1A0ZB | Bypass Jejunum to Ileum, Open Approach |
| | | 0D1A0ZH | Bypass Jejunum to Cecum, Open Approach |
| | | 0D1A0ZK | Bypass Jejunum to Ascending Colon, Open Approach |
| | | 0D1A0ZL | Bypass Jejunum to Transverse Colon, Open Approach |
| | | 0D1A0ZM | Bypass Jejunum to Descending Colon, Open Approach |
| | | 0D1A0ZN | Bypass Jejunum to Sigmoid Colon, Open Approach |
| | | 0D1A0ZP | Bypass Jejunum to Rectum, Open Approach |
| | | 0D1A3J4 | Bypass Jejunum to Cutaneous with Synthetic Substitute, Percutaneous Approach |
| | | 0D1A474 | Bypass Jejunum to Cutaneous with Autologous Tissue Substitute, Percutaneous Endoscopic Approach |
| | | 0D1A47A | Bypass Jejunum to Jejunum with Autologous Tissue Substitute, Percutaneous Endoscopic Approach |
| | | 0D1A47B | Bypass Jejunum to Ileum with Autologous Tissue Substitute, Percutaneous Endoscopic Approach |
| | | 0D1A47H | Bypass Jejunum to Cecum with Autologous Tissue Substitute, Percutaneous Endoscopic Approach |
| | | 0D1A47K | Bypass Jejunum to Ascending Colon with Autologous Tissue Substitute, Percutaneous Endoscopic Approach |
| | | 0D1A47L | Bypass Jejunum to Transverse Colon with Autologous Tissue Substitute, Percutaneous Endoscopic Approach |
| | | 0D1A47M | Bypass Jejunum to Descending Colon with Autologous Tissue Substitute, Percutaneous Endoscopic Approach |
| | | 0D1A47N | Bypass Jejunum to Sigmoid Colon with Autologous Tissue Substitute, Percutaneous Endoscopic Approach |
| | | 0D1A47P | Bypass Jejunum to Rectum with Autologous Tissue Substitute, Percutaneous Endoscopic Approach |
| | | 0D1A4J4 | Bypass Jejunum to Cutaneous with Synthetic Substitute, Percutaneous Endoscopic Approach |
| | | 0D1A4JA | Bypass Jejunum to Jejunum with Synthetic Substitute, Percutaneous Endoscopic Approach |
| | | 0D1A4JB | Bypass Jejunum to Ileum with Synthetic Substitute, Percutaneous Endoscopic Approach |
| | | 0D1A4JH | Bypass Jejunum to Cecum with Synthetic Substitute, Percutaneous Endoscopic Approach |
| | | 0D1A4JK | Bypass Jejunum to Ascending Colon with Synthetic Substitute, Percutaneous Endoscopic Approach |
| | | 0D1A4JL | Bypass Jejunum to Transverse Colon with Synthetic Substitute, Percutaneous Endoscopic Approach |
| | | 0D1A4JM | Bypass Jejunum to Descending Colon with Synthetic Substitute, Percutaneous Endoscopic Approach |
| | | 0D1A4JN | Bypass Jejunum to Sigmoid Colon with Synthetic Substitute, Percutaneous Endoscopic Approach |
| | | 0D1A4JP | Bypass Jejunum to Rectum with Synthetic Substitute, Percutaneous Endoscopic Approach |
| | | 0D1A4K4 | Bypass Jejunum to Cutaneous with Nonautologous Tissue Substitute, Percutaneous Endoscopic Approach |
| | | 0D1A4KA | Bypass Jejunum to Jejunum with Nonautologous Tissue Substitute, Percutaneous Endoscopic Approach |
| | | 0D1A4KB | Bypass Jejunum to Ileum with Nonautologous Tissue Substitute, Percutaneous Endoscopic Approach |
| | | 0D1A4KH | Bypass Jejunum to Cecum with Nonautologous Tissue Substitute, Percutaneous Endoscopic Approach |
| | | 0D1A4KK | Bypass Jejunum to Ascending Colon with Nonautol Tissue Substitute, Percutaneous Endoscopic Approach |
| | | 0D1A4KL | Bypass Jejunum to Transverse Colon with Nonautol Tissue Substitute, Percutaneous Endoscopic Approach |
| | | 0D1A4KM | Bypass Jejunum to Descending Colon with Nonautol Tissue Substitute, Percutaneous Endoscopic Approach |
| | | 0D1A4KN | Bypass Jejunum to Sigmoid Colon with Nonautologous Tissue Substitute, Percutaneous Endoscopic Approach |
| | | 0D1A4KP | Bypass Jejunum to Rectum with Nonautologous Tissue Substitute, Percutaneous Endoscopic Approach |
| | | 0D1A4Z4 | Bypass Jejunum to Cutaneous, Percutaneous Endoscopic Approach |
| | | 0D1A4ZA | Bypass Jejunum to Jejunum, Percutaneous Endoscopic Approach |
| | | 0D1A4ZB | Bypass Jejunum to Ileum, Percutaneous Endoscopic Approach |
| | | 0D1A4ZH | Bypass Jejunum to Cecum, Percutaneous Endoscopic Approach |
| | | 0D1A4ZK | Bypass Jejunum to Ascending Colon, Percutaneous Endoscopic Approach |
| | | 0D1A4ZL | Bypass Jejunum to Transverse Colon, Percutaneous Endoscopic Approach |
| | | 0D1A4ZM | Bypass Jejunum to Descending Colon, Percutaneous Endoscopic Approach |
| | | 0D1A4ZN | Bypass Jejunum to Sigmoid Colon, Percutaneous Endoscopic Approach |
| | | 0D1A4ZP | Bypass Jejunum to Rectum, Percutaneous Endoscopic Approach |
| | | 0D1B07B | Bypass Ileum to Ileum with Autologous Tissue Substitute, Open Approach |
| | | 0D1B07H | Bypass Ileum to Cecum with Autologous Tissue Substitute, Open Approach |
| | | 0D1B07K | Bypass Ileum to Ascending Colon with Autologous Tissue Substitute, Open Approach |
| | | 0D1B07L | Bypass Ileum to Transverse Colon with Autologous Tissue Substitute, Open Approach |
| | | 0D1B07M | Bypass Ileum to Descending Colon with Autologous Tissue Substitute, Open Approach |
| | | 0D1B07N | Bypass Ileum to Sigmoid Colon with Autologous Tissue Substitute, Open Approach |
| | | 0D1B07P | Bypass Ileum to Rectum with Autologous Tissue Substitute, Open Approach |
| | | 0D1B0JB | Bypass Ileum to Ileum with Synthetic Substitute, Open Approach |
| | | 0D1B0JH | Bypass Ileum to Cecum with Synthetic Substitute, Open Approach |
| | | 0D1B0JK | Bypass Ileum to Ascending Colon with Synthetic Substitute, Open Approach |
| | | 0D1B0JL | Bypass Ileum to Transverse Colon with Synthetic Substitute, Open Approach |
| | | 0D1B0JM | Bypass Ileum to Descending Colon with Synthetic Substitute, Open Approach |
| | | 0D1B0JN | Bypass Ileum to Sigmoid Colon with Synthetic Substitute, Open Approach |
| | | 0D1B0JP | Bypass Ileum to Rectum with Synthetic Substitute, Open Approach |
| | | 0D1B0KB | Bypass Ileum to Ileum with Nonautologous Tissue Substitute, Open Approach |
| | | 0D1B0KH | Bypass Ileum to Cecum with Nonautologous Tissue Substitute, Open Approach |

© 2019 Lewis KH et al. *JAMA Network Open*.

| | | | |
|---|---|---|---|
| OTHER ABDOMINAL OPERATION | ICD-10 Procedure | 0D1B0KK | Bypass Ileum to Ascending Colon with Nonautologous Tissue Substitute, Open Approach |
| | | 0D1B0KL | Bypass Ileum to Transverse Colon with Nonautologous Tissue Substitute, Open Approach |
| | | 0D1B0KM | Bypass Ileum to Descending Colon with Nonautologous Tissue Substitute, Open Approach |
| | | 0D1B0KN | Bypass Ileum to Sigmoid Colon with Nonautologous Tissue Substitute, Open Approach |
| | | 0D1B0KP | Bypass Ileum to Rectum with Nonautologous Tissue Substitute, Open Approach |
| | | 0D1B0ZB | Bypass Ileum to Ileum, Open Approach |
| | | 0D1B0ZH | Bypass Ileum to Cecum, Open Approach |
| | | 0D1B0ZK | Bypass Ileum to Ascending Colon, Open Approach |
| | | 0D1B0ZL | Bypass Ileum to Transverse Colon, Open Approach |
| | | 0D1B0ZM | Bypass Ileum to Descending Colon, Open Approach |
| | | 0D1B0ZN | Bypass Ileum to Sigmoid Colon, Open Approach |
| | | 0D1B0ZP | Bypass Ileum to Rectum, Open Approach |
| | | 0D1B47B | Bypass Ileum to Ileum with Autologous Tissue Substitute, Percutaneous Endoscopic Approach |
| | | 0D1B47H | Bypass Ileum to Cecum with Autologous Tissue Substitute, Percutaneous Endoscopic Approach |
| | | 0D1B47K | Bypass Ileum to Ascending Colon with Autologous Tissue Substitute, Percutaneous Endoscopic Approach |
| | | 0D1B47L | Bypass Ileum to Transverse Colon with Autologous Tissue Substitute, Percutaneous Endoscopic Approach |
| | | 0D1B47M | Bypass Ileum to Descending Colon with Autologous Tissue Substitute, Percutaneous Endoscopic Approach |
| | | 0D1B47N | Bypass Ileum to Sigmoid Colon with Autologous Tissue Substitute, Percutaneous Endoscopic Approach |
| | | 0D1B47P | Bypass Ileum to Rectum with Autologous Tissue Substitute, Percutaneous Endoscopic Approach |
| | | 0D1B4JB | Bypass Ileum to Ileum with Synthetic Substitute, Percutaneous Endoscopic Approach |
| | | 0D1B4JH | Bypass Ileum to Cecum with Synthetic Substitute, Percutaneous Endoscopic Approach |
| | | 0D1B4JK | Bypass Ileum to Ascending Colon with Synthetic Substitute, Percutaneous Endoscopic Approach |
| | | 0D1B4JL | Bypass Ileum to Transverse Colon with Synthetic Substitute, Percutaneous Endoscopic Approach |
| | | 0D1B4JM | Bypass Ileum to Descending Colon with Synthetic Substitute, Percutaneous Endoscopic Approach |
| | | 0D1B4JN | Bypass Ileum to Sigmoid Colon with Synthetic Substitute, Percutaneous Endoscopic Approach |
| | | 0D1B4JP | Bypass Ileum to Rectum with Synthetic Substitute, Percutaneous Endoscopic Approach |
| | | 0D1B4KB | Bypass Ileum to Ileum with Nonautologous Tissue Substitute, Percutaneous Endoscopic Approach |
| | | 0D1B4KH | Bypass Ileum to Cecum with Nonautologous Tissue Substitute, Percutaneous Endoscopic Approach |
| | | 0D1B4KK | Bypass Ileum to Ascending Colon with Nonautologous Tissue Substitute, Percutaneous Endoscopic Approach |
| | | 0D1B4KL | Bypass Ileum to Transverse Colon with Nonautologous Tissue Substitute, Percutaneous Endoscopic Approach |
| | | 0D1B4KM | Bypass Ileum to Descending Colon with Nonautologous Tissue Substitute, Percutaneous Endoscopic Approach |
| | | 0D1B4KN | Bypass Ileum to Sigmoid Colon with Nonautologous Tissue Substitute, Percutaneous Endoscopic Approach |
| | | 0D1B4KP | Bypass Ileum to Rectum with Nonautologous Tissue Substitute, Percutaneous Endoscopic Approach |
| | | 0D1B4ZB | Bypass Ileum to Ileum, Percutaneous Endoscopic Approach |
| | | 0D1B4ZH | Bypass Ileum to Cecum, Percutaneous Endoscopic Approach |
| | | 0D1B4ZK | Bypass Ileum to Ascending Colon, Percutaneous Endoscopic Approach |
| | | 0D1B4ZL | Bypass Ileum to Transverse Colon, Percutaneous Endoscopic Approach |
| | | 0D1B4ZM | Bypass Ileum to Descending Colon, Percutaneous Endoscopic Approach |
| | | 0D1B4ZN | Bypass Ileum to Sigmoid Colon, Percutaneous Endoscopic Approach |
| | | 0D1B4ZP | Bypass Ileum to Rectum, Percutaneous Endoscopic Approach |
| | | 0D564ZZ | Destruction of Stomach, Percutaneous Endoscopic Approach |
| | | 0D574ZZ | Destruction of Stomach, Pylorus, Percutaneous Endoscopic Approach |
| | | 0D580ZZ | Destruction of Small Intestine, Open Approach |
| | | 0D584ZZ | Destruction of Small Intestine, Percutaneous Endoscopic Approach |
| | | 0D587ZZ | Destruction of Small Intestine, Via Natural or Artificial Opening |
| | | 0D5A0ZZ | Destruction of Jejunum, Open Approach |
| | | 0D5A4ZZ | Destruction of Jejunum, Percutaneous Endoscopic Approach |
| | | 0D5A7ZZ | Destruction of Jejunum, Via Natural or Artificial Opening |
| | | 0D5B0ZZ | Destruction of Ileum, Open Approach |
| | | 0D5B4ZZ | Destruction of Ileum, Percutaneous Endoscopic Approach |
| | | 0D5B7ZZ | Destruction of Ileum, Via Natural or Artificial Opening |
| | | 0D5C0ZZ | Destruction of Ileocecal Valve, Open Approach |
| | | 0D5C4ZZ | Destruction of Ileocecal Valve, Percutaneous Endoscopic Approach |
| | | 0D5C7ZZ | Destruction of Ileocecal Valve, Via Natural or Artificial Opening |
| | | 0D5U0ZZ | Destruction of Omentum, Open Approach |
| | | 0D5U4ZZ | Destruction of Omentum, Percutaneous Endoscopic Approach |
| | | 0D5V0ZZ | Destruction of Mesentery, Open Approach |
| | | 0D5V4ZZ | Destruction of Mesentery, Percutaneous Endoscopic Approach |
| | | 0D5W0ZZ | Destruction of Peritoneum, Open Approach |
| | | 0D5W4ZZ | Destruction of Peritoneum, Percutaneous Endoscopic Approach |
| | | 0D760DZ | Dilation of Stomach with Intraluminal Device, Open Approach |
| | | 0D760ZZ | Dilation of Stomach, Open Approach |
| | | 0D764DZ | Dilation of Stomach with Intraluminal Device, Percutaneous Endoscopic Approach |
| | | 0D764ZZ | Dilation of Stomach, Percutaneous Endoscopic Approach |
| | | 0D767DZ | Dilation of Stomach with Intraluminal Device, Via Natural or Artificial Opening |
| | | 0D767ZZ | Dilation of Stomach, Via Natural or Artificial Opening |
| | | 0D770DZ | Dilation of Stomach, Pylorus with Intraluminal Device, Open Approach |
| | | 0D774DZ | Dilation of Stomach, Pylorus with Intraluminal Device, Percutaneous Endoscopic Approach |
| | | 0D777DZ | Dilation of Stomach, Pylorus with Intraluminal Device, Via Natural or Artificial Opening |
| | | 0D777ZZ | Dilation of Stomach, Pylorus, Via Natural or Artificial Opening |
| | | 0D787ZZ | Dilation of Small Intestine, Via Natural or Artificial Opening |
| | | 0D797ZZ | Dilation of Duodenum, Via Natural or Artificial Opening |
| | | 0D7A7ZZ | Dilation of Jejunum, Via Natural or Artificial Opening |
| | | 0D7B7ZZ | Dilation of Ileum, Via Natural or Artificial Opening |
| | | 0D7C7ZZ | Dilation of Ileocecal Valve, Via Natural or Artificial Opening |
| | | 0D910ZX | Drainage of Upper Esophagus, Open Approach, Diagnostic |
| | | 0D914ZX | Drainage of Upper Esophagus, Percutaneous Endoscopic Approach, Diagnostic |
| | | 0D917ZX | Drainage of Upper Esophagus, Via Natural or Artificial Opening, Diagnostic |
| | | 0D920ZX | Drainage of Middle Esophagus, Open Approach, Diagnostic |
| | | 0D924ZX | Drainage of Middle Esophagus, Percutaneous Endoscopic Approach, Diagnostic |

© 2019 Lewis KH et al. *JAMA Network Open*.

| | | | |
|---|---|---|---|
| OTHER ABDOMINAL OPERATION | ICD-10 Procedure | 0D927ZX | Drainage of Middle Esophagus, Via Natural or Artificial Opening, Diagnostic |
| | | 0D930ZX | Drainage of Lower Esophagus, Open Approach, Diagnostic |
| | | 0D934ZX | Drainage of Lower Esophagus, Percutaneous Endoscopic Approach, Diagnostic |
| | | 0D937ZX | Drainage of Lower Esophagus, Via Natural or Artificial Opening, Diagnostic |
| | | 0D940ZX | Drainage of Esophagogastric Junction, Open Approach, Diagnostic |
| | | 0D944ZX | Drainage of Esophagogastric Junction, Percutaneous Endoscopic Approach, Diagnostic |
| | | 0D947ZX | Drainage of Esophagogastric Junction, Via Natural or Artificial Opening, Diagnostic |
| | | 0D950ZX | Drainage of Esophagus, Open Approach, Diagnostic |
| | | 0D954ZX | Drainage of Esophagus, Percutaneous Endoscopic Approach, Diagnostic |
| | | 0D957ZX | Drainage of Esophagus, Via Natural or Artificial Opening, Diagnostic |
| | | 0D9600Z | Drainage of Stomach with Drainage Device, Open Approach |
| | | 0D960ZZ | Drainage of Stomach, Open Approach |
| | | 0D9640Z | Drainage of Stomach with Drainage Device, Percutaneous Endoscopic Approach |
| | | 0D964ZZ | Drainage of Stomach, Percutaneous Endoscopic Approach |
| | | 0D967ZZ | Drainage of Stomach, Via Natural or Artificial Opening |
| | | 0D9800Z | Drainage of Small Intestine with Drainage Device, Open Approach |
| | | 0D980ZZ | Drainage of Small Intestine, Open Approach |
| | | 0D9840Z | Drainage of Small Intestine with Drainage Device, Percutaneous Endoscopic Approach |
| | | 0D984ZX | Drainage of Small Intestine, Percutaneous Endoscopic Approach, Diagnostic |
| | | 0D984ZZ | Drainage of Small Intestine, Percutaneous Endoscopic Approach |
| | | 0D987ZX | Drainage of Small Intestine, Via Natural or Artificial Opening, Diagnostic |
| | | 0D987ZZ | Drainage of Small Intestine, Via Natural or Artificial Opening |
| | | 0D994ZX | Drainage of Duodenum, Percutaneous Endoscopic Approach, Diagnostic |
| | | 0D997ZX | Drainage of Duodenum, Via Natural or Artificial Opening, Diagnostic |
| | | 0D9A00Z | Drainage of Jejunum with Drainage Device, Open Approach |
| | | 0D9A0ZZ | Drainage of Jejunum, Open Approach |
| | | 0D9A3ZX | Drainage of Jejunum, Percutaneous Approach, Diagnostic |
| | | 0D9A40Z | DRN JEJUN DRN DEVICE PERQ ENDO APPROACH |
| | | 0D9A4ZX | Drainage of Jejunum, Percutaneous Endoscopic Approach, Diagnostic |
| | | 0D9A4ZZ | Drainage of Jejunum, Percutaneous Endoscopic Approach |
| | | 0D9A7ZX | Drainage of Jejunum, Via Natural or Artificial Opening, Diagnostic |
| | | 0D9A7ZZ | Drainage of Jejunum, Via Natural or Artificial Opening |
| | | 0D9B00Z | Drainage of Ileum with Drainage Device, Open Approach |
| | | 0D9B0ZZ | Drainage of Ileum, Open Approach |
| | | 0D9B3ZX | Drainage of Ileum, Percutaneous Approach, Diagnostic |
| | | 0D9B40Z | Drainage of Ileum with Drainage Device, Percutaneous Endoscopic Approach |
| | | 0D9B4ZX | Drainage of Ileum, Percutaneous Endoscopic Approach, Diagnostic |
| | | 0D9B4ZZ | Drainage of Ileum, Percutaneous Endoscopic Approach |
| | | 0D9B7ZX | Drainage of Ileum, Via Natural or Artificial Opening, Diagnostic |
| | | 0D9B7ZZ | Drainage of Ileum, Via Natural or Artificial Opening |
| | | 0D9C00Z | Drainage of Ileocecal Valve with Drainage Device, Open Approach |
| | | 0D9C0ZZ | Drainage of Ileocecal Valve, Open Approach |
| | | 0D9C40Z | Drainage of Ileocecal Valve with Drainage Device, Percutaneous Endoscopic Approach |
| | | 0D9C4ZX | Drainage of Ileocecal Valve, Percutaneous Endoscopic Approach, Diagnostic |
| | | 0D9C4ZZ | Drainage of Ileocecal Valve, Percutaneous Endoscopic Approach |
| | | 0D9C70Z | Drainage of Ileocecal Valve with Drainage Device, Via Natural or Artificial Opening |
| | | 0D9C7ZX | Drainage of Ileocecal Valve, Via Natural or Artificial Opening, Diagnostic |
| | | 0D9C7ZZ | Drainage of Ileocecal Valve, Via Natural or Artificial Opening |
| | | 0D9U00Z | Drainage of Omentum with Drainage Device, Open Approach |
| | | 0D9U0ZZ | Drainage of Omentum, Open Approach |
| | | 0D9V00Z | Drainage of Mesentery with Drainage Device, Open Approach |
| | | 0D9V0ZZ | Drainage of Mesentery, Open Approach |
| | | 0D9W00Z | Drainage of Peritoneum with Drainage Device, Open Approach |
| | | 0D9W0ZZ | Drainage of Peritoneum, Open Approach |
| | | 0DB10ZX | Excision of Upper Esophagus, Open Approach, Diagnostic |
| | | 0DB13ZX | Excision of Upper Esophagus, Percutaneous Approach, Diagnostic |
| | | 0DB14ZX | Excision of Upper Esophagus, Percutaneous Endoscopic Approach, Diagnostic |
| | | 0DB17ZX | Excision of Upper Esophagus, Via Natural or Artificial Opening, Diagnostic |
| | | 0DB20ZX | Excision of Middle Esophagus, Open Approach, Diagnostic |
| | | 0DB24ZX | Excision of Middle Esophagus, Percutaneous Endoscopic Approach, Diagnostic |
| | | 0DB27ZX | Excision of Middle Esophagus, Via Natural or Artificial Opening, Diagnostic |
| | | 0DB30ZX | Excision of Lower Esophagus, Open Approach, Diagnostic |
| | | 0DB34ZX | Excision of Lower Esophagus, Percutaneous Endoscopic Approach, Diagnostic |
| | | 0DB37ZX | Excision of Lower Esophagus, Via Natural or Artificial Opening, Diagnostic |
| | | 0DB50ZX | Excision of Esophagus, Open Approach, Diagnostic |
| | | 0DB57ZX | Excision of Esophagus, Via Natural or Artificial Opening, Diagnostic |
| | | 0DB80ZZ | Excision of Small Intestine, Open Approach |
| | | 0DB84ZX | Excision of Small Intestine, Percutaneous Endoscopic Approach, Diagnostic |
| | | 0DB84ZZ | Excision of Small Intestine, Percutaneous Endoscopic Approach |
| | | 0DB87ZX | Excision of Small Intestine, Via Natural or Artificial Opening, Diagnostic |
| | | 0DB87ZZ | Excision of Small Intestine, Via Natural or Artificial Opening |
| | | 0DB88ZZ | Excision of Small Intestine, Via Natural or Artificial Opening Endoscopic |
| | | 0DB90ZZ | Excision of Duodenum, Open Approach |
| | | 0DB93ZX | Excision of Duodenum, Percutaneous Approach, Diagnostic |
| | | 0DB94ZX | Excision of Duodenum, Percutaneous Endoscopic Approach, Diagnostic |
| | | 0DB97ZX | Excision of Duodenum, Via Natural or Artificial Opening, Diagnostic |
| | | 0DBA0ZZ | Excision of Jejunum, Open Approach |
| | | 0DBA4ZX | Excision of Jejunum, Percutaneous Endoscopic Approach, Diagnostic |
| | | 0DBA4ZZ | Excision of Jejunum, Percutaneous Endoscopic Approach |
| | | 0DBA7ZX | Excision of Jejunum, Via Natural or Artificial Opening, Diagnostic |

© 2019 Lewis KH et al. *JAMA Network Open*.

| | | | |
|---|---|---|---|
| OTHER ABDOMINAL OPERATION | ICD-10 Procedure | 0DBA7ZZ | Excision of Jejunum, Via Natural or Artificial Opening |
| | | 0DBB0ZZ | EXCISION ILEUM OPEN APPROACH |
| | | 0DBB4ZX | Excision of Ileum, Percutaneous Endoscopic Approach, Diagnostic |
| | | 0DBB4ZZ | Excision of Ileum, Percutaneous Endoscopic Approach |
| | | 0DBB7ZX | Excision of Ileum, Via Natural or Artificial Opening, Diagnostic |
| | | 0DBB7ZZ | Excision of Ileum, Via Natural or Artificial Opening |
| | | 0DBC0ZZ | Excision of Ileocecal Valve, Open Approach |
| | | 0DBC4ZZ | Excision of Ileocecal Valve, Percutaneous Endoscopic Approach |
| | | 0DBC7ZZ | Excision of Ileocecal Valve, Via Natural or Artificial Opening |
| | | 0DBS4ZZ | EXC GT OMENTUM PERQ ENDO APPROACH |
| | | 0DBU0ZZ | Excision of Omentum, Open Approach |
| | | 0DBU4ZZ | Excision of Omentum, Percutaneous Endoscopic Approach |
| | | 0DBV0ZZ | Excision of Mesentery, Open Approach |
| | | 0DBV4ZZ | Excision of Mesentery, Percutaneous Endoscopic Approach |
| | | 0DBW0ZZ | Excision of Peritoneum, Open Approach |
| | | 0DBW4ZZ | Excision of Peritoneum, Percutaneous Endoscopic Approach |
| | | 0DC60ZZ | Extirpation of Matter from Stomach, Open Approach |
| | | 0DC64ZZ | Extirpation of Matter from Stomach, Percutaneous Endoscopic Approach |
| | | 0DC80ZZ | Extirpation of Matter from Small Intestine, Open Approach |
| | | 0DC84ZZ | Extirpation of Matter from Small Intestine, Percutaneous Endoscopic Approach |
| | | 0DCA0ZZ | Extirpation of Matter from Jejunum, Open Approach |
| | | 0DCA4ZZ | Extirpation of Matter from Jejunum, Percutaneous Endoscopic Approach |
| | | 0DCB0ZZ | Extirpation of Matter from Ileum, Open Approach |
| | | 0DCB4ZZ | Extirpation of Matter from Ileum, Percutaneous Endoscopic Approach |
| | | 0DCC0ZZ | Extirpation of Matter from Ileocecal Valve, Open Approach |
| | | 0DCC4ZZ | Extirpation of Matter from Ileocecal Valve, Percutaneous Endoscopic Approach |
| | | 0DD14ZX | Extraction of Upper Esophagus, Percutaneous Endoscopic Approach, Diagnostic |
| | | 0DD24ZX | Extraction of Middle Esophagus, Percutaneous Endoscopic Approach, Diagnostic |
| | | 0DD34ZX | Extraction of Lower Esophagus, Percutaneous Endoscopic Approach, Diagnostic |
| | | 0DD44ZX | Extraction of Esophagogastric Junction, Percutaneous Endoscopic Approach, Diagnostic |
| | | 0DD54ZX | Extraction of Esophagus, Percutaneous Endoscopic Approach, Diagnostic |
| | | 0DD84ZX | Extraction of Small Intestine, Percutaneous Endoscopic Approach, Diagnostic |
| | | 0DD94ZX | Extraction of Duodenum, Percutaneous Endoscopic Approach, Diagnostic |
| | | 0DDA4ZX | Extraction of Jejunum, Percutaneous Endoscopic Approach, Diagnostic |
| | | 0DDB4ZX | Extraction of Ileum, Percutaneous Endoscopic Approach, Diagnostic |
| | | 0DDC4ZX | Extraction of Ileocecal Valve, Percutaneous Endoscopic Approach, Diagnostic |
| | | 0DF60ZZ | Fragmentation in Stomach, Open Approach |
| | | 0DF64ZZ | Fragmentation in Stomach, Percutaneous Endoscopic Approach |
| | | 0DF67ZZ | Fragmentation in Stomach, Via Natural or Artificial Opening |
| | | 0DF80ZZ | Fragmentation in Small Intestine, Open Approach |
| | | 0DF84ZZ | Fragmentation in Small Intestine, Percutaneous Endoscopic Approach |
| | | 0DF87ZZ | Fragmentation in Small Intestine, Via Natural or Artificial Opening |
| | | 0DF90ZZ | Fragmentation in Duodenum, Open Approach |
| | | 0DF94ZZ | Fragmentation in Duodenum, Percutaneous Endoscopic Approach |
| | | 0DF97ZZ | Fragmentation in Duodenum, Via Natural or Artificial Opening |
| | | 0DFA0ZZ | Fragmentation in Jejunum, Open Approach |
| | | 0DFA4ZZ | Fragmentation in Jejunum, Percutaneous Endoscopic Approach |
| | | 0DFA7ZZ | Fragmentation in Jejunum, Via Natural or Artificial Opening |
| | | 0DFB0ZZ | Fragmentation in Ileum, Open Approach |
| | | 0DFB4ZZ | Fragmentation in Ileum, Percutaneous Endoscopic Approach |
| | | 0DFB7ZZ | Fragmentation in Ileum, Via Natural or Artificial Opening |
| | | 0DH00YZ | Insertion of Other Device into Upper Intestinal Tract, Open Approach |
| | | 0DH04YZ | Insertion of Other Device into Upper Intestinal Tract, Percutaneous Endoscopic Approach |
| | | 0DH50DZ | Insertion of Intraluminal Device into Esophagus, Open Approach |
| | | 0DH54DZ | Insertion of Intraluminal Device into Esophagus, Percutaneous Endoscopic Approach |
| | | 0DH57DZ | Insertion of Intraluminal Device into Esophagus, Via Natural or Artificial Opening |
| | | 0DH602Z | Insertion of Monitoring Device into Stomach, Open Approach |
| | | 0DH60DZ | Insertion of Intraluminal Device into Stomach, Open Approach |
| | | 0DH60YZ | Insertion of Other Device into Stomach, Open Approach |
| | | 0DH642Z | Insertion of Monitoring Device into Stomach, Percutaneous Endoscopic Approach |
| | | 0DH64DZ | Insertion of Intraluminal Device into Stomach, Percutaneous Endoscopic Approach |
| | | 0DH64YZ | Insertion of Other Device into Stomach, Percutaneous Endoscopic Approach |
| | | 0DH67DZ | Insertion of Intraluminal Device into Stomach, Via Natural or Artificial Opening |
| | | 0DH802Z | Insertion of Monitoring Device into Small Intestine, Open Approach |
| | | 0DH842Z | Insertion of Monitoring Device into Small Intestine, Percutaneous Endoscopic Approach |
| | | 0DHA02Z | Insertion of Monitoring Device into Jejunum, Open Approach |
| | | 0DHA42Z | Insertion of Monitoring Device into Jejunum, Percutaneous Endoscopic Approach |
| | | 0DHB02Z | Insertion of Monitoring Device into Ileum, Open Approach |
| | | 0DHB42Z | Insertion of Monitoring Device into Ileum, Percutaneous Endoscopic Approach |
| | | 0DJ00ZZ | Inspection of Upper Intestinal Tract, Open Approach |
| | | 0DJ07ZZ | Inspection of Upper Intestinal Tract, Via Natural or Artificial Opening |
| | | 0DJ60ZZ | Inspection of Stomach, Open Approach |
| | | 0DJU0ZZ | Inspection of Omentum, Open Approach |
| | | 0DJU4ZZ | Inspection of Omentum, Percutaneous Endoscopic Approach |
| | | 0DJV0ZZ | Inspection of Mesentery, Open Approach |
| | | 0DJV4ZZ | Inspection of Mesentery, Percutaneous Endoscopic Approach |
| | | 0DJW0ZZ | Inspection of Peritoneum, Open Approach |
| | | 0DJW4ZZ | Inspection of Peritoneum, Percutaneous Endoscopic Approach |
| | | 0DL60CZ | Occlusion of Stomach with Extraluminal Device, Open Approach |
| | | 0DL60DZ | Occlusion of Stomach with Intraluminal Device, Open Approach |

© 2019 Lewis KH et al. *JAMA Network Open*.

| | | | |
|---|---|---|---|
| OTHER ABDOMINAL OPERATION | ICD-10 Procedure | 0DL60ZZ | Occlusion of Stomach, Open Approach |
| | | 0DL64CZ | Occlusion of Stomach with Extraluminal Device, Percutaneous Endoscopic Approach |
| | | 0DL64DZ | Occlusion of Stomach with Intraluminal Device, Percutaneous Endoscopic Approach |
| | | 0DL64ZZ | Occlusion of Stomach, Percutaneous Endoscopic Approach |
| | | 0DL67DZ | Occlusion of Stomach with Intraluminal Device, Via Natural or Artificial Opening |
| | | 0DL67ZZ | Occlusion of Stomach, Via Natural or Artificial Opening |
| | | 0DL70CZ | Occlusion of Stomach, Pylorus with Extraluminal Device, Open Approach |
| | | 0DL70DZ | Occlusion of Stomach, Pylorus with Intraluminal Device, Open Approach |
| | | 0DL70ZZ | Occlusion of Stomach, Pylorus, Open Approach |
| | | 0DL74CZ | Occlusion of Stomach, Pylorus with Extraluminal Device, Percutaneous Endoscopic Approach |
| | | 0DL74DZ | Occlusion of Stomach, Pylorus with Intraluminal Device, Percutaneous Endoscopic Approach |
| | | 0DL74ZZ | Occlusion of Stomach, Pylorus, Percutaneous Endoscopic Approach |
| | | 0DL77DZ | Occlusion of Stomach, Pylorus with Intraluminal Device, Via Natural or Artificial Opening |
| | | 0DL77ZZ | Occlusion of Stomach, Pylorus, Via Natural or Artificial Opening |
| | | 0DL80CZ | Occlusion of Small Intestine with Extraluminal Device, Open Approach |
| | | 0DL80DZ | Occlusion of Small Intestine with Intraluminal Device, Open Approach |
| | | 0DL80ZZ | Occlusion of Small Intestine, Open Approach |
| | | 0DL84CZ | Occlusion of Small Intestine with Extraluminal Device, Percutaneous Endoscopic Approach |
| | | 0DL84DZ | Occlusion of Small Intestine with Intraluminal Device, Percutaneous Endoscopic Approach |
| | | 0DL84ZZ | Occlusion of Small Intestine, Percutaneous Endoscopic Approach |
| | | 0DL87DZ | Occlusion of Small Intestine with Intraluminal Device, Via Natural or Artificial Opening |
| | | 0DL87ZZ | Occlusion of Small Intestine, Via Natural or Artificial Opening |
| | | 0DL90CZ | Occlusion of Duodenum with Extraluminal Device, Open Approach |
| | | 0DL90DZ | Occlusion of Duodenum with Intraluminal Device, Open Approach |
| | | 0DL90ZZ | Occlusion of Duodenum, Open Approach |
| | | 0DL94CZ | Occlusion of Duodenum with Extraluminal Device, Percutaneous Endoscopic Approach |
| | | 0DL94DZ | Occlusion of Duodenum with Intraluminal Device, Percutaneous Endoscopic Approach |
| | | 0DL94ZZ | Occlusion of Duodenum, Percutaneous Endoscopic Approach |
| | | 0DL97DZ | Occlusion of Duodenum with Intraluminal Device, Via Natural or Artificial Opening |
| | | 0DL97ZZ | Occlusion of Duodenum, Via Natural or Artificial Opening |
| | | 0DLA0CZ | Occlusion of Jejunum with Extraluminal Device, Open Approach |
| | | 0DLA0DZ | Occlusion of Jejunum with Intraluminal Device, Open Approach |
| | | 0DLA0ZZ | Occlusion of Jejunum, Open Approach |
| | | 0DLA4CZ | Occlusion of Jejunum with Extraluminal Device, Percutaneous Endoscopic Approach |
| | | 0DLA4DZ | Occlusion of Jejunum with Intraluminal Device, Percutaneous Endoscopic Approach |
| | | 0DLA4ZZ | Occlusion of Jejunum, Percutaneous Endoscopic Approach |
| | | 0DLA7DZ | Occlusion of Jejunum with Intraluminal Device, Via Natural or Artificial Opening |
| | | 0DLA7ZZ | Occlusion of Jejunum, Via Natural or Artificial Opening |
| | | 0DLB0CZ | Occlusion of Ileum with Extraluminal Device, Open Approach |
| | | 0DLB0DZ | Occlusion of Ileum with Intraluminal Device, Open Approach |
| | | 0DLB0ZZ | Occlusion of Ileum, Open Approach |
| | | 0DLB4CZ | Occlusion of Ileum with Extraluminal Device, Percutaneous Endoscopic Approach |
| | | 0DLB4DZ | Occlusion of Ileum with Intraluminal Device, Percutaneous Endoscopic Approach |
| | | 0DLB4ZZ | Occlusion of Ileum, Percutaneous Endoscopic Approach |
| | | 0DLB7DZ | Occlusion of Ileum with Intraluminal Device, Via Natural or Artificial Opening |
| | | 0DLB7ZZ | Occlusion of Ileum, Via Natural or Artificial Opening |
| | | 0DLC0CZ | Occlusion of Ileocecal Valve with Extraluminal Device, Open Approach |
| | | 0DLC0DZ | Occlusion of Ileocecal Valve with Intraluminal Device, Open Approach |
| | | 0DLC0ZZ | Occlusion of Ileocecal Valve, Open Approach |
| | | 0DLC4CZ | Occlusion of Ileocecal Valve with Extraluminal Device, Percutaneous Endoscopic Approach |
| | | 0DLC4DZ | Occlusion of Ileocecal Valve with Intraluminal Device, Percutaneous Endoscopic Approach |
| | | 0DLC4ZZ | Occlusion of Ileocecal Valve, Percutaneous Endoscopic Approach |
| | | 0DLC7DZ | Occlusion of Ileocecal Valve with Intraluminal Device, Via Natural or Artificial Opening |
| | | 0DLC7ZZ | Occlusion of Ileocecal Valve, Via Natural or Artificial Opening |
| | | 0DM60ZZ | Reattachment of Stomach, Open Approach |
| | | 0DM64ZZ | Reattachment of Stomach, Percutaneous Endoscopic Approach |
| | | 0DM80ZZ | Reattachment of Small Intestine, Open Approach |
| | | 0DM84ZZ | Reattachment of Small Intestine, Percutaneous Endoscopic Approach |
| | | 0DM90ZZ | Reattachment of Duodenum, Open Approach |
| | | 0DM94ZZ | Reattachment of Duodenum, Percutaneous Endoscopic Approach |
| | | 0DMA0ZZ | Reattachment of Jejunum, Open Approach |
| | | 0DMA4ZZ | Reattachment of Jejunum, Percutaneous Endoscopic Approach |
| | | 0DMB0ZZ | Reattachment of Ileum, Open Approach |
| | | 0DMB4ZZ | Reattachment of Ileum, Percutaneous Endoscopic Approach |
| | | 0DN60ZZ | Release Stomach, Open Approach |
| | | 0DN64ZZ | Release Stomach, Percutaneous Endoscopic Approach |
| | | 0DN67ZZ | Release Stomach, Via Natural or Artificial Opening |
| | | 0DN70ZZ | Release Stomach, Pylorus, Open Approach |
| | | 0DN74ZZ | Release Stomach, Pylorus, Percutaneous Endoscopic Approach |
| | | 0DN77ZZ | Release Stomach, Pylorus, Via Natural or Artificial Opening |
| | | 0DN80ZZ | RLSE SM INTESTINE OPEN APPROACH |
| | | 0DN84ZZ | Release Small Intestine, Percutaneous Endoscopic Approach |
| | | 0DN90ZZ | Release Duodenum, Open Approach |
| | | 0DN94ZZ | Release Duodenum, Percutaneous Endoscopic Approach |
| | | 0DNA0ZZ | Release Jejunum, Open Approach |
| | | 0DNA4ZZ | Release Jejunum, Percutaneous Endoscopic Approach |
| | | 0DNB0ZZ | Release Ileum, Open Approach |
| | | 0DNB4ZZ | Release Ileum, Percutaneous Endoscopic Approach |
| | | 0DNC0ZZ | Release Ileocecal Valve, Open Approach |
| | | 0DNC4ZZ | Release Ileocecal Valve, Percutaneous Endoscopic Approach |

© 2019 Lewis KH et al. *JAMA Network Open*.

| | | | |
|---|---|---|---|
| OTHER ABDOMINAL OPERATION | ICD-10 Procedure | 0DNC7ZZ | Release Ileocecal Valve, Via Natural or Artificial Opening |
| | | 0DNJ0ZZ | Release Appendix, Open Approach |
| | | 0DNJ4ZZ | Release Appendix, Percutaneous Endoscopic Approach |
| | | 0DNS4ZZ | RELEASE GT OMENTUM PERQ ENDO APPR |
| | | 0DNT4ZZ | RLSE LESSER OMENTUM PERQ ENDO APPR |
| | | 0DNU0ZZ | Release Omentum, Open Approach |
| | | 0DNU4ZZ | Release Omentum, Percutaneous Endoscopic Approach |
| | | 0DNV0ZZ | Release Mesentery, Open Approach |
| | | 0DNV4ZZ | Release Mesentery, Percutaneous Endoscopic Approach |
| | | 0DNW0ZZ | Release Peritoneum, Open Approach |
| | | 0DNW4ZZ | Release Peritoneum, Percutaneous Endoscopic Approach |
| | | 0DP000Z | Removal of Drainage Device from Upper Intestinal Tract, Open Approach |
| | | 0DP002Z | Removal of Monitoring Device from Upper Intestinal Tract, Open Approach |
| | | 0DP007Z | Removal of Autologous Tissue Substitute from Upper Intestinal Tract, Open Approach |
| | | 0DP00CZ | Removal of Extraluminal Device from Upper Intestinal Tract, Open Approach |
| | | 0DP00DZ | Removal of Intraluminal Device from Upper Intestinal Tract, Open Approach |
| | | 0DP00JZ | Removal of Synthetic Substitute from Upper Intestinal Tract, Open Approach |
| | | 0DP00KZ | Removal of Nonautologous Tissue Substitute from Upper Intestinal Tract, Open Approach |
| | | 0DP040Z | Removal of Drainage Device from Upper Intestinal Tract, Percutaneous Endoscopic Approach |
| | | 0DP042Z | Removal of Monitoring Device from Upper Intestinal Tract, Percutaneous Endoscopic Approach |
| | | 0DP047Z | Removal of Autologous Tissue Substitute from Upper Intestinal Tract, Percutaneous Endoscopic Approach |
| | | 0DP04CZ | Removal of Extraluminal Device from Upper Intestinal Tract, Percutaneous Endoscopic Approach |
| | | 0DP04DZ | Removal of Intraluminal Device from Upper Intestinal Tract, Percutaneous Endoscopic Approach |
| | | 0DP04JZ | Removal of Synthetic Substitute from Upper Intestinal Tract, Percutaneous Endoscopic Approach |
| | | 0DP04KZ | Removal of Nonautologous Tissue Substitute from Upper Intestinal Tract, Percutaneous Endoscopic Approach |
| | | 0DP077Z | Removal of Autologous Tissue Substitute from Upper Intestinal Tract, Via Natural or Artificial Opening |
| | | 0DP07CZ | Removal of Extraluminal Device from Upper Intestinal Tract, Via Natural or Artificial Opening |
| | | 0DP07JZ | Removal of Synthetic Substitute from Upper Intestinal Tract, Via Natural or Artificial Opening |
| | | 0DP07KZ | Removal of Nonautologous Tissue Substitute from Upper Intestinal Tract, Via Natural or Artificial Opening |
| | | 0DP600Z | Removal of Drainage Device from Stomach, Open Approach |
| | | 0DP602Z | Removal of Monitoring Device from Stomach, Open Approach |
| | | 0DP607Z | Removal of Autologous Tissue Substitute from Stomach, Open Approach |
| | | 0DP60CZ | Removal of Extraluminal Device from Stomach, Open Approach |
| | | 0DP60DZ | Removal of Intraluminal Device from Stomach, Open Approach |
| | | 0DP60JZ | Removal of Synthetic Substitute from Stomach, Open Approach |
| | | 0DP60KZ | Removal of Nonautologous Tissue Substitute from Stomach, Open Approach |
| | | 0DP60YZ | Removal of Other Device from Stomach, Open Approach |
| | | 0DP640Z | Removal of Drainage Device from Stomach, Percutaneous Endoscopic Approach |
| | | 0DP642Z | Removal of Monitoring Device from Stomach, Percutaneous Endoscopic Approach |
| | | 0DP647Z | Removal of Autologous Tissue Substitute from Stomach, Percutaneous Endoscopic Approach |
| | | 0DP64DZ | Removal of Intraluminal Device from Stomach, Percutaneous Endoscopic Approach |
| | | 0DP64JZ | Removal of Synthetic Substitute from Stomach, Percutaneous Endoscopic Approach |
| | | 0DP64KZ | Removal of Nonautologous Tissue Substitute from Stomach, Percutaneous Endoscopic Approach |
| | | 0DP64YZ | Removal of Other Device from Stomach, Percutaneous Endoscopic Approach |
| | | 0DP677Z | Removal of Autologous Tissue Substitute from Stomach, Via Natural or Artificial Opening |
| | | 0DP67CZ | Removal of Extraluminal Device from Stomach, Via Natural or Artificial Opening |
| | | 0DP67JZ | Removal of Synthetic Substitute from Stomach, Via Natural or Artificial Opening |
| | | 0DP67KZ | Removal of Nonautologous Tissue Substitute from Stomach, Via Natural or Artificial Opening |
| | | 0DPU00Z | Removal of Drainage Device from Omentum, Open Approach |
| | | 0DPU07Z | Removal of Autologous Tissue Substitute from Omentum, Open Approach |
| | | 0DPU0JZ | REMOVAL SYNTHETIC SUBST OMENTUM OPEN |
| | | 0DPU0KZ | Removal of Nonautologous Tissue Substitute from Omentum, Open Approach |
| | | 0DPU40Z | Removal of Drainage Device from Omentum, Percutaneous Endoscopic Approach |
| | | 0DPU47Z | Removal of Autologous Tissue Substitute from Omentum, Percutaneous Endoscopic Approach |
| | | 0DPU4JZ | Removal of Synthetic Substitute from Omentum, Percutaneous Endoscopic Approach |
| | | 0DPU4KZ | Removal of Nonautologous Tissue Substitute from Omentum, Percutaneous Endoscopic Approach |
| | | 0DPV00Z | Removal of Drainage Device from Mesentery, Open Approach |
| | | 0DPV07Z | Removal of Autologous Tissue Substitute from Mesentery, Open Approach |
| | | 0DPV0JZ | Removal of Synthetic Substitute from Mesentery, Open Approach |
| | | 0DPV0KZ | Removal of Nonautologous Tissue Substitute from Mesentery, Open Approach |
| | | 0DPV40Z | Removal of Drainage Device from Mesentery, Percutaneous Endoscopic Approach |
| | | 0DPV47Z | Removal of Autologous Tissue Substitute from Mesentery, Percutaneous Endoscopic Approach |
| | | 0DPV4JZ | Removal of Synthetic Substitute from Mesentery, Percutaneous Endoscopic Approach |
| | | 0DPV4KZ | Removal of Nonautologous Tissue Substitute from Mesentery, Percutaneous Endoscopic Approach |
| | | 0DPW00Z | Removal of Drainage Device from Peritoneum, Open Approach |
| | | 0DPW07Z | Removal of Autologous Tissue Substitute from Peritoneum, Open Approach |
| | | 0DPW0JZ | Removal of Synthetic Substitute from Peritoneum, Open Approach |
| | | 0DPW0KZ | Removal of Nonautologous Tissue Substitute from Peritoneum, Open Approach |
| | | 0DPW40Z | Removal of Drainage Device from Peritoneum, Percutaneous Endoscopic Approach |
| | | 0DPW47Z | Removal of Autologous Tissue Substitute from Peritoneum, Percutaneous Endoscopic Approach |
| | | 0DPW4JZ | Removal of Synthetic Substitute from Peritoneum, Percutaneous Endoscopic Approach |
| | | 0DPW4KZ | Removal of Nonautologous Tissue Substitute from Peritoneum, Percutaneous Endoscopic Approach |
| | | 0DQ50ZZ | Repair Esophagus, Open Approach |
| | | 0DQ54ZZ | Repair Esophagus, Percutaneous Endoscopic Approach |
| | | 0DQ57ZZ | Repair Esophagus, Via Natural or Artificial Opening |
| | | 0DQ60ZZ | REPAIR / STOMACH / OPEN APPROACH |
| | | 0DQ64ZZ | Repair Stomach, Percutaneous Endoscopic Approach |
| | | 0DQ67ZZ | Repair Stomach, Via Natural or Artificial Opening |
| | | 0DQ70ZZ | Repair Stomach, Pylorus, Open Approach |
| | | 0DQ74ZZ | Repair Stomach, Pylorus, Percutaneous Endoscopic Approach |

© 2019 Lewis KH et al. *JAMA Network Open*.

| | | | |
|---|---|---|---|
| OTHER ABDOMINAL OPERATION | ICD-10 Procedure | 0DQ77ZZ | Repair Stomach, Pylorus, Via Natural or Artificial Opening |
| | | 0DQ80ZZ | Repair Small Intestine, Open Approach |
| | | 0DQ84ZZ | Repair Small Intestine, Percutaneous Endoscopic Approach |
| | | 0DQ87ZZ | Repair Small Intestine, Via Natural or Artificial Opening |
| | | 0DQ90ZZ | Repair Duodenum, Open Approach |
| | | 0DQ94ZZ | Repair Duodenum, Percutaneous Endoscopic Approach |
| | | 0DQ97ZZ | Repair Duodenum, Via Natural or Artificial Opening |
| | | 0DQA0ZZ | Repair Jejunum, Open Approach |
| | | 0DQA4ZZ | REPAIR JEJUNUM PERQ ENDOSCOPIC APPR |
| | | 0DQA7ZZ | Repair Jejunum, Via Natural or Artificial Opening |
| | | 0DQB0ZZ | Repair Ileum, Open Approach |
| | | 0DQB4ZZ | Repair Ileum, Percutaneous Endoscopic Approach |
| | | 0DQB7ZZ | Repair Ileum, Via Natural or Artificial Opening |
| | | 0DQC0ZZ | Repair Ileocecal Valve, Open Approach |
| | | 0DQC4ZZ | Repair Ileocecal Valve, Percutaneous Endoscopic Approach |
| | | 0DQC7ZZ | Repair Ileocecal Valve, Via Natural or Artificial Opening |
| | | 0DQU0ZZ | Repair Omentum, Open Approach |
| | | 0DQU4ZZ | Repair Omentum, Percutaneous Endoscopic Approach |
| | | 0DQV0ZZ | Repair Mesentery, Open Approach |
| | | 0DQV4ZZ | Repair Mesentery, Percutaneous Endoscopic Approach |
| | | 0DRU07Z | Replacement of Omentum with Autologous Tissue Substitute, Open Approach |
| | | 0DRU0JZ | Replacement of Omentum with Synthetic Substitute, Open Approach |
| | | 0DRU0KZ | Replacement of Omentum with Nonautologous Tissue Substitute, Open Approach |
| | | 0DRU47Z | Replacement of Omentum with Autologous Tissue Substitute, Percutaneous Endoscopic Approach |
| | | 0DRU4JZ | Replacement of Omentum with Synthetic Substitute, Percutaneous Endoscopic Approach |
| | | 0DRU4KZ | Replacement of Omentum with Nonautologous Tissue Substitute, Percutaneous Endoscopic Approach |
| | | 0DRV07Z | Replacement of Mesentery with Autologous Tissue Substitute, Open Approach |
| | | 0DRV0JZ | Replacement of Mesentery with Synthetic Substitute, Open Approach |
| | | 0DRV0KZ | Replacement of Mesentery with Nonautologous Tissue Substitute, Open Approach |
| | | 0DRV47Z | Replacement of Mesentery with Autologous Tissue Substitute, Percutaneous Endoscopic Approach |
| | | 0DRV4JZ | Replacement of Mesentery with Synthetic Substitute, Percutaneous Endoscopic Approach |
| | | 0DRV4KZ | Replacement of Mesentery with Nonautologous Tissue Substitute, Percutaneous Endoscopic Approach |
| | | 0DS60ZZ | Reposition Stomach, Open Approach |
| | | 0DS64ZZ | Reposition Stomach, Percutaneous Endoscopic Approach |
| | | 0DS80ZZ | Reposition Small Intestine, Open Approach |
| | | 0DS84ZZ | Reposition Small Intestine, Percutaneous Endoscopic Approach |
| | | 0DS87ZZ | Reposition Small Intestine, Via Natural or Artificial Opening |
| | | 0DS90ZZ | Reposition Duodenum, Open Approach |
| | | 0DS94ZZ | Reposition Duodenum, Percutaneous Endoscopic Approach |
| | | 0DS97ZZ | Reposition Duodenum, Via Natural or Artificial Opening |
| | | 0DSA0ZZ | Reposition Jejunum, Open Approach |
| | | 0DSA4ZZ | Reposition Jejunum, Percutaneous Endoscopic Approach |
| | | 0DSA7ZZ | Reposition Jejunum, Via Natural or Artificial Opening |
| | | 0DSB0ZZ | Reposition Ileum, Open Approach |
| | | 0DSB4ZZ | Reposition Ileum, Percutaneous Endoscopic Approach |
| | | 0DSB7ZZ | Reposition Ileum, Via Natural or Artificial Opening |
| | | 0DT80ZZ | RESECTION SMALL INTESTINE OPEN |
| | | 0DT90ZZ | Resection of Duodenum, Open Approach |
| | | 0DT94ZZ | Resection of Duodenum, Percutaneous Endoscopic Approach |
| | | 0DT97ZZ | Resection of Duodenum, Via Natural or Artificial Opening |
| | | 0DTA0ZZ | Resection of Jejunum, Open Approach |
| | | 0DTA4ZZ | Resection of Jejunum, Percutaneous Endoscopic Approach |
| | | 0DTA7ZZ | Resection of Jejunum, Via Natural or Artificial Opening |
| | | 0DTB0ZZ | Resection of Ileum, Open Approach |
| | | 0DTB4ZZ | Resection of Ileum, Percutaneous Endoscopic Approach |
| | | 0DTB7ZZ | Resection of Ileum, Via Natural or Artificial Opening |
| | | 0DTC0ZZ | Resection of Ileocecal Valve, Open Approach |
| | | 0DTC4ZZ | Resection of Ileocecal Valve, Percutaneous Endoscopic Approach |
| | | 0DTC7ZZ | Resection of Ileocecal Valve, Via Natural or Artificial Opening |
| | | 0DTU0ZZ | Resection of Omentum, Open Approach |
| | | 0DTU4ZZ | Resection of Omentum, Percutaneous Endoscopic Approach |
| | | 0DU607Z | Supplement Stomach with Autologous Tissue Substitute, Open Approach |
| | | 0DU60JZ | Supplement Stomach with Synthetic Substitute, Open Approach |
| | | 0DU60KZ | Supplement Stomach with Nonautologous Tissue Substitute, Open Approach |
| | | 0DU647Z | Supplement Stomach with Autologous Tissue Substitute, Percutaneous Endoscopic Approach |
| | | 0DU64JZ | Supplement Stomach with Synthetic Substitute, Percutaneous Endoscopic Approach |
| | | 0DU64KZ | Supplement Stomach with Nonautologous Tissue Substitute, Percutaneous Endoscopic Approach |
| | | 0DU677Z | Supplement Stomach with Autologous Tissue Substitute, Via Natural or Artificial Opening |
| | | 0DU67JZ | Supplement Stomach with Synthetic Substitute, Via Natural or Artificial Opening |
| | | 0DU67KZ | Supplement Stomach with Nonautologous Tissue Substitute, Via Natural or Artificial Opening |
| | | 0DU707Z | Supplement Stomach, Pylorus with Autologous Tissue Substitute, Open Approach |
| | | 0DU70JZ | Supplement Stomach, Pylorus with Synthetic Substitute, Open Approach |
| | | 0DU70KZ | Supplement Stomach, Pylorus with Nonautologous Tissue Substitute, Open Approach |
| | | 0DU747Z | Supplement Stomach, Pylorus with Autologous Tissue Substitute, Percutaneous Endoscopic Approach |
| | | 0DU74JZ | Supplement Stomach, Pylorus with Synthetic Substitute, Percutaneous Endoscopic Approach |
| | | 0DU74KZ | Supplement Stomach, Pylorus with Nonautologous Tissue Substitute, Percutaneous Endoscopic Approach |
| | | 0DU777Z | Supplement Stomach, Pylorus with Autologous Tissue Substitute, Via Natural or Artificial Opening |
| | | 0DU77JZ | Supplement Stomach, Pylorus with Synthetic Substitute, Via Natural or Artificial Opening |
| | | 0DU77KZ | Supplement Stomach, Pylorus with Nonautologous Tissue Substitute, Via Natural or Artificial Opening |
| | | 0DU807Z | Supplement Small Intestine with Autologous Tissue Substitute, Open Approach |

© 2019 Lewis KH et al. *JAMA Network Open*.

| | | | |
|---|---|---|---|
| OTHER ABDOMINAL OPERATION | ICD-10 Procedure | 0DU80JZ | Supplement Small Intestine with Synthetic Substitute, Open Approach |
| | | 0DU80KZ | Supplement Small Intestine with Nonautologous Tissue Substitute, Open Approach |
| | | 0DU847Z | Supplement Small Intestine with Autologous Tissue Substitute, Percutaneous Endoscopic Approach |
| | | 0DU84JZ | Supplement Small Intestine with Synthetic Substitute, Percutaneous Endoscopic Approach |
| | | 0DU84KZ | Supplement Small Intestine with Nonautologous Tissue Substitute, Percutaneous Endoscopic Approach |
| | | 0DU877Z | Supplement Small Intestine with Autologous Tissue Substitute, Via Natural or Artificial Opening |
| | | 0DU87JZ | Supplement Small Intestine with Synthetic Substitute, Via Natural or Artificial Opening |
| | | 0DU87KZ | Supplement Small Intestine with Nonautologous Tissue Substitute, Via Natural or Artificial Opening |
| | | 0DU907Z | Supplement Duodenum with Autologous Tissue Substitute, Open Approach |
| | | 0DU90JZ | Supplement Duodenum with Synthetic Substitute, Open Approach |
| | | 0DU90KZ | Supplement Duodenum with Nonautologous Tissue Substitute, Open Approach |
| | | 0DU947Z | Supplement Duodenum with Autologous Tissue Substitute, Percutaneous Endoscopic Approach |
| | | 0DU94JZ | Supplement Duodenum with Synthetic Substitute, Percutaneous Endoscopic Approach |
| | | 0DU94KZ | Supplement Duodenum with Nonautologous Tissue Substitute, Percutaneous Endoscopic Approach |
| | | 0DU977Z | Supplement Duodenum with Autologous Tissue Substitute, Via Natural or Artificial Opening |
| | | 0DU97JZ | Supplement Duodenum with Synthetic Substitute, Via Natural or Artificial Opening |
| | | 0DU97KZ | Supplement Duodenum with Nonautologous Tissue Substitute, Via Natural or Artificial Opening |
| | | 0DUA07Z | Supplement Jejunum with Autologous Tissue Substitute, Open Approach |
| | | 0DUA0JZ | Supplement Jejunum with Synthetic Substitute, Open Approach |
| | | 0DUA0KZ | Supplement Jejunum with Nonautologous Tissue Substitute, Open Approach |
| | | 0DUA47Z | Supplement Jejunum with Autologous Tissue Substitute, Percutaneous Endoscopic Approach |
| | | 0DUA4JZ | Supplement Jejunum with Synthetic Substitute, Percutaneous Endoscopic Approach |
| | | 0DUA4KZ | Supplement Jejunum with Nonautologous Tissue Substitute, Percutaneous Endoscopic Approach |
| | | 0DUA77Z | Supplement Jejunum with Autologous Tissue Substitute, Via Natural or Artificial Opening |
| | | 0DUA7JZ | Supplement Jejunum with Synthetic Substitute, Via Natural or Artificial Opening |
| | | 0DUA7KZ | Supplement Jejunum with Nonautologous Tissue Substitute, Via Natural or Artificial Opening |
| | | 0DUB07Z | Supplement Ileum with Autologous Tissue Substitute, Open Approach |
| | | 0DUB0JZ | Supplement Ileum with Synthetic Substitute, Open Approach |
| | | 0DUB0KZ | Supplement Ileum with Nonautologous Tissue Substitute, Open Approach |
| | | 0DUB47Z | Supplement Ileum with Autologous Tissue Substitute, Percutaneous Endoscopic Approach |
| | | 0DUB4JZ | Supplement Ileum with Synthetic Substitute, Percutaneous Endoscopic Approach |
| | | 0DUB4KZ | Supplement Ileum with Nonautologous Tissue Substitute, Percutaneous Endoscopic Approach |
| | | 0DUB77Z | Supplement Ileum with Autologous Tissue Substitute, Via Natural or Artificial Opening |
| | | 0DUB7JZ | Supplement Ileum with Synthetic Substitute, Via Natural or Artificial Opening |
| | | 0DUB7KZ | Supplement Ileum with Nonautologous Tissue Substitute, Via Natural or Artificial Opening |
| | | 0DUC07Z | Supplement Ileocecal Valve with Autologous Tissue Substitute, Open Approach |
| | | 0DUC0JZ | Supplement Ileocecal Valve with Synthetic Substitute, Open Approach |
| | | 0DUC0KZ | Supplement Ileocecal Valve with Nonautologous Tissue Substitute, Open Approach |
| | | 0DUC47Z | Supplement Ileocecal Valve with Autologous Tissue Substitute, Percutaneous Endoscopic Approach |
| | | 0DUC4JZ | Supplement Ileocecal Valve with Synthetic Substitute, Percutaneous Endoscopic Approach |
| | | 0DUC4KZ | Supplement Ileocecal Valve with Nonautologous Tissue Substitute, Percutaneous Endoscopic Approach |
| | | 0DUC77Z | Supplement Ileocecal Valve with Autologous Tissue Substitute, Via Natural or Artificial Opening |
| | | 0DUC7JZ | Supplement Ileocecal Valve with Synthetic Substitute, Via Natural or Artificial Opening |
| | | 0DUC7KZ | Supplement Ileocecal Valve with Nonautologous Tissue Substitute, Via Natural or Artificial Opening |
| | | 0DUU07Z | Supplement Omentum with Autologous Tissue Substitute, Open Approach |
| | | 0DUU0JZ | Supplement Omentum with Synthetic Substitute, Open Approach |
| | | 0DUU0KZ | Supplement Omentum with Nonautologous Tissue Substitute, Open Approach |
| | | 0DUU47Z | Supplement Omentum with Autologous Tissue Substitute, Percutaneous Endoscopic Approach |
| | | 0DUU4JZ | Supplement Omentum with Synthetic Substitute, Percutaneous Endoscopic Approach |
| | | 0DUU4KZ | Supplement Omentum with Nonautologous Tissue Substitute, Percutaneous Endoscopic Approach |
| | | 0DUV07Z | Supplement Mesentery with Autologous Tissue Substitute, Open Approach |
| | | 0DUV0JZ | Supplement Mesentery with Synthetic Substitute, Open Approach |
| | | 0DUV0KZ | Supplement Mesentery with Nonautologous Tissue Substitute, Open Approach |
| | | 0DUV47Z | Supplement Mesentery with Autologous Tissue Substitute, Percutaneous Endoscopic Approach |
| | | 0DUV4JZ | Supplement Mesentery with Synthetic Substitute, Percutaneous Endoscopic Approach |
| | | 0DUV4KZ | Supplement Mesentery with Nonautologous Tissue Substitute, Percutaneous Endoscopic Approach |
| | | 0DV44CZ | Restriction of Esophagogastric Junction with Extraluminal Device, Percutaneous Endoscopic Approach |
| | | 0DV44DZ | Restriction of Esophagogastric Junction with Intraluminal Device, Percutaneous Endoscopic Approach |
| | | 0DV44ZZ | Restriction of Esophagogastric Junction, Percutaneous Endoscopic Approach |
| | | 0DV80CZ | Restriction of Small Intestine with Extraluminal Device, Open Approach |
| | | 0DV80DZ | Restriction of Small Intestine with Intraluminal Device, Open Approach |
| | | 0DV80ZZ | Restriction of Small Intestine, Open Approach |
| | | 0DV83CZ | Restriction of Small Intestine with Extraluminal Device, Percutaneous Approach |
| | | 0DV83DZ | Restriction of Small Intestine with Intraluminal Device, Percutaneous Approach |
| | | 0DV84CZ | Restriction of Small Intestine with Extraluminal Device, Percutaneous Endoscopic Approach |
| | | 0DV84DZ | Restriction of Small Intestine with Intraluminal Device, Percutaneous Endoscopic Approach |
| | | 0DV84ZZ | Restriction of Small Intestine, Percutaneous Endoscopic Approach |
| | | 0DV87DZ | Restriction of Small Intestine with Intraluminal Device, Via Natural or Artificial Opening |
| | | 0DV87ZZ | Restriction of Small Intestine, Via Natural or Artificial Opening |
| | | 0DV90CZ | Restriction of Duodenum with Extraluminal Device, Open Approach |
| | | 0DV90DZ | Restriction of Duodenum with Intraluminal Device, Open Approach |
| | | 0DV90ZZ | Restriction of Duodenum, Open Approach |
| | | 0DV93CZ | Restriction of Duodenum with Extraluminal Device, Percutaneous Approach |
| | | 0DV93DZ | Restriction of Duodenum with Intraluminal Device, Percutaneous Approach |
| | | 0DV93ZZ | Restriction of Duodenum, Percutaneous Approach |
| | | 0DV94CZ | Restriction of Duodenum with Extraluminal Device, Percutaneous Endoscopic Approach |
| | | 0DV94DZ | Restriction of Duodenum with Intraluminal Device, Percutaneous Endoscopic Approach |
| | | 0DV94ZZ | Restriction of Duodenum, Percutaneous Endoscopic Approach |
| | | 0DV97DZ | Restriction of Duodenum with Intraluminal Device, Via Natural or Artificial Opening |
| | | 0DV97ZZ | Restriction of Duodenum, Via Natural or Artificial Opening |

© 2019 Lewis KH et al. *JAMA Network Open*.

| | | | |
|---|---|---|---|
| OTHER ABDOMINAL OPERATION | ICD-10 Procedure | 0DVA0CZ | Restriction of Jejunum with Extraluminal Device, Open Approach |
| | | 0DVA0DZ | Restriction of Jejunum with Intraluminal Device, Open Approach |
| | | 0DVA0ZZ | Restriction of Jejunum, Open Approach |
| | | 0DVA4CZ | Restriction of Jejunum with Extraluminal Device, Percutaneous Endoscopic Approach |
| | | 0DVA4DZ | Restriction of Jejunum with Intraluminal Device, Percutaneous Endoscopic Approach |
| | | 0DVA4ZZ | Restriction of Jejunum, Percutaneous Endoscopic Approach |
| | | 0DVA7DZ | Restriction of Jejunum with Intraluminal Device, Via Natural or Artificial Opening |
| | | 0DVA7ZZ | Restriction of Jejunum, Via Natural or Artificial Opening |
| | | 0DVB0CZ | Restriction of Ileum with Extraluminal Device, Open Approach |
| | | 0DVB0DZ | Restriction of Ileum with Intraluminal Device, Open Approach |
| | | 0DVB0ZZ | Restriction of Ileum, Open Approach |
| | | 0DVB4CZ | Restriction of Ileum with Extraluminal Device, Percutaneous Endoscopic Approach |
| | | 0DVB4DZ | Restriction of Ileum with Intraluminal Device, Percutaneous Endoscopic Approach |
| | | 0DVB4ZZ | Restriction of Ileum, Percutaneous Endoscopic Approach |
| | | 0DVB7DZ | Restriction of Ileum with Intraluminal Device, Via Natural or Artificial Opening |
| | | 0DVB7ZZ | Restriction of Ileum, Via Natural or Artificial Opening |
| | | 0DVC0CZ | Restriction of Ileocecal Valve with Extraluminal Device, Open Approach |
| | | 0DVC0DZ | Restriction of Ileocecal Valve with Intraluminal Device, Open Approach |
| | | 0DVC0ZZ | Restriction of Ileocecal Valve, Open Approach |
| | | 0DVC4CZ | Restriction of Ileocecal Valve with Extraluminal Device, Percutaneous Endoscopic Approach |
| | | 0DVC4DZ | Restriction of Ileocecal Valve with Intraluminal Device, Percutaneous Endoscopic Approach |
| | | 0DVC4ZZ | Restriction of Ileocecal Valve, Percutaneous Endoscopic Approach |
| | | 0DVC7DZ | Restriction of Ileocecal Valve with Intraluminal Device, Via Natural or Artificial Opening |
| | | 0DVC7ZZ | Restriction of Ileocecal Valve, Via Natural or Artificial Opening |
| | | 0DW000Z | Revision of Drainage Device in Upper Intestinal Tract, Open Approach |
| | | 0DW002Z | Revision of Monitoring Device in Upper Intestinal Tract, Open Approach |
| | | 0DW007Z | Revision of Autologous Tissue Substitute in Upper Intestinal Tract, Open Approach |
| | | 0DW00CZ | Revision of Extraluminal Device in Upper Intestinal Tract, Open Approach |
| | | 0DW00DZ | Revision of Intraluminal Device in Upper Intestinal Tract, Open Approach |
| | | 0DW00JZ | Revision of Synthetic Substitute in Upper Intestinal Tract, Open Approach |
| | | 0DW00KZ | Revision of Nonautologous Tissue Substitute in Upper Intestinal Tract, Open Approach |
| | | 0DW040Z | Revision of Drainage Device in Upper Intestinal Tract, Percutaneous Endoscopic Approach |
| | | 0DW042Z | Revision of Monitoring Device in Upper Intestinal Tract, Percutaneous Endoscopic Approach |
| | | 0DW047Z | Revision of Autologous Tissue Substitute in Upper Intestinal Tract, Percutaneous Endoscopic Approach |
| | | 0DW04CZ | Revision of Extraluminal Device in Upper Intestinal Tract, Percutaneous Endoscopic Approach |
| | | 0DW04DZ | Revision of Intraluminal Device in Upper Intestinal Tract, Percutaneous Endoscopic Approach |
| | | 0DW04JZ | Revision of Synthetic Substitute in Upper Intestinal Tract, Percutaneous Endoscopic Approach |
| | | 0DW04KZ | Revision of Nonautologous Tissue Substitute in Upper Intestinal Tract, Percutaneous Endoscopic Approach |
| | | 0DW070Z | Revision of Drainage Device in Upper Intestinal Tract, Via Natural or Artificial Opening |
| | | 0DW072Z | Revision of Monitoring Device in Upper Intestinal Tract, Via Natural or Artificial Opening |
| | | 0DW077Z | Revision of Autologous Tissue Substitute in Upper Intestinal Tract, Via Natural or Artificial Opening |
| | | 0DW07CZ | Revision of Extraluminal Device in Upper Intestinal Tract, Via Natural or Artificial Opening |
| | | 0DW07DZ | Revision of Intraluminal Device in Upper Intestinal Tract, Via Natural or Artificial Opening |
| | | 0DW07JZ | Revision of Synthetic Substitute in Upper Intestinal Tract, Via Natural or Artificial Opening |
| | | 0DW07KZ | Revision of Nonautologous Tissue Substitute in Upper Intestinal Tract, Via Natural or Artificial Opening |
| | | 0DW600Z | Revision of Drainage Device in Stomach, Open Approach |
| | | 0DW602Z | Revision of Monitoring Device in Stomach, Open Approach |
| | | 0DW607Z | Revision of Autologous Tissue Substitute in Stomach, Open Approach |
| | | 0DW60CZ | Revision of Extraluminal Device in Stomach, Open Approach |
| | | 0DW60DZ | Revision of Intraluminal Device in Stomach, Open Approach |
| | | 0DW60JZ | Revision of Synthetic Substitute in Stomach, Open Approach |
| | | 0DW60KZ | Revision of Nonautologous Tissue Substitute in Stomach, Open Approach |
| | | 0DW60MZ | Revision of Stimulator Lead in Stomach, Open Approach |
| | | 0DW60YZ | Revision of Other Device in Stomach, Open Approach |
| | | 0DW640Z | Revision of Drainage Device in Stomach, Percutaneous Endoscopic Approach |
| | | 0DW642Z | Revision of Monitoring Device in Stomach, Percutaneous Endoscopic Approach |
| | | 0DW647Z | REV AUTO TISS SUBST STOM PERQ ENDO |
| | | 0DW64DZ | Revision of Intraluminal Device in Stomach, Percutaneous Endoscopic Approach |
| | | 0DW64JZ | Revision of Synthetic Substitute in Stomach, Percutaneous Endoscopic Approach |
| | | 0DW64KZ | Revision of Nonautologous Tissue Substitute in Stomach, Percutaneous Endoscopic Approach |
| | | 0DW64MZ | Revision of Stimulator Lead in Stomach, Percutaneous Endoscopic Approach |
| | | 0DW64YZ | Revision of Other Device in Stomach, Percutaneous Endoscopic Approach |
| | | 0DW670Z | Revision of Drainage Device in Stomach, Via Natural or Artificial Opening |
| | | 0DW672Z | Revision of Monitoring Device in Stomach, Via Natural or Artificial Opening |
| | | 0DW677Z | Revision of Autologous Tissue Substitute in Stomach, Via Natural or Artificial Opening |
| | | 0DW67CZ | Revision of Extraluminal Device in Stomach, Via Natural or Artificial Opening |
| | | 0DW67DZ | Revision of Intraluminal Device in Stomach, Via Natural or Artificial Opening |
| | | 0DW67JZ | Revision of Synthetic Substitute in Stomach, Via Natural or Artificial Opening |
| | | 0DW67KZ | Revision of Nonautologous Tissue Substitute in Stomach, Via Natural or Artificial Opening |
| | | 0DW67YZ | Revision of Other Device in Stomach, Via Natural or Artificial Opening |
| | | 0DW807Z | Revision of Autologous Tissue Substitute in Small Intestine, Open Approach |
| | | 0DW80JZ | Revision of Synthetic Substitute in Small Intestine, Open Approach |
| | | 0DW80KZ | Revision of Nonautologous Tissue Substitute in Small Intestine, Open Approach |
| | | 0DW847Z | Revision of Autologous Tissue Substitute in Small Intestine, Percutaneous Endoscopic Approach |
| | | 0DW84JZ | Revision of Synthetic Substitute in Small Intestine, Percutaneous Endoscopic Approach |
| | | 0DW84KZ | Revision of Nonautologous Tissue Substitute in Small Intestine, Percutaneous Endoscopic Approach |
| | | 0DW877Z | Revision of Autologous Tissue Substitute in Small Intestine, Via Natural or Artificial Opening |
| | | 0DW87JZ | Revision of Synthetic Substitute in Small Intestine, Via Natural or Artificial Opening |
| | | 0DW87KZ | Revision of Nonautologous Tissue Substitute in Small Intestine, Via Natural or Artificial Opening |
| | | 0DWU07Z | Revision of Autologous Tissue Substitute in Omentum, Open Approach |

© 2019 Lewis KH et al. *JAMA Network Open*.

| | | | |
|---|---|---|---|
| OTHER ABDOMINAL OPERATION | ICD-10 Procedure | 0DWU0JZ | Revision of Synthetic Substitute in Omentum, Open Approach |
| | | 0DWU0KZ | Revision of Nonautologous Tissue Substitute in Omentum, Open Approach |
| | | 0DWU37Z | Revision of Autologous Tissue Substitute in Omentum, Percutaneous Approach |
| | | 0DWU3JZ | Revision of Synthetic Substitute in Omentum, Percutaneous Approach |
| | | 0DWU3KZ | Revision of Nonautologous Tissue Substitute in Omentum, Percutaneous Approach |
| | | 0DWU47Z | Revision of Autologous Tissue Substitute in Omentum, Percutaneous Endoscopic Approach |
| | | 0DWU4JZ | Revision of Synthetic Substitute in Omentum, Percutaneous Endoscopic Approach |
| | | 0DWU4KZ | Revision of Nonautologous Tissue Substitute in Omentum, Percutaneous Endoscopic Approach |
| | | 0DWV07Z | Revision of Autologous Tissue Substitute in Mesentery, Open Approach |
| | | 0DWV0JZ | Revision of Synthetic Substitute in Mesentery, Open Approach |
| | | 0DWV0KZ | Revision of Nonautologous Tissue Substitute in Mesentery, Open Approach |
| | | 0DWV37Z | Revision of Autologous Tissue Substitute in Mesentery, Percutaneous Approach |
| | | 0DWV3JZ | Revision of Synthetic Substitute in Mesentery, Percutaneous Approach |
| | | 0DWV3KZ | Revision of Nonautologous Tissue Substitute in Mesentery, Percutaneous Approach |
| | | 0DWV47Z | Revision of Autologous Tissue Substitute in Mesentery, Percutaneous Endoscopic Approach |
| | | 0DWV4JZ | Revision of Synthetic Substitute in Mesentery, Percutaneous Endoscopic Approach |
| | | 0DWV4KZ | Revision of Nonautologous Tissue Substitute in Mesentery, Percutaneous Endoscopic Approach |
| | | 0DWW07Z | Revision of Autologous Tissue Substitute in Peritoneum, Open Approach |
| | | 0DWW0JZ | Revision of Synthetic Substitute in Peritoneum, Open Approach |
| | | 0DWW0KZ | Revision of Nonautologous Tissue Substitute in Peritoneum, Open Approach |
| | | 0DWW47Z | Revision of Autologous Tissue Substitute in Peritoneum, Percutaneous Endoscopic Approach |
| | | 0DWW4JZ | Revision of Synthetic Substitute in Peritoneum, Percutaneous Endoscopic Approach |
| | | 0DWW4KZ | Revision of Nonautologous Tissue Substitute in Peritoneum, Percutaneous Endoscopic Approach |
| | | 0DY60Z0 | Transplantation of Stomach, Allogeneic, Open Approach |
| | | 0DY60Z1 | Transplantation of Stomach, Syngeneic, Open Approach |
| | | 0DY60Z2 | Transplantation of Stomach, Zooplastic, Open Approach |
| | | 0FJ00ZZ | Inspection of Liver, Open Approach |
| | | 0FJ04ZZ | Inspection of Liver, Percutaneous Endoscopic Approach |
| | | 0FJD4ZZ | Inspection of Pancreatic Duct, Percutaneous Endoscopic Approach |
| | | 0FJG4ZZ | Inspection of Pancreas, Percutaneous Endoscopic Approach |
| | | 0FN00ZZ | Release Liver, Open Approach |
| | | 0FN04ZZ | Release Liver, Percutaneous Endoscopic Approach |
| | | 0FN10ZZ | Release Right Lobe Liver, Open Approach |
| | | 0FN14ZZ | Release Right Lobe Liver, Percutaneous Endoscopic Approach |
| | | 0FN20ZZ | Release Left Lobe Liver, Open Approach |
| | | 0FN24ZZ | Release Left Lobe Liver, Percutaneous Endoscopic Approach |
| | | 0FNC0ZZ | Release Ampulla of Vater, Open Approach |
| | | 0FNC4ZZ | Release Ampulla of Vater, Percutaneous Endoscopic Approach |
| | | 0FNC7ZZ | Release Ampulla of Vater, Via Natural or Artificial Opening |
| | | 0FND0ZZ | Release Pancreatic Duct, Open Approach |
| | | 0FND4ZZ | Release Pancreatic Duct, Percutaneous Endoscopic Approach |
| | | 0FND7ZZ | Release Pancreatic Duct, Via Natural or Artificial Opening |
| | | 0FNF0ZZ | Release Accessory Pancreatic Duct, Open Approach |
| | | 0FNF4ZZ | Release Accessory Pancreatic Duct, Percutaneous Endoscopic Approach |
| | | 0FNF7ZZ | Release Accessory Pancreatic Duct, Via Natural or Artificial Opening |
| | | 0FNG0ZZ | Release Pancreas, Open Approach |
| | | 0FNG4ZZ | Release Pancreas, Percutaneous Endoscopic Approach |
| | | 0FNG8ZZ | Release Pancreas, Via Natural or Artificial Opening Endoscopic |
| | | 0W3G0ZZ | Control Bleeding in Peritoneal Cavity, Open Approach |
| | | 0W3G4ZZ | Control Bleeding in Peritoneal Cavity, Percutaneous Endoscopic Approach |
| | | 0W3H0ZZ | Control Bleeding in Retroperitoneum, Open Approach |
| | | 0W3P0ZZ | Control Bleeding in Gastrointestinal Tract, Open Approach |
| | | 0W9F00Z | Drainage of Abdominal Wall with Drainage Device, Open Approach |
| | | 0W9F0ZZ | Drainage of Abdominal Wall, Open Approach |
| | | 0W9G00Z | Drainage of Peritoneal Cavity with Drainage Device, Open Approach |
| | | 0W9G0ZZ | Drainage of Peritoneal Cavity, Open Approach |
| | | 0W9G40Z | DRAIN PERITONEAL CAV DRN PC ENDO |
| | | 0W9G4ZZ | DRAIN PERITONEAL CAVITY PERQ ENDO |
| | | 0W9H00Z | Drainage of Retroperitoneum with Drainage Device, Open Approach |
| | | 0W9H0ZZ | Drainage of Retroperitoneum, Open Approach |
| | | 0W9H40Z | Drainage of Retroperitoneum with Drainage Device, Percutaneous Endoscopic Approach |
| | | 0W9H4ZZ | Drainage of Retroperitoneum, Percutaneous Endoscopic Approach |
| | | 0WBF0ZZ | Excision of Abdominal Wall, Open Approach |
| | | 0WBF4ZZ | Excision of Abdominal Wall, Percutaneous Endoscopic Approach |
| | | 0WBFXZ2 | Excision of Abdominal Wall, Stoma, External Approach |
| | | 0WBFXZZ | Excision of Abdominal Wall, External Approach |
| | | 0WBH0ZZ | Excision of Retroperitoneum, Open Approach |
| | | 0WBH4ZZ | Excision of Retroperitoneum, Percutaneous Endoscopic Approach |
| | | 0WCH0ZZ | Extirpation of Matter from Retroperitoneum, Open Approach |
| | | 0WCH4ZZ | Extirpation of Matter from Retroperitoneum, Percutaneous Endoscopic Approach |
| | | 0WCP0ZZ | Extirpation of Matter from Gastrointestinal Tract, Open Approach |
| | | 0WCP4ZZ | Extirpation of Matter from Gastrointestinal Tract, Percutaneous Endoscopic Approach |
| | | 0WHF0YZ | Insertion of Other Device into Abdominal Wall, Open Approach |
| | | 0WHF4YZ | Insertion of Other Device into Abdominal Wall, Percutaneous Endoscopic Approach |
| | | 0WHG0YZ | Insertion of Other Device into Peritoneal Cavity, Open Approach |
| | | 0WHG4YZ | Insertion of Other Device into Peritoneal Cavity, Percutaneous Endoscopic Approach |
| | | 0WHH0YZ | Insertion of Other Device into Retroperitoneum, Open Approach |
| | | 0WHH4YZ | Insertion of Other Device into Retroperitoneum, Percutaneous Endoscopic Approach |
| | | 0WHP03Z | Insertion of Infusion Device into Gastrointestinal Tract, Open Approach |
| | | 0WJF0ZZ | Inspection of Abdominal Wall, Open Approach |

© 2019 Lewis KH et al. *JAMA Network Open*.

| | | | |
|---|---|---|---|
| OTHER ABDOMINAL OPERATION | ICD-10 Procedure | 0WJF4ZZ | INSPECTION ABDOMINAL WALL PERQ ENDO |
| | | 0WJG0ZZ | Inspection of Peritoneal Cavity, Open Approach |
| | | 0WJG4ZZ | Inspection of Peritoneal Cavity, Percutaneous Endoscopic Approach |
| | | 0WJH0ZZ | Inspection of Retroperitoneum, Open Approach |
| | | 0WJP0ZZ | Inspection of Gastrointestinal Tract, Open Approach |
| | | 0WJP4ZZ | Inspection of Gastrointestinal Tract, Percutaneous Endoscopic Approach |
| | | 0WMF0ZZ | Reattachment of Abdominal Wall, Open Approach |
| | | 0WPF00Z | Removal of Drainage Device from Abdominal Wall, Open Approach |
| | | 0WPF07Z | Removal of Autologous Tissue Substitute from Abdominal Wall, Open Approach |
| | | 0WPF0JZ | REMOVAL SYNTH SUB ABD WALL OPEN |
| | | 0WPF0KZ | Removal of Nonautologous Tissue Substitute from Abdominal Wall, Open Approach |
| | | 0WPF0YZ | Removal of Other Device from Abdominal Wall, Open Approach |
| | | 0WPF40Z | Removal of Drainage Device from Abdominal Wall, Percutaneous Endoscopic Approach |
| | | 0WPF47Z | Removal of Autologous Tissue Substitute from Abdominal Wall, Percutaneous Endoscopic Approach |
| | | 0WPF4JZ | Removal of Synthetic Substitute from Abdominal Wall, Percutaneous Endoscopic Approach |
| | | 0WPF4KZ | Removal of Nonautologous Tissue Substitute from Abdominal Wall, Percutaneous Endoscopic Approach |
| | | 0WPF4YZ | Removal of Other Device from Abdominal Wall, Percutaneous Endoscopic Approach |
| | | 0WPG00Z | Removal of Drainage Device from Peritoneal Cavity, Open Approach |
| | | 0WPG0JZ | Removal of Synthetic Substitute from Peritoneal Cavity, Open Approach |
| | | 0WPG0YZ | Removal of Other Device from Peritoneal Cavity, Open Approach |
| | | 0WPG40Z | Removal of Drainage Device from Peritoneal Cavity, Percutaneous Endoscopic Approach |
| | | 0WPG4JZ | Removal of Synthetic Substitute from Peritoneal Cavity, Percutaneous Endoscopic Approach |
| | | 0WPG4YZ | Removal of Other Device from Peritoneal Cavity, Percutaneous Endoscopic Approach |
| | | 0WPH00Z | Removal of Drainage Device from Retroperitoneum, Open Approach |
| | | 0WPH0YZ | Removal of Other Device from Retroperitoneum, Open Approach |
| | | 0WPH40Z | Removal of Drainage Device from Retroperitoneum, Percutaneous Endoscopic Approach |
| | | 0WPH4YZ | Removal of Other Device from Retroperitoneum, Percutaneous Endoscopic Approach |
| | | 0WPP0YZ | Removal of Other Device from Gastrointestinal Tract, Open Approach |
| | | 0WQFXZ2 | REPAIR ABDOMINAL WALL STOMA EXT |
| | | 0WWF00Z | Revision of Drainage Device in Abdominal Wall, Open Approach |
| | | 0WWF07Z | Revision of Autologous Tissue Substitute in Abdominal Wall, Open Approach |
| | | 0WWF0JZ | Revision of Synthetic Substitute in Abdominal Wall, Open Approach |
| | | 0WWF0KZ | Revision of Nonautologous Tissue Substitute in Abdominal Wall, Open Approach |
| | | 0WWF0YZ | Revision of Other Device in Abdominal Wall, Open Approach |
| | | 0WWF40Z | Revision of Drainage Device in Abdominal Wall, Percutaneous Endoscopic Approach |
| | | 0WWF47Z | Revision of Autologous Tissue Substitute in Abdominal Wall, Percutaneous Endoscopic Approach |
| | | 0WWF4JZ | Revision of Synthetic Substitute in Abdominal Wall, Percutaneous Endoscopic Approach |
| | | 0WWF4KZ | Revision of Nonautologous Tissue Substitute in Abdominal Wall, Percutaneous Endoscopic Approach |
| | | 0WWF4YZ | Revision of Other Device in Abdominal Wall, Percutaneous Endoscopic Approach |
| | | 0WWG00Z | Revision of Drainage Device in Peritoneal Cavity, Open Approach |
| | | 0WWG0JZ | Revision of Synthetic Substitute in Peritoneal Cavity, Open Approach |
| | | 0WWG0YZ | Revision of Other Device in Peritoneal Cavity, Open Approach |
| | | 0WWG40Z | Revision of Drainage Device in Peritoneal Cavity, Percutaneous Endoscopic Approach |
| | | 0WWG4JZ | Revision of Synthetic Substitute in Peritoneal Cavity, Percutaneous Endoscopic Approach |
| | | 0WWG4YZ | Revision of Other Device in Peritoneal Cavity, Percutaneous Endoscopic Approach |
| | | 0WWH00Z | Revision of Drainage Device in Retroperitoneum, Open Approach |
| | | 0WWH0YZ | Revision of Other Device in Retroperitoneum, Open Approach |
| | | 0WWH40Z | Revision of Drainage Device in Retroperitoneum, Percutaneous Endoscopic Approach |
| | | 0WWH4YZ | Revision of Other Device in Retroperitoneum, Percutaneous Endoscopic Approach |
| | | 0WWP0YZ | Revision of Other Device in Gastrointestinal Tract, Open Approach |
| BARIATRIC REVISION OR CONVERSION | CPT | 43620 | Gastrectomy total with esophagoenterostomy |
| | | 43621 | Removal of stomach |
| | | 43622 | Gastrectomy total w/intesintal pouch formation |
| | | 43631 | Removal of stomach, partial |
| | | 43632 | Gastrectomy partial, distal w/g-jejunostomy |
| | | 43633 | Removal of stomach, partial |
| | | 43634 | Removal of stomach, partial |
| | | 43638 | Gastrectomy partial, prox w/ esophagogastrostomy |
| | | 43639 | Gastrectomy |
| | | 43644 | Lap gastric bypass/roux-en-y |
| | | 43645 | Lap gastr bypass incl smll i |
| | | 43770 | Lap band placement |
| | | 43771 | Lap gastric restrictive procedure |
| | | 43772 | Lap gastric restrictive procedure |
| | | 43773 | Lap gastric restrictive procedure |
| | | 43774 | Lap gastric restrictive procedure |
| | | 43775 | Vertical sleeve gastrectomy |
| | | 43820 | Fusion of stomach and bowel |
| | | 43842 | Gastric restrictive proc w/o gast bypass |
| | | 43843 | Gastroplasty w/o v-band |
| | | 43844 | Lap gast restrictive proc w/ GB & Roux en Y gastroenterostomy |
| | | 43845 | Gast restrictive w/prtl gastrectomy 50-100cm |
| | | 43846 | Gastric bypass for obesity |
| | | 43847 | Gastric bypass incl small i |
| | | 43848 | Revision gastroplasty |
| | | 43850 | Revise stomach bowel fusion |
| | | 43855 | Revision gastroduodenal anast |
| | | 43860 | Revise stomach bowel fusion |
| | | 43865 | Revision gastroduodenal anast |
| | | 43880 | Repair stomach-bowel fistula |

© 2019 Lewis KH et al. *JAMA Network Open*.

| | | | |
|---|---|---|---|
| | | 43886<br>43887<br>43888 | Gast restrictive proc open<br>Gast restrictive proc open<br>Gast restrictive proc open |

| | | | |
|---|---|---|---|
| BARIATRIC REVISION OR CONVERSION | ICD-9 Procedure | 43.5<br>43.6<br>43.7<br>43.8<br>43.81<br>43.82<br>43.89<br>43.9<br>43.91<br>43.99<br>44.3<br>44.31<br>44.38<br>44.39<br>44.5<br>44.68<br>44.95<br>44.96<br>44.97<br>46.93 | Partial gastrectomy with anastomosis to esophagus<br>Partial gastrectomy with anastomosis to duodenum<br>Partial gastrect w/anastom jejunum<br>Other partial gastrectomy<br>Partial gastrectomy w jejun transpos<br>Laparoscopic vertical slv gastrectomy<br>Other partial gastrectomy<br>Total gastrectomy<br>Total gastrectomy w intest transpos<br>Other total gastrectomy<br>Gastroenterostomy without gastrectomy<br>High gastric bypass<br>Laparoscopic gastroenterostomy<br>Other gastroenterostomy<br>Revision gastric anastomosis<br>Laparoscopic gastroplasty<br>Lap gastric restrictive proc<br>Lap revis gastric restrictive proc<br>Lap removal gastric restrictive device<br>Revision anast sm intestine |
| BARIATRIC REVISION OR CONVERSION | ICD-10 Procedure | 0D13079<br>0D1307A<br>0D1307B<br>0D16079<br>0D1607A<br>0D1607B<br>0D1607L<br>0D160J9<br>0D160JA<br>0D160JB<br>0D160JL<br>0D160K9<br>0D160KA<br>0D160KB<br>0D160KL<br>0D160Z9<br>0D160ZA<br>0D160ZB<br>0D160ZL<br>0D16479<br>0D1647A<br>0D1647B<br>0D1647L<br>0D164J9<br>0D164JA<br>0D164JB<br>0D164JL<br>0D164K9<br>0D164KA<br>0D164KB<br>0D164KL<br>0D164Z9<br>0D164ZA<br>0D164ZB<br>0D164ZL<br>0DB40ZX<br>0DB40ZZ<br>0DB44ZZ<br>0DB47ZX<br>0DB47ZZ<br>0DB60Z3<br>0DB60ZZ<br>0DB64Z3<br>0DB64ZZ<br>0DB67Z3<br>0DB67ZZ<br>0DB70ZZ<br>0DB77ZZ<br>0DP64CZ<br>0DT40ZZ<br>0DT44ZZ<br>0DT47ZZ<br>0DT60ZZ<br>0DT64ZZ<br>0DT67ZZ<br>0DT70ZZ<br>0DT74ZZ<br>0DT77ZZ<br>0DV60CZ | Bypass Lower Esophagus to Duodenum with Autologous Tissue Substitute, Open Approach<br>Bypass Lower Esophagus to Jejunum with Autologous Tissue Substitute, Open Approach<br>Bypass Lower Esophagus to Ileum with Autologous Tissue Substitute, Open Approach<br>Bypass Stomach to Duodenum with Autologous Tissue Substitute, Open Approach<br>Bypass Stomach to Jejunum with Autologous Tissue Substitute, Open Approach<br>Bypass Stomach to Ileum with Autologous Tissue Substitute, Open Approach<br>Bypass Stomach to Transverse Colon with Autologous Tissue Substitute, Open Approach<br>Bypass Stomach to Duodenum with Synthetic Substitute, Open Approach<br>Bypass Stomach to Jejunum with Synthetic Substitute, Open Approach<br>Bypass Stomach to Ileum with Synthetic Substitute, Open Approach<br>Bypass Stomach to Transverse Colon with Synthetic Substitute, Open Approach<br>Bypass Stomach to Duodenum with Nonautologous Tissue Substitute, Open Approach<br>Bypass Stomach to Jejunum with Nonautologous Tissue Substitute, Open Approach<br>Bypass Stomach to Ileum with Nonautologous Tissue Substitute, Open Approach<br>Bypass Stomach to Transverse Colon with Nonautologous Tissue Substitute, Open Approach<br>Bypass Stomach to Duodenum, Open Approach<br>Bypass stomach jejunum open approach<br>Bypass Stomach to Ileum, Open Approach<br>Bypass Stomach to Transverse Colon, Open Approach<br>Bypass Stomach to Duodenum with Autologous Tissue Substitute, Percutaneous Endoscopic Approach<br>Bypass Stomach to Jejunum with Autologous Tissue Substitute, Percutaneous Endoscopic Approach<br>Bypass Stomach to Ileum with Autologous Tissue Substitute, Percutaneous Endoscopic Approach<br>Bypass Stomach to Transverse Colon with Autologous Tissue Substitute, Percutaneous Endoscopic Approach<br>Bypass Stomach to Duodenum with Synthetic Substitute, Percutaneous Endoscopic Approach<br>Bypass Stomach to Jejunum with Synthetic Substitute, Percutaneous Endoscopic Approach<br>Bypass Stomach to Ileum with Synthetic Substitute, Percutaneous Endoscopic Approach<br>Bypass Stomach to Transverse Colon with Synthetic Substitute, Percutaneous Endoscopic Approach<br>Bypass Stomach to Duodenum with Nonautologous Tissue Substitute, Percutaneous Endoscopic Approach<br>Bypass Stomach to Jejunum with Nonautologous Tissue Substitute, Percutaneous Endoscopic Approach<br>Bypass Stomach to Ileum with Nonautologous Tissue Substitute, Percutaneous Endoscopic Approach<br>Bypass Stomach to Transverse Colon with Nonautol Tissue Substitute, Percutaneous Endoscopic Approach<br>Bypass Stomach to Duodenum, Percutaneous Endoscopic Approach<br>Bypass Stomach to Jejunum, Percutaneous Endoscopic Approach<br>Bypass Stomach to Ileum, Percutaneous Endoscopic Approach<br>Bypass Stomach to Transverse Colon, Percutaneous Endoscopic Approach<br>Excision of Esophagogastric Junction, Open Approach, Diagnostic<br>Excision of Esophagogastric Junction, Open Approach<br>Excision of Esophagogastric Junction, Percutaneous Endoscopic Approach<br>Excision of Esophagogastric Junction, Via Natural or Artificial Opening, Diagnostic<br>Excision of Esophagogastric Junction, Via Natural or Artificial Opening<br>Excision of Stomach, Open Approach, Vertical<br>Excision of Stomach, Open Approach<br>Excision stomach perq endo vertical<br>Excision stomach percutaneous endo<br>Excision of Stomach, Via Natural or Artificial Opening, Vertical<br>Excision of Stomach, Via Natural or Artificial Opening<br>Excision of Stomach, Pylorus, Open Approach<br>Excision of Stomach, Pylorus, Via Natural or Artificial Opening<br>Removal of Extraluminal Device from Stomach, Percutaneous Endoscopic Approach<br>Resection of Esophagogastric Junction, Open Approach<br>Resection of Esophagogastric Junction, Percutaneous Endoscopic Approach<br>Resection of Esophagogastric Junction, Via Natural or Artificial Opening<br>Resection of Stomach, Open Approach<br>Resection of Stomach, Percutaneous Endoscopic Approach<br>Resection of Stomach, Via Natural or Artificial Opening<br>Resection of Stomach, Pylorus, Open Approach<br>Resection of Stomach, Pylorus, Percutaneous Endoscopic Approach<br>Resection of Stomach, Pylorus, Via Natural or Artificial Opening<br>Restriction of Stomach with Extraluminal Device, Open Approach |

© 2019 Lewis KH et al. *JAMA Network Open*.

| | | | |
|---|---|---|---|
| | | 0DV60DZ | Restriction of Stomach with Intraluminal Device, Open Approach |

| | | | |
|---|---|---|---|
| BARIATRIC REVISION OR CONVERSION | ICD-10 Procedure | 0DV60ZZ | Restriction of Stomach, Open Approach |
| | | 0DV64CZ | Restriction of Stomach with Extraluminal Device, Percutaneous Endoscopic Approach |
| | | 0DV64DZ | Restriction of Stomach with Intraluminal Device, Percutaneous Endoscopic Approach |
| | | 0DV64ZZ | Restriction of Stomach, Percutaneous Endoscopic Approach |
| | | 0DV67DZ | Restriction of Stomach with Intraluminal Device, Via Natural or Artificial Opening |
| | | 0DV67ZZ | Restriction of Stomach, Via Natural or Artificial Opening |
| | | 0DV70CZ | Restriction of Stomach, Pylorus with Extraluminal Device, Open Approach |
| | | 0DV70DZ | Restriction of Stomach, Pylorus with Intraluminal Device, Open Approach |
| | | 0DV70ZZ | Restriction of Stomach, Pylorus, Open Approach |
| | | 0DV74CZ | Restriction of Stomach, Pylorus with Extraluminal Device, Percutaneous Endoscopic Approach |
| | | 0DV74DZ | Restriction of Stomach, Pylorus with Intraluminal Device, Percutaneous Endoscopic Approach |
| | | 0DV74ZZ | Restriction of Stomach, Pylorus, Percutaneous Endoscopic Approach |
| | | 0DV77DZ | Restriction of Stomach, Pylorus with Intraluminal Device, Via Natural or Artificial Opening |
| | | 0DV77ZZ | Restriction of Stomach, Pylorus, Via Natural or Artificial Opening |
| | | 0DW64CZ | Revision of Extraluminal Device in Stomach, Percutaneous Endoscopic Approach |

© 2019 Lewis KH et al. *JAMA Network Open*.

| **eTable 2.** Procedures Subject to a 30-Day Washout Period Following the Date of the Index Bariatric Procedure[a] | |
|---|---|
| **Category** | **Procedures Codes Covered** |
| BILIARY PROCEDURE | All Procedures in category |
| ABDOMINAL WALL HERNIA REPAIR | All procedures in category |
| BARIATRIC REVISION OR CONVERSION | All procedures in category |
| OTHER ABDOMINAL OPERATION | CPT 43280 (Laparoscopic Fundoplasty)<br>CPT 43281 (Lap paraesophageal hernia repair)<br>CPT 43282 (Lap paraesophag hernia rpr w/mesh)<br>CPT 43327 (Fundoplasty)<br>CPT 43328 (Fundoplasty)<br>CPT 43332 (Transabdominal esophageal hiatal hernia repair)<br>CPT 43333 (Paraesophageal hernia repair)<br>CPT 43334 (Paraesophageal hernia repair)<br>CPT 43335 (Paraesophageal hernia repair)<br>CPT 43336 (Paraesophageal hernia repair)<br>CPT 43337 (Paraesophageal hernia repair)<br>CPT 43659 (Laparoscope procedure, stomach)<br>CPT 47379 (Laparoscope procedure, liver)<br>ICD-9 53.71 (Lap repair diaphragmatic hernia abdominal approach)<br>ICD-10 0BQT4ZZ (Repair Diaphragm, Perq Endo)<br>ICD-10 0BRT47Z (Replace Diaphragm with Autologous Tissue Substitute, Perq Endo)<br>ICD-10 0BRT4JZ (Replace Diaphragm with Synthetic Substitute, Perq Endo)<br>ICD-10 0BRT4KZ (Replace Diaphragm with Nonautologous Tissue Substitute, Perq Endo)<br>ICD-10 0BUT47Z (Supplement Diaphragm with Autologous Tissue Substitute, Perq Endo)<br>ICD-10 0BUT4JZ (Supplement Diaphragm with Synthetic Substitute, Perq Endo)<br>ICD-10 0BUT4KZ (Supplement Diaphragm with Nonautologous Tissue Substitute, Perq Endo) |
| *a - Following the index date, no procedures from this list were counted as reinterventions for the initial 30 post-operative days, because they more likely represented delayed or resubmitted claims from the index procedure as opposed to unique reoperations.* | |

© 2019 Lewis KH et al. *JAMA Network Open*.

| eTable 3. Procedure and Pharmacy Codes Used to Define Study Cohort and Baseline Comorbidities | |
|---|---|
| **(1) CPT, ICD-9, and ICD-10 Procedure Codes used to Define Study Cohorts** | |
| **Category** | **CPT-4 Codes, ICD-9-CM and ICD-10-CM Diagnosis or Procedure Codes** |
| **Patients were considered to have had a:** | **If:** |
| Laparoscopic RYGB | CPT codes: 43644 (Laparoscopy, surgical, gastric restrictive procedure; with gastric bypass and Roux-en-Y gastroenterostomy, Roux limb 150cm or less), 43645 (Laparoscopy, surgical, gastric restrictive procedure; with gastric bypass and small intestine reconstruction to limit absorption), 43844 (laparoscopic gastric restrictive and any open bypass, prior to October 1, 2015)<br>ICD-9 Procedure codes: 44.38 (laparoscopic gastroenterostomy) AND ICD-9 Diagnosis code: 278.01 (morbid obesity) coded on same day<br>ICD-10 Procedure codes: 0D1647A (bypass stomach to jejunum with autologous tissue substitute, percutaneous endoscopic approach), 0D1647B (bypass stomach to ileum with autologous tissue substitute, percutaneous endoscopic approach), 0D164ZA (bypass stomach to jejunum, percutaneous endoscopic approach) AND no 2nd excision code on same day, 0D163ZA (bypass stomach to jejunum, percutaneous approach) AND no 2nd excision code on same day, 0D1687A (bypass stomach to jejunum with autologous tissue substitute, endoscopic), 0D1687B (bypass stomach to ileum with autologous tissue substitute, via natural or artificial opening endoscopic), 0D168ZA (bypass stomach to jejunum, via natural or artificial opening endoscopic), 0D168ZB (bypass stomach to ileum, via natural or artificial opening endoscopic) AND ICD-10 Diagnosis code: E66.01 (morbid obesity) coded on same day |
| Vertical Sleeve Gastrectomy (VSG) | CPT code 43775 (laparoscopic longitudinal gastrectomy)<br>ICD-10 Procedure codes : 0DB60Z3 (excision of stomach, percutaneous endoscopic approach, vertical), or 0DB63Z3 (excision of stomach, open approach, vertical), 0DB64Z3 (excision of stomach, percutaneous approach, vertical) or 0DB67Z3 (excision of stomach, via natural or artificial opening, vertical) or 0DB68Z3 (excision of stomach, via natural or artificial opening endoscopic, vertical) if not associated with bypass codes (0D194ZA or 0D194ZB or 0D190ZA or 0D190ZB) (bypass duodenum to jejunum/ileum) on the same day AND ICD-10 Diagnosis code: E66.01 (morbid obesity) coded on same day |
| **(2) CPT, ICD-9, and ICD-10 Procedure Codes Applied as Additional Exclusion Criteria:** | |
| **Category** | **CPT-4 Codes, ICD-9-CM and ICD-10-CM Diagnosis or Procedure Codes** |
| **Patients were eliminated from the study cohort when they had:** | |
| Other Miscellaneous Bariatric Procedures | CPT codes: 43621 or 43633 (excision procedures on the stomach) ONLY if these codes are associated with ICD-9 Diagnosis code: 278.01 (morbid obesity) or ICD-10 Diagnosis Code: E66.01 (morbid obesity), 43770 (laparoscopic bariatric surgery procedures), 43846 (proximal; short limb), 43847 (distal; roux limb > 150 cm), 43842, 43843, 43845, 43999 (other procedures on the stomach) ONLY if these codes are associated with ICD-9 Diagnosis code: 278.01 (morbid obesity) or ICD-10 Diagnosis Code: E66.01 (morbid obesity)<br>ICD-9 Procedure codes: 43.89 (open and other partial gastrectomy), 44.68 (laparoscopic gastroplasty), 44.69 (other repair of stomach), 44.95 (laparoscopic gastric restrictive procedure), 45.51 (isolation of segment of small intestine), 45.91 (small-to-small intestinal anastomosis), 44.31 (high gastric bypass), 44.39 (other gastroenterostomy without gastrectomy) AND ICD-9 Diagnosis code 278.01 (morbid obesity) coded on same day<br>ICD-10 Procedure codes: 0D1607A (bypass stomach to jejunum with autologous tissue substitute, open approach), 0D160ZA (bypass stomach to jejunum open approach), 0D1607B (bypass stomach to ileum with autologous tissue substitute, open approach), 0D160ZB (bypass stomach to ileum, open approach), 0DV60CZ (restriction of stomach with extraluminal device, open approach), 0DV63CZ (restriction of stomach with extraluminal device, percutaneous approach), 0DV64CZ (restriction of stomach with extraluminal device, percutaneous endoscopic approach)(0D160ZA or 0D163ZA or 0D164ZA or 0D160ZB or 0D163ZB or 0D164ZB) (bypass stomach to jejunum/ileum) AND (0DB60ZZ or 0DB63ZZ or 0DB64ZZ) (excision of stomach) coded on same day, (0D194ZA or 0D194ZB or 0D190ZA or 0D190ZB) (bypass duodenum to jejunum/ileum) AND (0DB64Z3 or 0DB60Z3 or 0DB63Z3 or 0DB67Z3 or 0DB68Z3) (excision of stomach, vertical) coded on same day AND ICD-10 Diagnosis code E66.01 (morbid obesity) coded on same day |
| Evidence that procedure linked to GI malignancy rather than weight loss indications | Eliminate patients if they have any ICD-9 or ICD-10 Diagnosis codes for GI malignancy within two years and/or if they have any diagnosis or procedure codes for (repair of) perforated GI ulcer on the date of their index procedure or within 30 days prior.<br>ICD-9 Diagnosis codes: 150-150.9 (malignant neoplasm of esophagus), 151-151.9 (malignant neoplasm of stomach), 152-152.9 (malignant neoplasm of small intestine), 157-157.9 (malignant neoplasm of pancreas), 531.xx – 533.xx (perforated GI ulcer)<br>ICD-10 Diagnosis codes: C15.x (malignant neoplasm of esophagus), C16.x (malignant neoplasm of stomach), C17.x (malignant neoplasm of small intestine), C25.x (malignant neoplasm of pancreas), K25.x (gastric ulcer), K26.x (duodenal ulcer), K27.x (peptic ulcer) or CPT code: 43840 (Gastrorrhaphy) |
| Indication that this is not actually the first bariatric procedure for a given patient | Eliminate patients with CPT, HCPC, ICD-9 or ICD-10 procedure codes for any revisional procedure anywhere in the enrollment span prior to the index procedure:<br>CPT codes: 43771 (revision of gastric band), 43772 (removal of gastric band), 43773 (removal and replacement of gastric band), 43774 (removal of adjustable gastric band and port), 43848 (removal of band to RYGB, open), 43886 (revision of subcutaneous port, open), 43887 (early port infection AGB), 43860 (revision of GJ anastomosis), 44120 (enterectomy single with re-anastomosis, open), 43865 (revision of GJ anastomosis w/ reconstruction, w/w/o part gastrectomy), 43850 (revision of gastroduodenal anastomosis without vagotomy), 43855 (revision of GD anastomosis with vagotomy), 43888 (gastric restrictive procedure open – removal/replacement port), 43621 (total gastrectomy with RY reconstruction), 43639 (partial gastrectomy with vagotomy), 43634 (partial distal gastrectomy w/intestinal pouch), 43632 (partial distal gastrectomy w/ GJ), 43631 (partial distal gastrectomy w/ GD), 43638 (partial proximal gastrectomy w/ |

© 2019 Lewis KH et al. *JAMA Network Open*.

| | |
|---|---|
| | vagotomy), 43620 (total gastrectomy w/ esophagoenterostomy), 43622 (gastrectomy total w/ intestinal pouch) of HCPC codes: S2083 (adjustment gastric band)<br>ICD-9 Procedure codes: 44.97 (removal gastric band and port), 44.96 (replacement of gastric band and port), 44.5 (revision RYGB), 44.68 (laparoscopic gastroplasty), 44.3 (gastroenterostomy w/o gastrectomy), 43.5 (partial gastrectomy w/ anastomosis to esophagus), 43.8 (other partial gastrectomy), 43.9 (total gastrectomy), 43.6 (partial gastrectomy), 43.99 (other total gastrectomy), 43.7 (partial gastrectomy), 43.91 (total gastrectomy), 43.81 (partial gastrectomy)<br>ICD-10 Procedure codes: 0DP643Z or 0DW643Z (endoscopic removal/revision infusion device), 0DP64CZ or 0DW64CZ (endoscopic removal/revision extraluminal device), 0DQ60ZZ or 0DQ63ZZ or 0DQ64ZZ or 0DQ67ZZ or 0DQ68ZZ or 0DQ64ZZ (repair stomach), 0DB40ZZ or 0DB43ZZ or 0DB44ZZ or 0DB47ZZ (excision of esophagogastric junction), 0DT40ZZ or 0DT44ZZ or 0DT47ZZ or 0DT48ZZ (resection of esophagogastric junction), 0DB60ZZ or 0DB63ZZ or 0DB67ZZ (excision stomach), 0DT70ZZ or 0DT74ZZ or 0DT77ZZ or 0DT78ZZ or 0DT60ZZ or 0DT64ZZ or 0DT67ZZ or 0DT68ZZ (resection stomach), 0DV64CZ (endoscopic restriction of stomach extraluminal device), (0DB60ZZ or 0DB63ZZ or 0DB64ZZ or 0DB67ZZ or 0DB68ZZ) AND (0D160ZA or 0D164ZA or 0D168ZA) (excision stomach, bypass stomach to jejunum),(0DT60ZZ or 0DT64ZZ or 0DT67ZZ or 0DT68ZZ) AND (0D13079 or 0D1307A or 0D1307B) (resection stomach, bypass lower esophagus to duodenum/jejunum/ ileum) |
| **(3) NDC, ICD-9 and ICD-10 Diagnosis and Procedure Codes Used to Define Baseline Comorbidities** | |
| **Category** | **NDC, ICD-9-CM and ICD-10-CM Diagnosis or Procedure Codes** |
| **(3A) BMI Category** | |
| Specific BMI Categories | **Patients were assigned to a category based on the most recently coded diagnosis (often on day of surgery)**<br>BMI < 30: ICD-9 Diagnosis Codes V85.0, V85.1, V85.2 or ICD-10 Diagnosis Codes Z68.1, Z68.2<br>BMI 30-39.9: ICD-9 Diagnosis Codes V85.3x or ICD-10 Diagnosis Codes Z68.3x<br>BMI 40-49.9: ICD-9 Diagnosis Codes V85.41, V85.42 or ICD-10 Diagnosis Codes Z68.41, Z68.42<br>BMI ≥60: ICD-9 Diagnosis Codes V85.44, V85.45 or ICD-10 Diagnosis Code Z68.44, Z68.45 |
| Non-specific Morbid Obesity | **Patients were assigned to the non-specific obesity category when no specific BMI code was present**<br>ICD-9 Diagnosis code 278.01or ICD-10 Diagnosis code E66.01 (morbid obesity) |
| **(3B) Gastro-Esophageal Reflux Disease (GERD)** | |
| Presence of Baseline GERD | **Patients were defined as having baseline GERD when they had ≥1 diagnosis in the 6 months before surgery**<br>ICD-9 Diagnosis codes 530.11(reflux esophagitis), 530.81(esophageal reflux), 78.71 (heartburn) or ICD-10 Diagnosis codes K21 (GERD unspecified), K210 (GERD with esophagitis), K219 (GERD without esophagitis), R12 (heartburn) |
| **(3C) Type 2 Diabetes** | |
| Presence of Baseline Type 2 Diabetes | **Patients were defined as having diabetes when (a) they had ≥ 1 type 2 diagnosis in the 6 months before surgery AND (b) >50% of all diabetes diagnoses were specific to type 2**<br>Type 2 Diabetes: ICD-9 Diagnosis codes 250.x0 (type 2 or unspecified diabetes, not stated as uncontrolled), 250.x2 (type 2 or unspecified diabetes, uncontrolled) or ICD-10 Diagnosis codes E08xx (diabetes due to underlying condition), E09xx (drug or chemical induced diabetes), E11xx (type 2 diabetes), E13xx (other specified diabetes)<br>Other codes used to determine if ≥50% of diabetes diagnoses were specific to Type 2: ICD-9 Diagnoses 250.x1 (type 1 diabetes, not stated as uncontrolled), 250.x3 (type 1 diabetes, uncontrolled) or ICD-10 Diagnosis codes E10xx (type 1 diabetes) |
| **(3D) History of Tobacco Use** | |
| Baseline History of Tobacco Use | **Patients were defined as having a history of tobacco use when they had any diagnoses, treatments, laboratory tests, or prescriptions for smoking cessation in the 6 months before surgery**<br>ICD-9 Diagnosis codes 3051 (tobacco use disorder), 649x (tobacco use disorder complicating pregnancy), 98984 (toxic effect of tobacco) or ICD-10 Diagnosis codes Z720 (problems related to lifestyle, tobacco use), F17xx (nicotine dependence), O9933x (tobacco use complicating pregnancy), T6521xx (toxic effect of chewing tobacco), T6522xx (toxic effect of tobacco cigarettes), T6529xx (toxic effect of other tobacco and nicotine)CPT codes 99406, 99407, G0436, G0437, or G9016 (smoking and tobacco cessation counseling), S9453 (smoking cessation classes), S4995 (smoking cessation gum), G9276 or G9458 (documented tobacco user advised to quit), 1034F (current smoker), 4004F or 4001F (screened for tobacco use and received an intervention), 80323 (nicotine and metabolite, quantitative, urine)<br>LOINC codes 12294-5,28555-1 ,3853-9 ,3854-7 ,40386-5,40387-3,43125-4 ,43195-7,43218-7,4350-5 ,43830-9,55557-3 ,55558-1 , 59936-5 , 59937-3 , 60320-9 , 6502-0, 62503-8, 80541-6,87596-3,90226-2 (nicotine labs)<br>NDCs in First Data Bank AHFS6 category 129200 (Nicotrol, nicotine patch, nicotine tartrate, Nicorette, nicotine gum, Chantix) |
| **(3E) Other Health Conditions Flagged by ACG Software** | |
| **Patients were defined as having:** | **When the ACG software flagged any of the following EDC diagnosis groups in the 6 months before surgery:** |
| Psychiatric Illness | PSY01 (Anxiety, neuroses), PSY02 (Substance use), PSY07 (Schizophrenia), PSY08 (Personality disorders), PSY09 (Depression), PSY10(Psychologic signs and symptoms), PSY12 (Bipolar disorder), PSY13 (Adjustment disorder), PSY14 (Psychosocial disorders of childhood), PSY15 (Eating disorder), PSY16 (Impulse control), PSY17(Psycho- physiologic and somatoform disorders), PSY18 (Psychosexual), PSY20 (Major depression) |
| Cardiovascular Disease | CAR03 (Ischemic heart disease, excluding acute myocardial infarction), CAR05 (Congestive heart failure), CAR07 (Cardiomyopathy), CAR10 (Generalized atherosclerosis), CAR12 (Acute myocardial infarction), CAR16 (Cardiovascular disorders, other), GSU11 (Peripheral vascular disease), NUR05 (Cerebrovascular disease) |
| Hypertension | CAR14 (Hypertension, w/o major complications), CAR15 (Hypertension, with major complications) |
| Renal Disease | REN01 (Chronic renal failure), REN03 (Acute renal failure), REN04 (Nephritis, nephrosis), REN05 (Renal disorders, other), REN06 (End Stage Renal Disease) |
| Liver Disease | GAS04 (Acute hepatitis), GAS05 (Chronic liver disease), GAS14(Gastrointestinal/Hepatic disorders) |

© 2019 Lewis KH et al. *JAMA Network Open*.

**eTable 4.** Sensitivity Results From Cox Proportional Hazards Models[a] Comparing Matched Cohorts of VSG[b] and RYGB[c] Patients, Up to 48 Months After Surgery, and Procedure-Specific Estimated Event Rates Based on Kaplan-Meier Plots--Using CPT Codes Only to Define Events

| Outcome Measure | Adjusted Cox Model | | Cumulative Incidence of Outcome (95% CI) for VSG[b] and RYGB[c] in the Early (90 days), Mid (1 year) and Later (4 years) Post-operative Periods[d] | | | | | |
|---|---|---|---|---|---|---|---|---|
| | Hazard Ratio for VSG[b] vs. RYGB[c] (95% CI) | p-value | 90 Days After Index Procedure | | 1 Year After Index Procedure | | 4 Years After Index Procedure | |
| | | | RYGB[c] | VSG[b] | RYGB[c] | VSG[b] | RYGB[c] | VSG[b] |
| **Remains enrolled No./eligible No. (%)** | **N/A** | **N/A** | 4098/4384 (93.5%) | 7900/8382 (94.2%) | 2825/3941 (71.7%) | 5743/7538 (76.2%) | 701/2362 (29.7%) | 1420/4395 (32.3%) |
| **Overall Abdominal Operative Intervention[e]** | **0.79 (0.71, 0.88)** | **p<0.001** | 2.3% (2.9%, 2.8%) | 2.3% (2.0%, 2.7%) | 8.6% (7.8% ,9.6%) | 6.2% (5.7%, 6.8%) | 20.7% (19.0%,22.6%) | 17.4% (16.2%,18.7%) |
| **Biliary Procedure[f]** | **0.75 (0.65, 0.88)** | **p<0.001** | 0.7% (0.5%, 1.0%) | 0.5% (0.4%, 0.7%) | 4.6% (3.9%, 5.4%) | 3.2% (2.9%, 3.7%) | 11.1% (9.8%, 12.6%) | 8.8% (7.9%, 9.7%) |
| **Abdominal Wall Hernia Repair[g]** | **0.59 (0.46, 0.76)** | p<0.001 | 0.0% (0.0%, 0.2%) | 0.0% (0.0%, 0.1%) | 1.4% (1.0%, 1.9%) | 0.8% (0.7%, 1.1%) | 5.9% (4.8%, 7.2%) | 3.4% (2.8%, 4.1%) |
| **Bariatric Conversion or Revision[h]** | **2.23 (1.35, 3.67)** | **p=0.002** | 0.1% (0.0%, 0.3%) | 0.2% (0.2%, 0.4%) | 0.3% (0.2%, 0.5%) | 0.5% (0.4%, 0.7%) | 0.7% (0.4%, 1.1%) | 2.1% (1.7%, 2.7%) |
| **Other Abdominal Operation[i]** | **0.68 (0.57, 0.81)** | **p<0.001** | 1.5% (1.2%, 2.0%) | 1.6% (1.4%, 1.9%) | 3.8% (3.2%, 4.4%) | 2.3% (2.0%, 2.7%) | 8.8% (7.7%, 10.2%) | 6.5% (5.8%, 7.4%) |
| **Endoscopy[j]** | **0.53 (0.48, 0.58)** | **p<0.001** | 8.5% (7.9%, 9.5%) | 3.2% (2.9%, 3.7%) | 15.2% (14.1%,16.4%) | 6.9% (6.3% ,7.5%) | 26.0% (24.2%,27.9%) | 18.1% (16.8%,19.4%) |
| **Enteral Access[k]** | 0.67 (0.43, 1.05) | p=0.08 | 0.4% (0.2%, 0.6%) | 0.4% (0.3%, 0.6%) | 0.7% (0.5%, 1.0%) | 0.5% (0.4%, 0.7%) | 1.0% (0.7%, 1.5%) | 0.6% (0.4%, 0.7%) |
| **Other Non-Operative Intervention[l]** | 0.90 (0.62, 1.31) | p=0.57 | 0.4% (0.3%, 0.7%) | 0.4% (0.3%, 0.6%) | 0.8% (0.6%, 1.1%) | 0.6% (0.4%, 0.7%) | 1.5% (1.1%, 2.2%) | 1.5% (1.2%, 1.9%) |

*a -Models were adjusted for all matched covariates plus age group, sex, baseline ACG comorbidity score group, and presence of GERD, hypertension and mental illness. b- vertical sleeve gastrectomy; c- roux en y gastric bypass; d- From adjusted Kaplan-Meier plots at days 90, 360 and 1440 relative to index procedure; e - Category includes any operative reoperation on the abdomen (includes subcategories of biliary procedures, abdominal wall hernia repairs, conversions/revisions and reoperation) Complete code listing can be found in Table A1 of this Appendix; f -Category includes only procedures on the biliary tract such as cholecystectomy and placement of drains in the biliary tree; g- Category includes only repair of ventral hernias and other abdominal wall hernias - does not include internal hernias or paraesophageal hernias; h - Category includes only subsequent bariatric procedures (e.g. conversion from VSG to RYGB) as well as revisional procedures such as gastrectomy; i - Category includes those abdominal operative procedures not captured under the categories of biliary, abdominal wall hernias, or conversion/revision, and represents presumed complications; j - Category includes any endoscopic procedure for diagnosis or treatment on the upper GI tract; k - Category includes placement of gastrostomy tubes or other feeding devices, either percutaneously or through other means of access; l - Category includes invasive but not operative procedures on the abdomen such as paracentesis, or radiologically-guided drainage procedures that do not involve incisions.*

© 2019 Lewis KH et al. *JAMA Network Open*.

**eTable 5.** Results From Cox Proportional Hazards Models[a] Comparing Unmatched Cohorts of VSG[b] and RYGB[c] Patients, Up to 48 Months After Surgery, and Procedure-Specific Estimated Event Rates Based on Kaplan-Meier Plots

| Outcome Measure | Adjusted Cox Model | | Cumulative Incidence of Outcome (95% CI) for VSG[b] and RYGB[c] in the Early (90 days), Mid (1 year) and Later (4 years) Post-operative Periods[d] | | | | | |
|---|---|---|---|---|---|---|---|---|
| | Hazard Ratio for VSG[b] vs. RYGB[c] (95% CI) | p-value | 90 Days After Index Procedure | | 1 Year After Index Procedure | | 4 Years After Index Procedure | |
| | | | RYGB[c] | VSG[b] | RYGB[c] | VSG[b] | RYGB[c] | VSG[b] |
| **Remains enrolled No./eligible No. (%)** | **N/A** | **N/A** | 4117/4404 (93.5%) | 7813/8331 (93.8%) | 2838/3960 (71.7%) | 5138/6873 (74.8%) | 707/2376 (29.8%) | 728/2322 (31.4%) |
| **Overall Abdominal Operative Intervention[e]** | **0.77 (0.68, 0.86)** | **p<0.001** | 2.9% (2.5%, 3.5%) | 2.5% (2.2%, 2.9%) | 9.3% (8.4% ,10.3%) | 6.7% (6.2%, 7.4%) | 21.9% (20.1%,23.8%) | 17.3% (15.9%,18.9%) |
| **Biliary Procedure[f]** | **0.79 (0.68, 0.93)** | **p=0.005** | 0.7% (0.5%, 1.0%) | 0.7% (0.5%, 0.9%) | 4.6% (4.0%, 5.3%) | 3.5% (3.1%, 4.0%) | 11.3% (10.0%, 12.8%) | 9.5% (8.4%, 10.7%) |
| **Abdominal Wall Hernia Repair[g]** | **0.51 (0.39, 0.66)** | **p<0.001** | 0.1% (0.0%, 0.2%) | 0.0% (0.0%, 0.1%) | 1.7% (1.3%, 2.2%) | 1.0% (0.8%, 1.2%) | 6.4% (5.3%, 7.7%) | 3.2% (2.5%, 4.1%) |
| **Bariatric Conversion or Revision[h]** | **1.51 (0.94, 2.43)** | **p=0.089** | 0.2% (0.1%, 0.3%) | 0.2% (0.1%, 0.3%) | 0.4% (0.2%, 0.6%) | 0.5% (0.3%, 0.7%) | 1.1% (0.7%, 1.7%) | 2.0% (1.4%, 2.8%) |
| **Other Abdominal Operation[i]** | **0.65 (0.55, 0.77)** | **p<0.001** | 2.2% (1.8%, 2.6%) | 1.8% (1.6%, 2.2%) | 4.8% (4.1%, 5.5%) | 2.9% (2.5%, 3.3%) | 10.6% (9.3%, 12.0%) | 6.6% (5.8%, 7.7%) |
| **Endoscopy[j]** | **0.53 (0.48, 0.59)** | **p<0.001** | 9.0% (8.3% ,10.0%) | 4.0% (3.7%, 4.5%) | 15.6% (14.5%,16.8%) | 7.4% (6.8% ,8.0%) | 26.5% (24.6%,28.4%) | 18.9% (17.3%,20.5%) |
| **Enteral Access[k]** | **0.36 (0.22, 0.59)** | **p<0.001** | 0.5% (0.4%, 0.8%) | 0.2% (0.1%, 0.3%) | 0.9% (0.7%, 1.3%) | 0.3% (0.2%, 0.4%) | 1.5% (1.1%, 2.1%) | 0.4% (0.3%, 0.6%) |
| **Other Non-Operative Intervention[l]** | 1.00 (0.68, 1.46) | p=0.993 | 0.5% (0.4%, 0.8%) | 0.4% (0.3%, 0.6%) | 0.9% (0.6%, 1.2%) | 0.6% (0.5%, 0.8%) | 1.7% (1.2%, 2.3%) | 1.7% (1.3%, 2.3%) |

***a** Models were adjusted for age group, sex, baseline ACG comorbidity score group, BMI category, calendar year group, US region, and presence of diabetes, GERD, hypertension and mental illness; **b**-vertical sleeve gastrectomy; **c**- roux en y gastric bypass; **d**- From adjusted Kaplan-Meier plots at days 90, 360 and 1440 relative to index procedure; **e** - Category includes any operative reoperation on the abdomen (includes subcategories of biliary procedures, abdominal wall hernia repairs, conversions/revisions and reoperation) Complete code listing can be found in Table A1 of this Appendix; **f** -Category includes only procedures on the biliary tract such as cholecystectomy and placement of drains in the biliary tree; **g**- Category includes only repair of ventral hernias and other abdominal wall hernias - does not include internal hernias or paraesophageal hernias; **h** - Category includes only subsequent bariatric procedures (e.g. conversion from VSG to RYGB) as well as revisional procedures such as gastrectomy; **i** - Category includes those abdominal operative procedures not captured under the categories of biliary, abdominal wall hernias, or conversion/revision, and represents presumed complications; **j** - Category includes any endoscopic procedure for diagnosis or treatment on the upper GI tract; **k** - Category includes placement of gastrostomy tubes or other feeding devices, either percutaneously or through other means of access; **l** - Category includes invasive but not operative procedures on the abdomen such as paracentesis, or radiologically-guided drainage procedures that do not involve incisions.*

© 2019 Lewis KH et al. *JAMA Network Open*.

**eTable 6.** Number (%) of Patients Remaining Enrolled Over Postoperative Follow-up Period, by Surgery Type

| | Surgery Day | 30 Days | 90 Days | 180 Days | 360 Days | 720 Days | 1080 Days | 1440 Days |
|---|---|---|---|---|---|---|---|---|
| | Number Eligible[a] | | | | | | | |
| **RYGB[b]** | 4476 (100%) | 4471 (99.9%) | 4384 (97.9%) | 4281 (95.6%) | 3941 (88.0%) | 3376 (75.4%) | 2905 (64.9%) | 2362 (52.8%) |
| **VSG[c]** | 8551 (100%) | 8549(99.9%) | 8382 (98.0%) | 8117 (95.6%) | 7538(88.2%) | 6426 (75.1%) | 5480 (64.1%) | 4395 (51.4%) |
| | Number (%) Enrolled[d] | | | | | | | |
| **RYGB[b]** | 4476 (100%) | 4466 (99.9%) | 4098 (93.5%) | 3621 (84.6%) | 2825 (71.7%) | 1773 (52.5%) | 1141 (39.3%) | 701 (29.7%) |
| **VSG[c]** | 8551 (100%) | 8533 (99.8%) | 7900 (94.2%) | 7082 (86.6%) | 5743 (76.2%) | 3679 (57.3%) | 2364 (43.1%) | 1420 (32.3%) |

*a- Counts are CEM-weighted and represent patients whose index surgery was at least these many days from the end of our data (6/30/2017). b- roux en y gastric bypass. c - vertical sleeve gastrectomy. d- Counts are CEM-weighted and represent n (or %) of eligible patients who were alive, enrolled, <65 years old and had no GI malignancy diagnoses.*

**eTable 7. Number (%) of Patients Remaining Enrolled in Unmatched Cohort, Over Postoperative Period, by Surgery Type**

| | Surgery Day | 30 Days | 90 Days | 180 Days | 360 Days | 720 Days | 1080 Days | 1440 Days |
|---|---|---|---|---|---|---|---|---|
| | Number Eligible[a] | | | | | | | |
| **RYGB[b]** | 4496 (100%) | 4491 (99.9%) | 4404 (98.0%) | 4301 (95.7%) | 3960 (88.1%) | 3392 (75.4%) | 2920 (64.9%) | 2376 (52.8%) |
| **VSG[c]** | 8627 (100%) | 8624 (99.9%) | 8331 (96.6%) | 7981 (92.5%) | 6873 (79.7%) | 5038 (58.4%) | 3586 (42.6%) | 2322 (26.9%) |
| | Number (%) Enrolled[d] | | | | | | | |
| **RYGB[b]** | 4496 (100%) | 4486 (99.9%) | 4117 (93.5%) | 3637 (84.6%) | 2838 (71.7%) | 1783 (52.6%) | 1148 (39.3%) | 707 (29.8%) |
| **VSG[c]** | 8627 (100%) | 8605 (99.8%) | 7813 (93.8%) | 6829 (85.6%) | 5138 (74.8%) | 2802 (55.6%) | 1505 (42.0%) | 728 (31.4%) |

*a- Counts represent patients whose index surgery was at least these many days from the end of our data (6/30/2017). b- Roux en y gastric bypass. c- vertical sleeve gastrectomy. d- Counts represent n (or %) of eligible patients who were alive, enrolled, <65 years old and had no GI malignancy diagnoses.*

© 2019 Lewis KH et al. *JAMA Network Open*.

**eFigure 1.** Time to Operative Abdominal Intervention, Endoscopy, Other Abdominal Operation, or Bariatric Conversion or Revision in Matched Cohorts of RYGB[a] and VSG[b] Patients, Using Only CPT[c] Codes to Define Event

**A. Overall Operative Abdominal Intervention[d]**

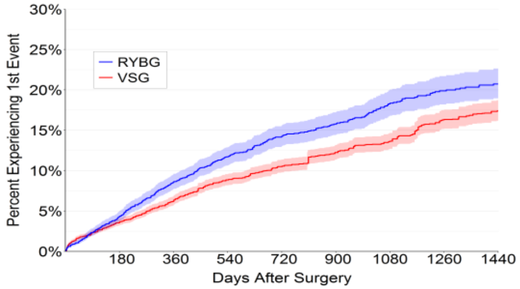


**Number (%) at Risk[h]**

| | Surgery Day | 360 Days | 720 Days | 1440 Days |
|---|---|---|---|---|
| **RYGB** | 4476 (100%) | 2577 (65.4%) | 1520 (45.0%) | 566 (24.0%) |
| **VSG** | 8551 (100%) | 5380 (71.4%) | 3275 (51.0%) | 1169 (26.6%) |

**B. Endoscopy[e]**

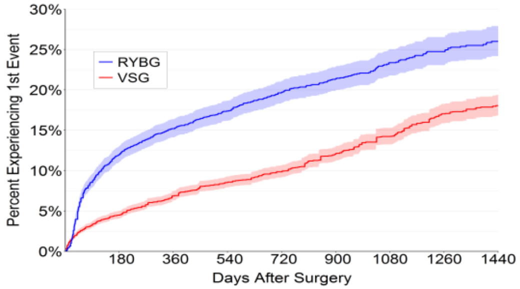


**Number (%) at Risk[h]**

| | Surgery Day | 360 Days | 720 Days | 1440 Days |
|---|---|---|---|---|
| **RYGB** | 4476 (100%) | 2415 (61.3%) | 1450 (43.0%) | 554 (23.5%) |
| **VSG** | 8551 (100%) | 5368 (71.2%) | 3336 (51.9%) | 1187 (27.0%) |

**C. Other Abdominal Operation[f]**

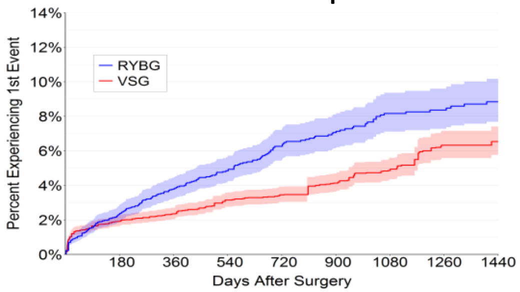


**Number (%) at Risk[h]**

| | Surgery Day | 360 Days | 720 Days | 1440 Days |
|---|---|---|---|---|
| **RYGB** | 4476 (100%) | 2717 (68.9%) | 1660 (49.2%) | 645 (27.3%) |
| **VSG** | 8551 (100%) | 5594 (74.2%) | 3540 (55.1%) | 1316 (29.9%) |

**D. Bariatric Conversion or Revision[g]**

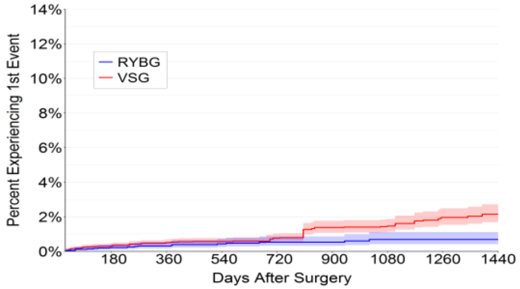


**Number (%) at Risk[h]**

| | Surgery Day | 360 Days | 720 Days | 1440 Days |
|---|---|---|---|---|
| **RYGB** | 4476 (100%) | 2815 (71.4%) | 1762 (52.2%) | 695 (29.4%) |
| **VSG** | 8551 (100%) | 5713 (75.8%) | 3653 (56.9%) | 1385 (31.5%) |

***a-*** *roux en y gastric bypass**;** **b-** vertical sleeve gastrectomy**;** **c-** current procedural technology**;** **d** - Category includes any operative reoperation on the abdomen (includes subcategories of biliary procedures, abdominal wall hernia repairs, conversions/revisions and reoperation) Complete code listing can be found in Table A1 of this Appendix; **e** - Category includes any endoscopic procedure for diagnosis or treatment on the upper GI tract; **f** - Category includes those abdominal operative procedures not captured under the categories of biliary, abdominal wall hernias, or conversion/revision, and represents presumed complications; **g** - Category includes only subsequent bariatric procedures (e.g. conversion from VSG to RYGB) as well as revisional procedures such as gastrectomy; ; **h-** Counts are CEM-weighted and represent patients who remain enrolled and at-risk (have not yet had an event of interest) at each time point. Note that because many of the procedures took place in the later years of our data, some proportion of both VSG and RYGB patients lack complete follow-up not because of loss to follow-up / disenrollment or events, but rather because of insufficient time between the date of their surgery and the end of our dataset. To more accurately represent completeness of follow-up accounting for this fact, **eTable6** in the Supplement provides counts and percentage enrolled relative to those truly eligible for complete follow-up at all relevant time points.*

© 2019 Lewis KH et al. *JAMA Network Open*.

**eFigure 2.** Time to First Biliary Procedure, Abdominal Wall Hernia Repair, Enteral Access, or Other Nonoperative Intervention in Matched Cohorts of RYGB[a] and VSG[b] Patients, Using Only CPT[c] Codes to Define Event

**A. Biliary Procedure[d]**

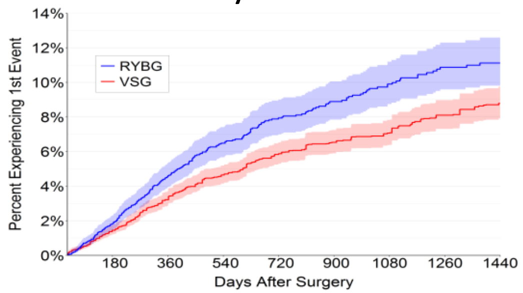


**Number (%) at Risk[h]**

| | Surgery Day | 360 Days | 720 Days | 1440 Days |
|---|---|---|---|---|
| **RYGB** | 4476 (100%) | 2693(68.3%) | 1642(48.6%) | 625(26.5%) |
| **VSG** | 8551 (100%) | 5557(73.7%) | 3462(53.9%) | 1290(29.4%) |

**B. Abdominal Wall Hernia Repair[e]**

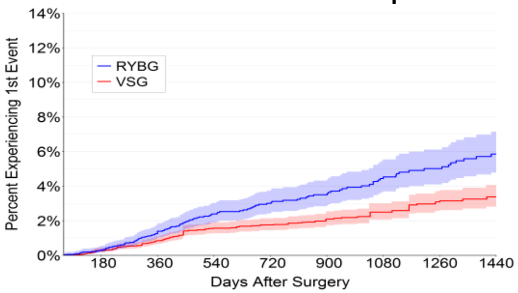


**Number (%) at Risk[h]**

| | Surgery Day | 360 Days | 720 Days | 1440 Days |
|---|---|---|---|---|
| **RYGB** | 4476 (100%) | 2786(70.7%) | 1714(50.8%) | 671(28.4%) |
| **VSG** | 8551 (100%) | 5699(75.6%) | 3611(56.2%) | 1383(31.5%) |

**C. Enteral Access[f]**

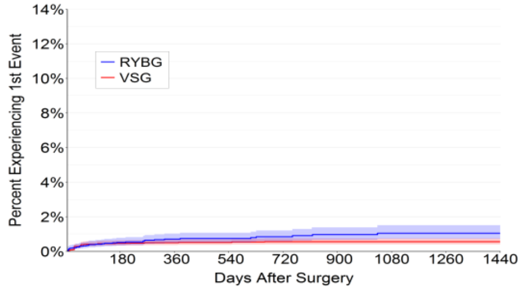


**Number (%) at Risk[h]**

| | Surgery Day | 360 Days | 720 Days | 1440 Days |
|---|---|---|---|---|
| **RYGB** | 4476 (100%) | 2807(71.2%) | 1755(52.0%) | 694(29.4%) |
| **VSG** | 8551 (100%) | 5707(75.7%) | 3645(56.7%) | 1415(32.2%) |

**D. Other Non-operative Intervention[g]**

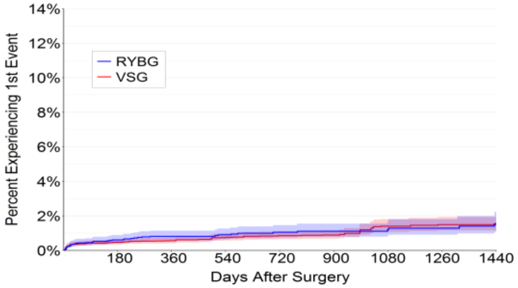


**Number (%) at Risk[h]**

| | Surgery Day | 360 Days | 720 Days | 1440 Days |
|---|---|---|---|---|
| **RYGB** | 4476 (100%) | 2804(71.1%) | 1754(52.0%) | 694(29.4%) |
| **VSG** | 8551 (100%) | 5706(75.7%) | 3643(56.7%) | 1397(31.8%) |

***a-*** *roux en y gastric bypass;* ***b –*** *vertical sleeve gastrectomy;* ***c-*** *current procedural technology;* ***d-*** *Category includes only procedures on the biliary tract such as cholecystectomy and placement of drains in the biliary tree;* ***e-****Category includes only repair of ventral hernias and other abdominal wall hernias - does not include internal hernias or paraesophageal hernias;* ***f-*** *Category includes placement of gastrostomy tubes or other feeding devices, either percutaneously or through other means of access;* ***f g*** *- Category includes invasive but non-operative procedures on the abdomen such as paracentesis, or radiologically-guided drainage procedures that do not involve incisions; ;* ***h-*** *Counts are CEM-weighted and represent patients who remain enrolled and at-risk (have not yet had an event of interest) at each time point. Note that because many of the procedures took place in the later years of our data, some proportion of both VSG and RYGB patients lack complete follow-up not because of loss to follow-up / disenrollment or events, but rather because of insufficient time between the date of their surgery and the end of our dataset. To more accurately represent completeness of follow-up accounting for this fact,* ***eTable6*** *in the Supplement provides counts and percentage enrolled relative to those truly eligible for complete follow-up at all relevant time points.*

© 2019 Lewis KH et al. *JAMA Network Open*.

**eFigure 3.** Time to First Operative Abdominal Intervention, Endoscopy, Other Abdominal Operation. or Bariatric Conversion or Revision in Unmatched Cohorts of RYGB[a] and VSG[b] Patients

### A. Overall Operative Abdominal Intervention[c]

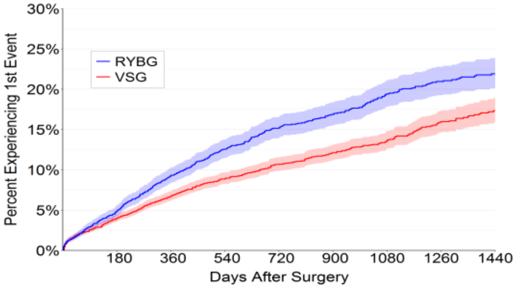


**Number (%) at Risk[g]**

| | Surgery Day | 360 Days | 720 Days | 1440 Days |
|---|---|---|---|---|
| **RYGB** | 4496 (100%) | 2570 (64.9%) | 1516 (44.7%) | 562 (23.7%) |
| **VSG** | 8626 (100%) | 4791 (69.7%) | 2500 (49.6%) | 604 (26.0%) |

### B. Endoscopy[d]

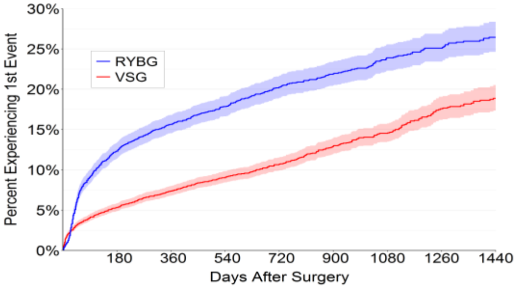


**Number (%) at Risk[g]**

| | Surgery Day | 360 Days | 720 Days | 1440 Days |
|---|---|---|---|---|
| **RYGB** | 4496 (100%) | 2415 (61.0%) | 1449 (42.7%) | 550 (23.1%) |
| **VSG** | 8626 (100%) | 4763 (69.3%) | 2505 (49.7%) | 605 (26.1%) |

### C. Other Abdominal Operation[e]

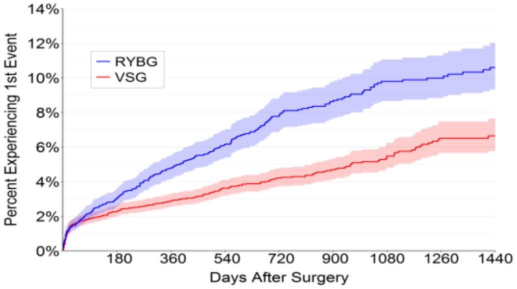


**Number (%) at Risk[g]**

| | Surgery Day | 360 Days | 720 Days | 1440 Days |
|---|---|---|---|---|
| **RYGB** | 4496 (100%) | 2701 (68.2%) | 1648 (48.6%) | 638 (26.9%) |
| **VSG** | 8626 (100%) | 4980 (72.5%) | 2675 (53.1%) | 670 (28.9%) |

### D. Bariatric Conversion or Revision[f]

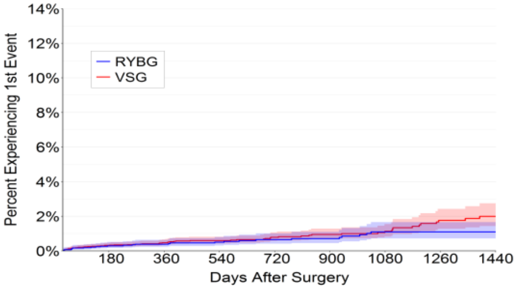


**Number (%) at Risk[g]**

| | Surgery Day | 360 Days | 720 Days | 1440 Days |
|---|---|---|---|---|
| **RYGB** | 4496 (100%) | 2827 (71.4%) | 1770 (52.2%) | 701 (29.5%) |
| **VSG** | 8626 (100%) | 5115 (74.4%) | 2781 (55.2%) | 711 (30.6%) |

*__a__ -roux en y gastric bypass; __b__-vertical sleeve gastrectomy; __c__ - Category includes any operative reoperation on the abdomen (includes subcategories of biliary procedures, abdominal wall hernia repairs, conversions/revisions and reoperation) Complete code listing can be found in Table A1 of this Appendix; __d__ - Category includes any endoscopic procedure for diagnosis or treatment on the upper GI tract; __e__ - Category includes those abdominal operative procedures not captured under the categories of biliary, abdominal wall hernias, or conversion/revision, and represents presumed complications; __f__ - Category includes only subsequent bariatric procedures (e.g. conversion from VSG to RYGB) as well as revisional procedures such as gastrectomy; __g-__ Counts represent patients who remain enrolled and at-risk (have not yet had an event of interest) at each time point. Note that because many of the procedures took place in the later years of our data, some proportion of both VSG and RYGB patients lack complete follow-up not because of loss to follow-up / disenrollment or events, but rather because of insufficient time between the date of their surgery and the end of our dataset. To more accurately represent completeness of follow-up accounting for this fact, __eTable7__ in the Supplement provides counts and percentage enrolled relative to those truly eligible for complete follow-up at all relevant time points.*

© 2019 Lewis KH et al. *JAMA Network Open*.

**eFigure 4**. Time to First Biliary Procedure, Abdominal Wall Hernia Repair, Enteral Access, or Other Nonoperative Intervention in Unmatched Cohorts of RYGB[a] and VSG[b] Patients

### A. Biliary Procedure[c]

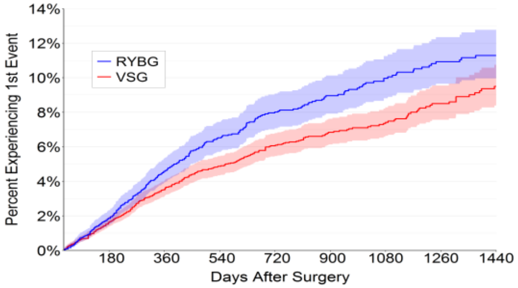


**Number (%) at Risk[g]**

| | Surgery Day | 360 Days | 720 Days | 1440 Days |
|---|---|---|---|---|
| **RYGB** | 4496 (100%) | 2705 (68.3%) | 1649 (48.6%) | 628 (26.4%) |
| **VSG** | 8626 (100%) | 4962 (72.2%) | 2638 (52.4%) | 667 (28.7%) |

### B. Abdominal Wall Hernia Repair[d]

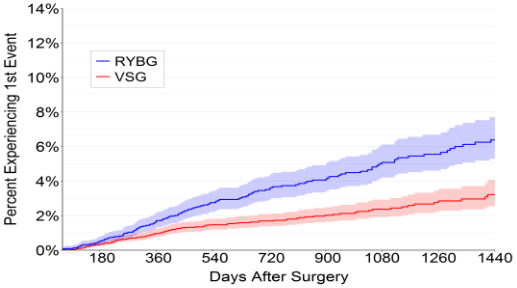


**Number (%) at Risk[g]**

| | Surgery Day | 360 Days | 720 Days | 1440 Days |
|---|---|---|---|---|
| **RYGB** | 4496 (100%) | 2791 (70.5%) | 1714 (50.5%) | 674 (28.4%) |
| **VSG** | 8626 (100%) | 5088 (74.0%) | 2753 (54.6%) | 708 (30.5%) |

### C. Enteral Access[e]

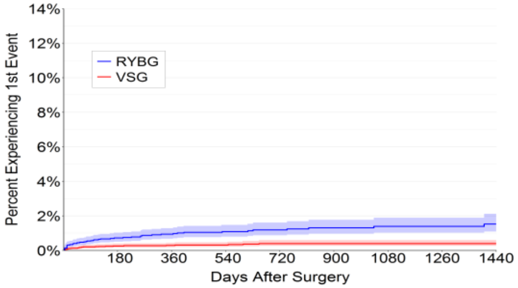


**Number (%) at Risk[g]**

| | Surgery Day | 360 Days | 720 Days | 1440 Days |
|---|---|---|---|---|
| **RYGB** | 4496 (100%) | 2817 (71.1%) | 1762 (51.9%) | 698 (29.4%) |
| **VSG** | 8626 (100%) | 5122 (74.9%) | 2788 (55.3%) | 725 (31.2%) |

### D. Other Non-operative Intervention[f]

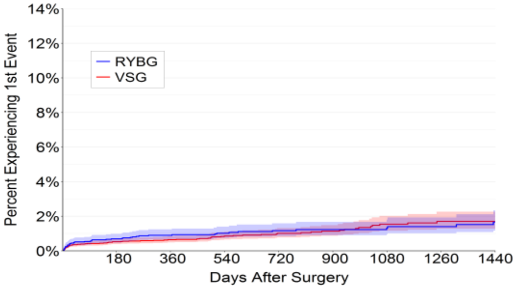


**Number (%) at Risk[g]**

| | Surgery Day | 360 Days | 720 Days | 1440 Days |
|---|---|---|---|---|
| **RYGB** | 4496 (100%) | 2813 (71.0%) | 1762 (51.9%) | 699 (29.4%) |
| **VSG** | 8626 (100%) | 5100 (74.2%) | 2774 (55.1%) | 715 (30.8%) |

***a-*** *roux en y gastric bypass****;*** ***b-*** *vertical sleeve gastrectomy****;*** ***c -*** *Category includes only procedures on the biliary tract such as cholecystectomy and placement of drains in the biliary tree;* ***d-*** *Category includes only repair of ventral hernias and other abdominal wall hernias - does not include internal hernias or paraesophageal hernias;* ***e-*** *Category includes placement of gastrostomy tubes or other feeding devices, either percutaneously or through other means of access;* ***f -*** *Category includes invasive but non-operative procedures on the abdomen such as paracentesis, or radiologically-guided drainage procedures that do not involve incisions;.* ***g-*** *Counts represent patients who remain enrolled and at-risk (have not yet had an event of interest) at each time point. Note that because many of the procedures took place in the later years of our data, some proportion of both VSG and RYGB patients lack complete follow-up not because of loss to follow-up / disenrollment or events, but rather because of insufficient time between the date of their surgery and the end of our dataset. To more accurately represent completeness of follow-up accounting for this fact,* ***eTable7*** *in the Supplement provides counts and percentage enrolled relative to those truly eligible for complete follow-up at all relevant time points.*

© 2019 Lewis KH et al. *JAMA Network Open*.
